# Suffering in silence: Stigma, healthcare barriers, and resilience during Sierra Leone’s 2025 clade IIb mpox outbreak—A multi-perspective qualitative study

**DOI:** 10.1371/journal.pgph.0006686

**Published:** 2026-06-30

**Authors:** Eric Nzirakaindi Ikoona, Lucy Namulemo, Mary Magdalene Sinnah, Mohamed Alex Vandi, Foday Sahr

**Affiliations:** 1 National Public Health Agency, Freetown, Sierra Leone; 2 Foothills Community-Based Interventions, Monticello, Kentucky, United States of America; 3 Uganda Counselling and Support Services, Kampala, Uganda; Institute of Development Studies, UNITED KINGDOM OF GREAT BRITAIN AND NORTHERN IRELAND

## Abstract

Mpox was confirmed in Sierra Leone in January 2025, with a public health emergency declared shortly thereafter. By June 2025, the country reported over 4,000 confirmed cases caused by clade IIb. Limited qualitative research has explored the lived experiences of affected populations during mpox outbreaks in African settings. This study explored multi-stakeholder experiences of mpox, including illness trajectories, stigma manifestations, healthcare-seeking behaviours, and health system response challenges. We conducted a multi-perspective qualitative study using thematic framework analysis between March and August 2025 across four districts. Participants included mpox survivors (n = 42), healthcare workers (n = 38), community members (n = 36), and contact tracers (n = 24). Data were collected through 94 semi-structured interviews and 12 focus group discussions. Analysis was guided by a modified Social Ecological Model (SEM) integrating the Health Stigma and Discrimination Framework (HSDF). Five interconnected themes emerged: (1) illness characterised by physical suffering and lasting bodily changes; (2) multi-layered stigma operating across individual, interpersonal, community, and institutional levels; (3) healthcare-seeking shaped by fear of disclosure and economic constraints; (4) healthcare workers’ moral distress and professional isolation; (5) adaptive health system response alongside coordination challenges. Stigma operated as a cross-cutting dimension that intersected with all other themes, shaping illness experience, care-seeking behaviour, professional practice, and system-level response simultaneously. It also permeated all dimensions of the mpox outbreak experience, delaying care-seeking, undermining contact tracing, and compounding healthcare worker distress. Compound trauma from sequential epidemic exposure emerged as a distinct burden among frontline workers. These findings point to the need for stigma-informed outbreak response strategies, sustained healthcare worker support mechanisms, and community-centred communication approaches in post-conflict, resource-constrained settings facing recurrent epidemics.

## Introduction

Mpox (formerly monkeypox) remains a significant public health concern. The World Health Organization (WHO) declared the mpox upsurge a Public Health Emergency of International Concern (PHEIC) on 14 August 2024 [1]. Mpox was first confirmed in Sierra Leone on 10 January 2025, the outbreak was officially declared on 13 January 2025, and a national public health emergency was declared on 16 January 2025 [2,3]. By 22 June 2025, the country reported 4,294 confirmed cases and 28 deaths [4]. In early May 2025, Sierra Leone accounted for approximately half of Africa’s confirmed mpox cases, underscoring the intensity of transmission during the outbreak acceleration phase [5].

The Sierra Leone outbreak presents distinctive epidemiological and social characteristics. Genomic evidence indicates that most sequences fall within clade IIb and cluster as a distinct group descended from lineage A.2.2, consistent with sustained human-to-human transmission; a proposed designation identifies this newly observed Sierra Leone lineage as G.1 (alias of A.2.2.1) [6]. Frequent genital involvement among cases supports the plausibility of sexual networks contributing to clade IIb transmission in this setting [7,8]. Sexual exposure histories are socially and legally sensitive, and self-reported accounts of exposure have raised the possibility of underreporting, stigma, and disclosure constraints [8]. These epidemiological and social features carry significant implications for understanding disease spread, designing targeted interventions, and addressing the complex social dimensions of the outbreak.

### The Sierra Leone context

Sierra Leone’s mpox response occurs within a health system shaped by post-conflict rebuilding and the 2014–2016 Ebola epidemic, which disrupted services and workforce capacity [9,10]. More recently, the country navigated the COVID-19 pandemic while simultaneously addressing endemic challenges, including malaria, tuberculosis, and HIV [8]. These cumulative experiences have shaped both the health system’s adaptive capacity and community perceptions of outbreak response, creating a distinctive context in which trust, institutional memory, and resource constraints interact in ways that may differ from settings without comparable histories of sequential health emergencies.

The concentration of cases in the Western Area, encompassing Freetown and surrounding districts, reflects underlying patterns of urbanisation, population density, and healthcare access [4]. By mid-2025, four dedicated treatment centres had opened, a 400-bed facility had become operational in Freetown, and over 43,000 individuals had received vaccination— yet these resources remained insufficient for the scale of the epidemic [4,11].

### Stigma and infectious disease outbreaks

Infectious disease outbreaks have historically been accompanied by stigmatisation of affected individuals and communities [12,13]. Link and Phelan’s foundational conceptualisation describes stigma as occurring when elements of labelling, stereotyping, separation, status loss, and discrimination converge within a context of power imbalance [14]. Disease-related stigma manifests through multiple mechanisms: enacted stigma (actual experiences of discrimination), perceived stigma (expectations of mistreatment), internalised stigma (acceptance of negative societal attitudes), and associative stigma (stigma experienced by those connected to affected individuals) [15].

The mpox outbreak presents particular stigma challenges given its visible cutaneous manifestations, perceived associations with sexual transmission, and historical framings as an “African disease” [16,17]. Evidence from the 2022 global outbreak documented significant stigma among affected populations, including personalised stigma, negative self-image, and disclosure concerns [18,19]. Healthcare workers have similarly reported fear of contagion and social isolation [20,21]. While these dynamics are well documented in high-income country contexts, significant gaps remain in understanding how mpox- related stigma operates in African settings, where different cultural meanings, health system structures, and prior outbreak experiences may produce distinct stigma configurations [22].

### Healthcare workers’ experiences in outbreak response

Healthcare workers (HCWs) occupy a critical yet vulnerable position during infectious disease outbreaks [23]. Research from the 2022 mpox response documented knowledge gaps, with 30% of HCWs having never heard of mpox before the outbreak and over one- quarter initially misdiagnosing cases [24]. HCWs reported inadequate access to vaccines, concerns about occupational exposure, and moral distress related to resource constraints [25]. The psychological toll of outbreak response—compounded for many by the recent COVID-19 pandemic—has contributed to burnout, compassion fatigue, and professional attrition [26,27].

### Gaps in current knowledge

Despite growing epidemiological surveillance of mpox in Africa, qualitative research examining lived experiences remains markedly limited [28,29]. To our knowledge, no published qualitative studies have examined mpox experiences from endemic African settings during the 2024–2025 mpox PHEIC period. Existing qualitative research has predominantly emerged from high-income country contexts, primarily focusing on men who have sex with men populations affected by clade IIb during the 2022 outbreak [18,19,30]. This leaves significant knowledge gaps regarding how mpox illness is experienced in African contexts, the specific manifestations and mechanisms of mpox-related stigma in these settings, and healthcare-seeking behaviours and barriers in resource-constrained environments.

### Study aims and theoretical framework

This study aimed to provide a comprehensive qualitative exploration of mpox outbreak experiences in Sierra Leone, addressing the following research questions: (1) How do individuals who have experienced mpox understand and narrate their illness trajectories?

(2) What forms and mechanisms of stigma emerge during mpox outbreaks, and how do these affect individuals, families, and communities? (3) What factors shape healthcare-seeking behaviours among those affected by or at risk of mpox? (4) How do healthcare workers and public health personnel experience outbreak response? (5) How do experiences vary across different social positions and geographic contexts?

Our analysis was guided by a modified Social Ecological Model (SEM) integrating the Health Stigma and Discrimination Framework (HSDF) [15,31] (S1 Appendix). The SEM provides a multi-level structure for examining individual, interpersonal, community, organisational, and policy factors that shape health experiences. The HSDF offers a cross- cutting framework for understanding how stigma operates through marking, labelling, separation, status loss, and discrimination within specific contexts. We employed these frameworks as sensitising tools rather than rigid analytical templates, remaining open to findings that might challenge, extend, or complicate the frameworks’ assumptions— particularly regarding the interplay between structural constraints and individual-level processes in a post-conflict, resource-constrained setting.

## Methods

### Ethics statement

Ethical approval was obtained from the Sierra Leone Ethics and Scientific Review Committee (SLESRC-2025-049) on 28 February 2025. All participants provided written informed consent before participation. For participants who could not write, witnessed thumbprint consent was obtained in accordance with SLESRC guidance. The consent process covered the study purpose, procedures, voluntary participation, right to withdraw, confidentiality protections, potential emotional risks, referral options, audio-recording, data storage, dissemination plans, and contact information for the research team and ethics committee.

Given the potentially stigmatising nature of mpox, confidentiality protections were central to the study design. Participants selected interview locations that offered privacy; identifiers were removed during transcription; quotations are presented using pseudonyms and with identifying details altered; and data files are stored on encrypted, password-protected servers accessible only to the research team. Emotional distress protocols were established, and research assistants were trained to recognise distress signals and offer supportive responses. Referral pathways to mental health services were established in each district through a partnership with Partners in Health Sierra Leone. Participants received transportation reimbursement (NLE 50, approximately USD 2.50 at the June 2025 exchange rate) and refreshments, but no other compensation, to minimise coercion.

### Study design and period

We conducted a multi-perspective qualitative study using thematic framework analysis to examine lived experiences of the mpox outbreak [32,33]. The study was conducted between 10 March and 28 August 2025, spanning the outbreak’s acceleration, peak (May–June 2025), and initial decline phases. Thematic framework analysis is well-suited for policy-relevant health research involving multiple participant groups, enabling systematic comparison across cases while maintaining analytic transparency [32,34]. Our analytical approach was informed by the SEM and the HSDF, which provided sensitising concepts for theme development [15,31].

We incorporated multiple stakeholder perspectives to triangulate and develop a comprehensive understanding of the outbreak experience. This multi-perspective approach aligns with calls for qualitative research that moves beyond single-stakeholder views to capture the relational and systemic dimensions of health phenomena [35]. The four participant groups—survivors, healthcare workers, community members, and contact tracers—occupy distinct epistemic positions. Survivors provide experiential knowledge grounded in embodied illness experience; healthcare workers contribute professional and observational accounts shaped by clinical training and institutional roles; community members offer perspectives on social responses and collective meanings; and contact tracers provide operational insights from the interface between surveillance systems and community engagement. We treated these perspectives as complementary but epistemically distinct, and our analytical approach was designed to differentiate between lived experience and professional or observational discourse (see Data analysis). The study followed the Consolidated Criteria for Reporting Qualitative Research (COREQ) guidelines [36] (Table A in S2 Appendix).

### Setting

The study was conducted across four districts representing diverse epidemiological and geographic contexts: Western Area Urban (Freetown), Western Area Rural, Bo, and Kenema. These districts were selected based on: (1) case burden, with Western Area accounting for approximately 80% of confirmed cases; (2) geographic diversity, including urban, peri-urban, and rural settings; (3) healthcare infrastructure variation, from tertiary facilities to community health centres; and (4) prior outbreak experience, with all districts having been affected by the 2014–2016 Ebola epidemic [9].

At the time of data collection, the four districts exhibited distinct epidemiological profiles. Western Area Urban reported the highest case concentration, driven by population density and social mixing patterns characteristic of the capital. Western Area Rural, while geographically proximate, exhibited different transmission dynamics associated with peri-urban settlement patterns and more limited healthcare access. Bo and Kenema districts in the south and east reported lower overall case counts but faced particular challenges related to diagnostic capacity, delayed specimen transport, and greater distances to treatment centres. Across all four districts, cases were predominantly among young adults aged 15–35 years, with male predominance in reported cases [7].

### Participants and sampling

We recruited 140 participants across four stakeholder groups: mpox survivors who had completed isolation (n = 42), healthcare workers involved in mpox response (n = 38), community members from affected areas (n = 36), and contact tracers/surveillance officers (n = 24). Purposive sampling with maximum variation was employed to ensure diversity across demographic characteristics (age, gender, education), geographic location (urban/peri-urban/rural), and for survivors, illness severity and time since recovery.

Inclusion criteria for survivors required: confirmed mpox diagnosis (laboratory or clinical), completion of isolation period, age 18 years or older, capacity to provide informed consent, and willingness to discuss experiences. Healthcare worker eligibility required direct involvement in mpox clinical care, laboratory services, or infection prevention and control. Community member eligibility required residence in a community with documented mpox cases. Contact tracers required current or recent employment in mpox surveillance activities.

### Participant recruitment and contact

Recruitment utilised multiple pathways tailored to each stakeholder group. For survivor participants, treatment centre staff at the four designated mpox treatment facilities (Connaught Hospital, 34 Military Hospital, Kenema Government Hospital, and Bo Government Hospital) provided study information sheets to patients nearing discharge. Patients who expressed interest were referred to research assistants, who contacted them via telephone 2–4 weeks post-discharge to confirm eligibility and schedule interviews. Community health workers also identified survivors in their catchment areas who had completed home-based isolation.

Healthcare workers were recruited through facility-based approaches. Research team members visited hospitals, peripheral health units, and laboratories involved in the mpox response to present the study at staff meetings. Interested staff provided contact information and were subsequently contacted to arrange interviews at convenient times. Contact tracers were recruited through District Health Management Teams (DHMTs); surveillance focal persons distributed study information during routine team meetings, and interested personnel were contacted directly by research assistants.

Community members were identified through community leaders and existing community health structures. Community Health Officers introduced the study at community meetings in areas with documented mpox cases. Interested community members provided contact details and were subsequently contacted to arrange participation. We assessed theme stability iteratively within each stakeholder group and district; recruitment stopped when additional interviews did not generate new codes or meaningfully extend existing themes.

### Data collection

Data collection employed semi-structured interviews (SSIs) (S3 Appendix) and focus group discussions (FGDs) (S4 Appendix), with methods matched to participant group, preferences, topic sensitivity, and feasibility. Individual SSIs were preferred for survivors because illness narratives, perceived sexual exposure, stigma, and family or workplace disclosure were highly sensitive. For healthcare workers, SSIs were used to elicit private accounts of moral distress, resource constraints, occupational risk, and emotional burden, while FGDs were used to explore shared operational experiences, collective reflections, and points of convergence or disagreement across professional roles.

In total, we conducted 94 SSIs (42 with survivors, 28 with healthcare workers, 12 with community members, 12 with contact tracers) and 12 FGDs (4 with healthcare workers, 6 with community members, 2 with contact tracers). FGDs comprised 6–10 participants each, grouped by stakeholder type and district to enable comfortable discussion among peers with shared experiences. For healthcare worker FGDs, we attempted to group participants by cadre or level of seniority where feasible, but full stratification was not always possible because of small numbers of eligible staff at some facilities, particularly in Bo and Kenema.

Because FGDs in clinical settings can suppress disclosure, facilitators monitored group dynamics, invited quieter participants to speak, reminded participants that disagreement was acceptable, and avoided questions that required disclosure of identifiable colleague or patient information. Where participants appeared uncomfortable or where sensitive issues required more private exploration, follow-up SSIs were offered. We therefore interpret healthcare worker FGDs as collective professional discourse and place greater evidentiary weight on SSIs for sensitive individual experiences.

Interview guides were developed separately for each stakeholder group, informed by the theoretical framework and existing literature (S3 and S4 Appendices). Survivor guides explored illness onset and trajectory, healthcare-seeking experiences, social responses and stigma, coping strategies, and perspectives on recovery. Healthcare worker guides examined clinical experiences, occupational challenges, resource constraints, emotional impacts, and recommendations. Community member guides explored knowledge and perceptions, community responses, fears and concerns, and recommendations. Contact tracer guides examined work experiences, challenges encountered, community responses, and support needs.

All guides were piloted with five individuals from each stakeholder group and refined based on feedback. Interviews were conducted in Krio, Mende, Temne, or English according to participant preference by trained Sierra Leonean research assistants fluent in the local languages used for the study. The research assistants were embedded in Sierra Leonean cultural contexts and explained study procedures and sensitive concepts in locally meaningful terms when needed. Interviews averaged 62 minutes for survivors (range: 45–95 minutes), 48 minutes for other SSIs (range: 35–75 minutes), and 87 minutes for FGDs (range: 72–110 minutes). All sessions were audio-recorded with permission and transcribed verbatim in the original language before being translated into English by bilingual team members.

Translations were verified by back-translating randomly selected segments (20% of transcripts).

### Informed consent process

The informed consent process was designed to ensure voluntary, informed participation while being sensitive to the stigmatising nature of mpox. Prior to each interview or FGD, research assistants met with potential participants in a private setting of their choosing (typically their home, a private room at a health facility, or a community centre).

Research assistants explained the study using a standardised information sheet available in English, Krio, Mende, and Temne.

The consent process covered: (1) study purpose and procedures, including the expected duration and topics to be discussed; (2) voluntary nature of participation, with explicit emphasis that declining would not affect access to healthcare or other services; (3) right to withdraw at any time without penalty; (4) right to skip any question or stop the interview at any time; (5) confidentiality protections, including use of pseudonyms, secure data storage, and removal of identifying information; (6) potential risks including emotional distress when discussing difficult experiences; (7) available support including referral to mental health services if needed; (8) audio-recording procedures and data storage; (9) plans for data use and dissemination; and (10) contact information for the research team and ethics committee for questions or concerns.

Participants were given time to ask questions and were provided with the information sheet to keep. For participants who could not read, the information sheet was read aloud in their preferred language. Written informed consent was obtained from all participants before data collection commenced. For participants who could not write, witnessed thumbprint consent was obtained in accordance with Sierra Leone Ethics and Scientific Review Committee guidelines. A copy of the signed consent form was provided to each participant.

### Research team and reflexivity

The research team comprised investigators with backgrounds in public health, medicine, nursing, medical anthropology, and community health. The study was locally led from within Sierra Leone’s public health system and institutionally anchored in the National Public Health Agency (NPHA), the national institution responsible for public health protection, surveillance, outbreak preparedness and response, and evidence generation to understand drivers of public health threats. The principal investigator (ENI) and co-principal investigator (FS) are physicians and public health researchers based in Sierra Leone with extensive experience in qualitative health research and implementation science. FS is the Executive Director and ENI is Strategic Advisor of the NPHA. The co-investigators MAV and MMS are Sierra Leonean public health professionals with direct experience in Sierra Leone’s outbreak response architecture, surveillance systems, and community-facing public health practice. MAV is a physician and senior public health leader within the NPHA, and MMS is a Principal Surveillance Officer with experience in national surveillance and outbreak response.

LN is a doctorate-trained social scientist and mental health expert with training in qualitative methods and prior experience in HIV and infectious disease research in East and West Africa. Research assistants (n = 8) were recruited from Sierra Leone; all held at minimum a bachelor’s degree in a health-related field, and four had prior experience in qualitative data collection during the Ebola response. Research assistants received five days of training covering qualitative methods, ethical considerations, sensitive interviewing, emotional support provision, and mpox-specific knowledge. The team included members with prior experience in Ebola response research, providing valuable contextual understanding [9,10].

We maintained reflexive practices throughout, recognising that researchers’ positions—as healthcare professionals, public health practitioners, and Sierra Leoneans navigating the same outbreak—shaped data collection and interpretation. Weekly team debriefings addressed emerging insights, analytic decisions, and researcher well-being. Reflexive memos documented team members’ evolving understandings, emotional responses, and methodological considerations.

### Data analysis

Analysis followed the Framework Method, enabling systematic cross-case comparison and transparent matrix-based synthesis [32,33]. Analysis proceeded through six phases:

Familiarisation: Analysts read complete transcripts multiple times, noting initial observations; (2) Developing an analytical framework: Initial coding of a subset of transcripts to develop a working codebook informed by the SEM/HSDF theoretical framework and emergent concepts; (3) Indexing: Systematic coding of all transcripts using the agreed codebook; (4) Charting: Summarising coded data into framework matrices organised by theme and participant group; (5) Cross-case analysis: Identifying patterns and variations across participants within and between stakeholder groups; (6) Interpretation: Developing higher-order interpretations connecting findings to broader literature and implications.

Because stigma formed part of the study aims and the HSDF sensitising framework, the analysis did not treat the mere presence of stigma as wholly inductive. The primary analytical question was not whether stigma existed, but what forms and mechanisms of stigma emerged and how these affected individuals, families, communities, healthcare workers, contact tracing, and system response. We therefore distinguished between framework-informed domains and data-led extensions of those domains during coding, charting, and interpretation.

A critical analytical decision concerned how to integrate and differentiate perspectives across the four participant groups. We constructed separate framework matrices for each stakeholder group before conducting cross-group analysis, ensuring that patterns within each group were identified before comparisons were drawn. In our interpretive process, we explicitly distinguished between first-person experiential accounts (primarily from survivors), professional observational accounts (primarily from healthcare workers and contact tracers), and community normative perspectives (primarily from community members). Where healthcare workers or contact tracers spoke about patient experiences, we treated these as institutionally situated observations rather than direct evidence of lived experience, and we note this distinction in our presentation of findings.

Convergence across groups was interpreted as strengthening credibility, while divergence prompted further analytical interrogation of the epistemic basis for differing accounts.

Data management was conducted using NVivo 14 (Lumivero, 2023). Coding was led by ENI and LN, both of whom had doctoral-level training in qualitative research methods. ENI brought embedded knowledge of Sierra Leone’s health system and outbreak response expertise while LN brought a comparative perspective from qualitative research experience in East African settings. A team-based approach to coding was employed, with initial independent coding by both analysts followed by consensus discussions to resolve discrepancies. An audit trail documented all analytic decisions (Table B in S5 Appendix). Subgroup analyses examined variations by gender, age group (18–24, 25–34, 35–49, 50+), district, urban/rural residence, education level, and for healthcare workers, professional role and facility type. Given the qualitative nature of the study and modest subgroup sizes, we report observed patterns as exploratory observations rather than generalisable subgroup differences, and we exercise caution in interpretation where cell sizes are small. We also attended to how social positions may intersect—for example, how gender and rural residence jointly shaped stigma experiences—rather than treating demographic categories as independent, parallel variables. Member checking was conducted with 20 participants (5 from each stakeholder group) who reviewed preliminary findings and provided feedback that was incorporated into the final analysis.

## Results

### Participant characteristics

Table 1 presents participant demographics. Among the 42 mpox survivors, 26 (61.9%) were male and 16 (38.1%) were female, with a mean age of 28.4 years (range: 18–52). The majority resided in Western Area Urban (n = 25, 59.5%), followed by Western Area Rural (n = 9, 21.4%), Bo (n = 5, 11.9%), and Kenema (n = 3, 7.1%). Time since recovery ranged from 2–14 weeks at interview. Among healthcare workers (n = 38), 22 (57.9%) were nurses, 8 (21.1%) were physicians, 5 (13.2%) were laboratory technicians, and 3 (7.9%) were IPC officers. Community members (n = 36) were distributed across all four districts with deliberate oversampling from communities with the highest case burdens.

**Table 1:** Demographic characteristics of study participants.

Characteristic	Survivors (n = 42)	Healthcare workers (n = 38)	Community (n = 36)	Contact Tracers (n = 24)
Gender – Male	26 (61.9%)	15 (39.5%)	18 (50.0%)	16 (66.7%)
Gender – Female	16 (38.1%)	23 (60.5%)	18 (50.0%)	8 (33.3%)
Age (mean, range)	28.4 (18–52)	36.2 (24–58)	38.7 (22–65)	32.1 (23–48)
Western Area Urban	25 (59.5%)	22 (57.9%)	14 (38.9%)	10 (41.7%)
Western Area Rural	9 (21.4%)	8 (21.1%)	10 (27.8%)	6 (25.0%)
Bo District	5 (11.9%)	5 (13.2%)	6 (16.7%)	4 (16.7%)
Kenema District	3 (7.1%)	3 (7.9%)	6 (16.7%)	4 (16.7%)

Contact tracers (n = 24) represented all DHMTs involved in mpox response in the four study districts.

Analysis identified five overarching themes with multiple subthemes, summarised in the thematic framework (Fig A in S1 Appendix), with the full diagram and explanation provided in S1 Appendix.

### Theme 1: “The body that betrays”—Illness experiences

Survivors described illness experiences characterised by physical suffering, diagnostic uncertainty, and lasting bodily changes. This theme encompassed four subthemes: onset and recognition, physical suffering, diagnostic journeys, and altered body experience.

#### Onset and recognition.

Initial symptom recognition was frequently delayed or misattributed, particularly in the outbreak’s early phases. Many survivors described initially interpreting symptoms through familiar illness categories—malaria, skin infections, or sexually transmitted infections:

*“I first felt the fever coming and thought, this is malaria again. I took the [antimalarial] tablets and waited. When the bumps started appearing on my skin, I thought maybe it was an allergic reaction. It was only when my friend told me about mpox being in Freetown that I started to worry it could be that.” (S-M-24- WAU).*

#### Physical suffering.

Survivors described intense physical suffering that often exceeded their expectations. Pain was a dominant feature, particularly associated with lesions, lymphadenopathy, and systemic symptoms. Survivors frequently reported limited access to effective pain relief (S6 Appendix):

*“The pain was like nothing I have experienced before. Every lesion felt like fire burning on my skin. I could not sleep, I could not eat, and I could not even lie down comfortably. The lymph nodes under my arms were so swollen that I could not lower my arms to my sides. I thought I was going to die.” (S-M-35-WAU)*

#### Diagnostic journeys.

Pathways to diagnosis were often circuitous, involving multiple healthcare encounters. Several survivors described initial misdiagnosis:

*“The first clinic I went to, they said it was chickenpox and gave me calamine lotion. After two days, I went back because it was getting worse, and they said maybe it was syphilis and gave me injections. It was only when I went to [government hospital] that they finally said it could be mpox and took the swab.” (S-M-29-WAU)*

#### Altered body experience.

Beyond acute illness, survivors described lasting changes in their relationship with their bodies, including scarring and impacts on sexuality and intimacy:

*“Since I recovered, I have not been able to be intimate with my husband. Every time I think about it, I remember the sores in that area, and I feel afraid and ashamed. He is patient with me, but I can see he is frustrated. I do not know if this feeling will ever go away.” (S-F-34-WAU)*

### Theme 2: “Marked and marginalised”—Multi-layered stigma

Distinct mechanisms through which stigma manifested emerged across all participant groups and study sites. Stigma operated at multiple levels and through four interconnected forms: enacted stigma and discrimination, perceived stigma and anticipated rejection, internalised stigma and self-blame, and associative stigma (see S6 Appendix).

#### Enacted stigma and discrimination.

Survivors reported direct experiences of discrimination from multiple sources, including family rejection, community avoidance, and healthcare discrimination:

*“When my family found out I had mpox, my own brother told me I could not stay in the house anymore. He said I would infect his children. I had to go and stay with a friend who was the only one willing to take me in.” (S-M-26-WAR)*

#### Perceived stigma and anticipated rejection.

Perceived stigma shaped behaviour, particularly around disclosure:

*“I told no one except my mother and one close friend. Not even my employer—I said I had malaria and needed rest. I was so afraid that if people knew, I would lose everything.” (S-F-27-BO)*

#### Internalised stigma and self-blame.

Many survivors described accepting negative social messages, leading to shame and self- blame, particularly when illness was attributed to sexual behaviour:

*“I kept asking myself, what did I do to deserve this? I felt so dirty, so ashamed. I thought maybe God was punishing me for something I had done. I could not even look at myself in the mirror.” (S-M-25-WAU)*

#### Associative stigma.

Stigma extended to families, caregivers, and those associated with outbreak response. Both survivors and healthcare workers described this pattern. Healthcare workers reported being avoided by neighbours and community members because of their roles:

*“My neighbours know I work at the mpox treatment centre. Some of them have started avoiding me. One woman actually told me she does not want me coming near her children because I might bring the disease to the community. It is hurtful because I am trying to help people.” (HCW-F-34-WAU-Nurse)*

### Theme 3: “Seeking care in silence”—Healthcare-seeking trajectories

Multiple factors influenced healthcare-seeking behaviours, including stigma, knowledge, economic constraints, and health system accessibility. Three subthemes characterised these trajectories: delays and barriers, alternative care pathways, and facilitators of timely care (S6 Appendix).

#### Delays and barriers.

Fear of stigma was the most frequently cited reason for delayed care-seeking:

*“I waited because I was afraid of what would happen if people found out. I thought maybe if I just stayed home, it would go away on its own, and no one would ever need to know.” (S-M-22-WAU)*

Care-seeking delays varied across social position and geography, but these observations were exploratory rather than comparative. Urban participants more often described concealment strategies shaped by fear of disclosure at work or within dense social networks. Rural participants more often described transport costs, distance to facilities, and limited diagnostic access. Participants in formal employment described fear of workplace disclosure and job loss, while those in informal work emphasised income loss from missing daily labour. Because several subgroups were small, particularly outside Western Area, these patterns are presented narratively rather than as subgroup comparisons.

#### Alternative care pathways.

Some survivors described managing symptoms at home or seeking less visible sources of care before reaching mpox-specific services. These pathways were shaped by uncertainty about early symptoms and fear that attending a treatment centre would disclose their illness to others:

“At first, I did not know what it was. I thought it could be malaria or a skin infection, so I bought medicine quietly and stayed home. I was also afraid that if I went to the mpox centre, people would see me and start talking, and no one would ever buy tomatoes from my stall again.” (S-F-30-WAU)

#### Facilitators of timely care.

Survivors who sought care promptly described factors that facilitated their decision, including awareness of mpox symptoms from public health messaging, social support, and prior positive healthcare experiences:

*“I had seen the messages on the radio about mpox and what signs to look for. When I started having those exact symptoms, I knew I should go get tested right away.” (S-M-33-WAU)*

### Theme 4: “Working at the edge”—Healthcare worker experiences

Healthcare workers described experiences of responding to the outbreak while navigating resource constraints, occupational risks, emotional burdens, and community pressures.

Four subthemes captured these experiences. For concision, two central subthemes are presented below; the full set of subthemes and extended illustrative quotations are provided in S6 Appendix.

#### Clinical challenges and moral distress.

Healthcare workers described confronting clinical situations for which they felt inadequately prepared:

*“There were times when we had more patients than beds. We had to make difficult decisions about who could be admitted and who had to go home. Some patients we sent home were very sick, but we had no space for them. I would lie awake at night wondering if I had made the right choice.” (HCW-M-38-WAU- Physician)*

#### Emotional toll and compound trauma.

The emotional burden was substantial. Many healthcare workers who had served during previous epidemics described the mpox response reactivating earlier distress:

*“When mpox started spreading, it brought back all those memories from Ebola. The fear, the death, watching colleagues get sick. I thought I had processed all of that but it came flooding back. Some nights I could not sleep.” (HCW-F-45-WAU- Nurse)*

### Theme 5: “Building the plane while flying”—Health system response

The final theme addresses the health system response, drawing primarily on accounts from healthcare workers and contact tracers. Participants described a response that was adaptive but strained, with frontline teams often adjusting referral pathways, communication practices, and community engagement strategies as the outbreak evolved. Four subthemes characterised this domain: adaptive capacity and improvisation, coordination challenges, vaccination experiences, and contact tracing barriers (S6 Appendix).

#### Adaptive capacity and improvisation.

Frontline teams described adjusting their work in real time as case numbers increased and formal systems struggled to keep pace.

*“There was no perfect system at the beginning. Every day the situation changed, so we also changed how we worked. Sometimes the official message came late, and we used phone calls, WhatsApp groups, and people we trusted in the community to move faster. It was not ideal, but if we waited for everything to be arranged, the cases would move ahead of us.” (CT-M-32-WAU)*

Beyond communication improvisation, frontline adaptation extended to treatment capacity management. Healthcare workers described facilities that reached capacity rapidly during the outbreak’s acceleration phase, requiring informal arrangements for patient transfer between centres, rotation of staff, and triage of cases for home-based isolation when admission was not possible. Referral pathways were negotiated in real time rather than through formal protocols, and transport availability—for both patients and specimens—was a recurrent constraint that affected the speed of both clinical response and surveillance (S6 Appendix).

#### Coordination challenges.

Participants described delays and fragmentation in information flow across laboratory, surveillance, treatment, and field teams.

*“Sometimes the laboratory result, the case list, and the field team were not moving at the same speed. A patient could be confirmed, but by the time the information reached us, the family had already moved or the contacts were no longer easy to find. The problem was not that people refused to work. The problem was that the information was coming in pieces.” (CT-F-29-WAR)*

Transport constraints compounded information delays. Specimen movement between collection points and laboratories depended on vehicles not always prioritised for surveillance functions. Contact tracers in rural and peri-urban areas described situations in which the window for identifying and isolating contacts had passed by the time laboratory confirmation reached field teams. These gaps were understood by participants as systemic failures of coordination rather than individual lapses, but they accumulated into measurable delays in the response chain (S6 Appendix).

#### Vaccination experiences.

Vaccination was described as protective, but also socially sensitive in communities where mpox was associated with shame, exposure, or sexual transmission.

*“People wanted protection, but they were also afraid of what others would think if they joined the vaccination line. Some said, ‘If they see me there, they will say I have been exposed.’ So the vaccine itself became connected to the same shame as the disease. We had to explain that vaccination was protection, not a confession.” (HCW-F-36-WAU-Nurse)*

Vaccine prioritisation and eligibility also created operational and social tension. Initial supplies were limited, and allocation decisions—across healthcare workers, close contacts, and community members—were experienced as inconsistent. Healthcare workers at peripheral facilities described receiving later access to vaccination than those at referral centres, which they perceived as increasing their occupational exposure during the outbreak’s early phase. Community members described uncertainty about eligibility criteria: some understood vaccination as reserved for confirmed contacts, while others were unsure whether seeking vaccination required disclosure of potential exposure. These ambiguities, compounded by the social stigma attached to vaccination in some communities, limited uptake among those who might have benefited most (S6 Appendix).

#### Contact tracing barriers.

Contact tracing was particularly difficult when naming contacts could expose sexual relationships, household conflict, workplace absence, or socially disapproved behaviour.

*“Many patients are reluctant to name their contacts, especially if the contact was through sexual activity. They are afraid of what will happen to those people, or they do not want to reveal their private behaviour.” (CT-M-34-WAU)*

Beyond sexual exposure, contact tracers described how household tensions, employment concerns, and community visibility created additional barriers. Participants in informal employment feared that being named as a contact would render their illness visible to employers or community leaders, threatening their livelihoods. In some cases, contact tracers described extending timelines informally to allow participants to disclose to family members before notification proceeded, balancing surveillance objectives against participant safety and consent (S6 Appendix).

#### Subgroup variations.

Across the findings, subgroup variation appeared suggestive but not definitive. Female survivors more frequently described internalised stigma linked to perceived sexual transmission and intimate relationships, while male survivors more often emphasised economic impacts and work disruption. Younger adults described peer-level stigma, social media disclosure fears, and economic vulnerability. Older participants more often described family-based discrimination and concerns compounded by pre-existing health conditions. Rural participants generally described longer care-seeking pathways, whereas urban participants described more elaborate concealment strategies. These observations are exploratory and should not be interpreted as generalisable subgroup differences.

## Discussion

This study provides an in-depth qualitative exploration of mpox outbreak experiences in Sierra Leone, revealing physical suffering, multi-layered stigma, healthcare-seeking challenges, healthcare worker distress, and health system response dynamics during the clade IIb epidemic. While certain themes, including disease-related stigma, healthcare-seeking delays, and healthcare worker moral distress, resonate with prior research on infectious disease outbreaks, our findings contribute empirical and conceptual insights that extend beyond contextual confirmation of known patterns [28,29].

Three contributions merit particular emphasis. First, this study provides what is, to our knowledge, the first qualitative account of mpox illness experiences from an African setting during the current PHEIC, offering empirical grounding for understanding how mpox is experienced in a context characterised by post-conflict health system fragility, sequential epidemic exposure, and cultural frameworks distinct from those documented in high-income country studies of the 2022 outbreak. Second, our multi-perspective design reveals how stigma operates not merely as a barrier at discrete points in the care pathway but as a recursive structural process that simultaneously shapes illness experience, healthcare-seeking, professional practice, contact tracing, and system-level response. Third, the phenomenon of compound trauma among healthcare workers, where mpox response reactivated unresolved distress from Ebola and COVID-19 experiences, represents a distinctive finding with implications for workforce sustainability in settings with histories of sequential health emergencies.

### Illness experiences and disrupted embodiment

Survivors’ narratives of physical suffering align with emerging clinical literature documenting severe pain as a hallmark of mpox, particularly when genital lesions are present [7,37]. Our qualitative findings add experiential depth to these clinical observations, revealing how inadequate pain management compounded suffering and eroded trust in healthcare. The phenomenon of “disrupted embodiment”—fundamentally altered relationships with one’s own body—has been documented in other illness contexts but has not previously been explored in relation to mpox [38]. Visible scarring and impacts on sexuality represent lasting consequences extending well beyond acute illness resolution. It should be noted, however, that our cross-sectional design captures experiences at a single point in recovery; longitudinal research would be needed to understand how embodied experiences evolve over time and whether the disruptions described here are enduring or gradually resolve [18].

### Stigma mechanisms and impacts

Our findings extend the HSDF to the mpox context, documenting how stigma operates through multiple mechanisms—enacted, perceived, internalised, and associative—at multiple levels from individual to institutional [15]. The visible nature of mpox lesions, coupled with perceived associations with sexual transmission, creates a particularly stigmatising configuration [16,39]. Comparisons to HIV disease were explicit in participant accounts, echoing patterns documented in the 2022 global outbreak [18,19,22].

Critically, our findings illustrate how stigma undermined outbreak control in this setting. Fear of stigma delayed care-seeking, enabling ongoing transmission. Anticipated discrimination reduced disclosure to contacts, hampering contact tracing. Internalised stigma affected mental health and recovery. These pathways indicate that stigma warrants attention not only as a social harm but as a public health concern integral to outbreak response effectiveness [12,22,40].

### Theoretical implications

Our findings have implications for the theoretical frameworks that guided the analysis. The study did not seek to establish whether stigma existed in the outbreak; rather, it examined the forms, mechanisms, pathways, and effects through which stigma shaped lived experience and response practice. This distinction matters theoretically because the empirical contribution lies in the movement of stigma across levels, not in its presence as a theme. The SEM provided a useful heuristic for organising multi-level findings, but the empirical data suggest that relationships between levels are more dynamic and recursive than the model’s nested structure implies. Stigma did not operate as a discrete factor at any single level. It functioned as a cross-cutting structural force that simultaneously shaped individual cognition, interpersonal relationships, community norms, healthcare encounters, surveillance practices, and institutional response. This finding suggests that the SEM’s conventional representation of levels as relatively independent layers may need adaptation for outbreak contexts, where a single social process can permeate and connect all levels simultaneously.

Similarly, the HSDF proved valuable for categorising stigma mechanisms, but our findings suggest areas for extension. The framework’s typology of enacted, perceived, internalised, and associative stigma captured important dimensions of participants’ accounts, yet these mechanisms operated in reinforcing cycles rather than as parallel categories. Enacted stigma fuelled perceived stigma, perceived stigma encouraged concealment, concealment delayed care-seeking and contact disclosure, and delayed disclosure intensified community suspicion and further enacted stigma. This cyclical dynamic, while implied by the framework, deserves more explicit theorisation, particularly in outbreak contexts where the temporal compression of an epidemic may accelerate stigma cycles in ways not fully captured by frameworks developed for chronic conditions such as HIV. Future research might explore how the HSDF could be adapted to account for the rapid onset, acute visibility, sexual and moral associations, and epidemic tempo of mpox-related stigma compared with the more gradual stigma trajectories characteristic of chronic infectious diseases [40].

Beyond the cyclical dynamic, several inductive extensions of the HSDF emerged from the data that were not anticipated by the framework. Associative stigma, conventionally documented toward family members and caregivers, extended in this study to contact tracers and outbreak response personnel who were stigmatised by association with the response itself rather than with illness. Institutional settings associated with mpox care—treatment centres and vaccination sites—also carried stigmatising meaning: participants avoided vaccination queues and treatment facilities to prevent public identification with the disease, a form of institutionally located stigma not captured by the framework’s original typology. Religious and moral idioms shaped internalised stigma in ways that extended beyond the framework’s conception of self-stigma; survivors drew on spiritual frameworks of punishment and moral failing to account for their illness, reflecting culturally specific self-attribution processes absent from frameworks developed primarily in secular, high-income contexts. Finally, informal stigma-mitigation practices emerged spontaneously among frontline response teams: contact tracers and healthcare workers developed strategies to reduce the visibility of response activities, adopt neutral language in community settings, and avoid publicly identifying individuals through observable behaviour. These adaptations represent a form of stigma-responsive practice grounded in lived outbreak experience and warrant recognition in response training and preparedness guidance.

### Healthcare workers’ experiences

Healthcare workers’ narratives of moral distress, occupational risk anxiety, and emotional burden align with documented experiences from other outbreaks while revealing mpox-specific dimensions [22–26]. The compound effect of sequential outbreak response was especially salient. Many participants had served during both Ebola and COVID-19 before being asked to respond to mpox, and they described earlier memories, fears, and losses returning during the new response. This pattern is not fully captured by burnout alone. Burnout offers an important comparator because it describes emotional exhaustion, depersonalisation, and reduced personal accomplishment arising through chronic occupational strain [27,41]. In this study, however, healthcare workers described the reactivation of prior outbreak-related fear, grief, and moral conflict across sequential public health emergencies. We therefore use compound trauma descriptively to refer to the cumulative emotional and professional burden produced when successive epidemic responses reactivate prior exposure before adequate recovery or institutional repair occurs.

This descriptive interpretation is anchored in, but does not attempt to develop, the wider literature on moral distress, moral injury, and cumulative disaster exposure. Moral distress describes knowing the ethically appropriate action while being constrained from acting [42], and the crescendo effect explains how moral residue can accumulate across repeated incidents [43]. Moral injury captures longer-term psychological, social, moral, and identity-related effects of witnessing, failing to prevent, or participating in events that transgress deeply held moral beliefs [44,45]. Disaster literature similarly shows that multiple or successive exposures can deepen mental health effects and delay recovery, particularly when events occur in close temporal proximity [46–48]. A living systematic review on health and care workers during COVID-19 and other public health emergencies of international concern likewise highlights mental health, well-being, and quality-of-life impacts as core workforce consequences of emergencies [49]. In Sierra Leone, the relevance of these concepts is heightened by the memory of Ebola, the COVID-19 response, recurrent resource scarcity, and repeated expectations that frontline staff absorb risk on behalf of the public [9,22]. We therefore present compound trauma as an empirical pattern requiring further dedicated conceptual and longitudinal study, not as a fully settled diagnostic or theoretical construct.

The implication is that psychosocial support for healthcare workers cannot be treated as an episodic add-on during each outbreak. Settings with recurrent epidemic exposure require continuity of workforce well-being programmes, access to confidential mental health support, peer support systems, supportive supervision, and institutional recognition of moral distress. We note, however, that healthcare worker accounts in this study primarily reflect the perspectives of those who remained in their posts during the outbreak; the experiences of workers who left clinical practice or were unable to continue responding remain undocumented and may differ substantially.

### Implications for practice and policy

Our findings suggest several implications for mpox response in Sierra Leone and potentially in similar settings, though we note that these are indicative rather than prescriptive, and their applicability will depend on local context and implementation considerations.

First, the pervasive role of stigma across all domains of the outbreak experience suggests that stigma-informed strategies merit integration into outbreak management frameworks from the outset, rather than being treated as secondary concerns [22,40]. This could include attention to language used in public communications, protection of patient confidentiality, community engagement to address misinformation and fear, and support for affected individuals facing discrimination [50]. The specific forms that such strategies might take require further development and evaluation.

Second, healthcare worker accounts suggest that support mechanisms, including adequate PPE, access to vaccination, psychosocial support, strategies to address associative stigma, and career protections for outbreak responders, should be established as integral components of the response architecture rather than as afterthoughts [25,26]. The compound trauma observed among workers with prior epidemic experience further suggests the potential value of continuity in workforce wellbeing programmes across successive outbreaks.

Third, the care-seeking patterns observed in this study suggest that community-centred communication approaches—emphasising symptom recognition and care availability, delivered through trusted community channels—may facilitate timely care-seeking [40,50]. However, our data captures what participants reported about their care-seeking experiences and decisions, and social desirability considerations may have shaped these accounts. Intervention research is needed to evaluate the effectiveness of specific communication strategies.

### Limitations

Several limitations should be acknowledged. First, although the sample was purposively diverse across participant groups and districts, it may not represent all experiences of the outbreak. In particular, people who never accessed healthcare, those who declined participation because of stigma, and those who experienced severe outcomes may be underrepresented. The geographic concentration of cases in Western Area may also limit transferability to districts with different epidemiological and social profiles.

Second, the study relied on retrospective self-reported accounts collected during a highly sensitive outbreak. Recall bias may have affected illness narratives, and social desirability bias may have shaped responses across participant groups. Survivors may have understated socially sensitive exposures or behaviours, community members may have moderated stigmatising attitudes, and healthcare workers may have emphasised operational constraints. Sexual exposures and partner-related experiences were also likely underreported because of stigma, fear of disclosure, and the social risks attached to discussing sexual behaviour in this setting. We therefore make no claims about individual transmission routes.

Third, the study aims and HSDF-informed sensitising framework directed analytical attention toward stigma-related experience. This may have increased attention to stigma compared with other possible dimensions of the outbreak experience. To reduce this risk, we treated the framework as a sensitising tool rather than a rigid coding template, and the specific forms, mechanisms, pathways, and effects of stigma reported here were developed from participant accounts rather than imposed in advance.

Fourth, although individual interviews were used for sensitive survivor accounts and for healthcare workers’ more personal experiences, some healthcare worker FGDs may have been affected by hierarchy and workplace dynamics. Facilitators attempted to mitigate this by monitoring group interaction, inviting dissenting views, and offering follow-up SSIs where needed, but some participants may still have moderated criticism of colleagues, supervisors, or institutional practices.

Fifth, the cross-sectional nature of data collection means that the study captures experiences at particular points in the outbreak trajectory and recovery process. How stigma, embodiment, care-seeking, and healthcare worker distress evolve over time remains an important question for longitudinal research. The absence of male partner perspectives, additional family members, and individuals who declined healthcare also limits the completeness of our account of the relational dimensions of mpox experience.

Despite these limitations, the multi-perspective design, diverse sample, member checking, framework-based analysis, and triangulation across survivor, healthcare worker, community, and contact tracer accounts support the credibility of the findings within the scope of the study.

## Conclusions

Stigma was the dominant social force shaping the mpox outbreak experience in Sierra Leone. It delayed care-seeking, undermined contact tracing, eroded trust in health services, and compounded the emotional burden on healthcare workers, all of which directly compromised outbreak control. Because stigma was anticipated in the analytical framework, the key finding is not simply that stigma existed, but that it operated recursively across individual, interpersonal, community, institutional, and system levels.

Healthcare workers with prior Ebola and COVID-19 experience described a compound trauma pattern that current support systems did not fully address. This should not be read as a settled diagnostic construct; rather, it signals that prior outbreak exposure, unresolved moral distress, and recurrent expectations of risk need to be considered in workforce support planning. Health worker support systems should therefore extend across successive outbreaks rather than being rebuilt for each emergency.

Three priorities emerge for outbreak preparedness in similar post-conflict, resource-constrained settings: (1) stigma-informed strategies integrated from the outset of outbreak response, not appended later; (2) healthcare worker support systems that extend across successive outbreaks rather than being rebuilt each time; and (3) community-centred communication, vaccination, and contact tracing approaches that protect confidentiality and reach people before stigma drives them into silence. These priorities require further evaluation, but the experiences documented here show that technical response alone is insufficient without attention to the social and workforce dimensions of epidemic disease.

## Supporting information

S1 Appendix Framework description and thematic framework summary. Integrated SEM+HSDF analytical framework, Fig A thematic framework diagram, and thematic framework overview.
(DOCX)

S2 Appendix COREQ checklist. Completed Consolidated Criteria for Reporting Qualitative Research checklist.
(DOCX)

S3 Appendix Semi-structured interview guides. Interview guides for survivors, healthcare workers, community members, and contact tracers.
(DOCX)

S4 Appendix Focus group discussion guide. Guide for focus group discussions with healthcare workers, community members, and contact tracers.
(DOCX)

S5 Appendix Coding framework. Full codebook with code names, definitions, inclusion/exclusion criteria, and example excerpts.
(DOCX)

S6 Appendix Extended participant quotations by theme. Additional quotations supporting each theme and subtheme.
(DOCX)

S1 Checklist Inclusivity in Global Research Questionnaire. (DOCX)
