# Supplementary material for: Suffering in silence: Stigma, healthcare barriers, and resilience during Sierra Leone’s 2025 clade IIb mpox outbreak—A multi-perspective qualitative study
Source: PLOS Glob Public Health. 2026 Jun 30;6(6):e0006686. doi: 10.1371/journal.pgph.0006686 (PMC13318003; doi:10.1371/journal.pgph.0006686)
Supplement: S1 Appendix — Integrated SEM+HSDF analytical framework, Fig A thematic framework diagram, and thematic framework overview. (DOCX) [file pgph.0006686.s001.docx]

**Supplementary Materials**

*Suffering in silence: Stigma, healthcare barriers, and resilience during Sierra Leone's 2025 clade IIb mpox outbreak—A multi-perspective qualitative study*

**S1 Appendix. Framework description and thematic framework summary**


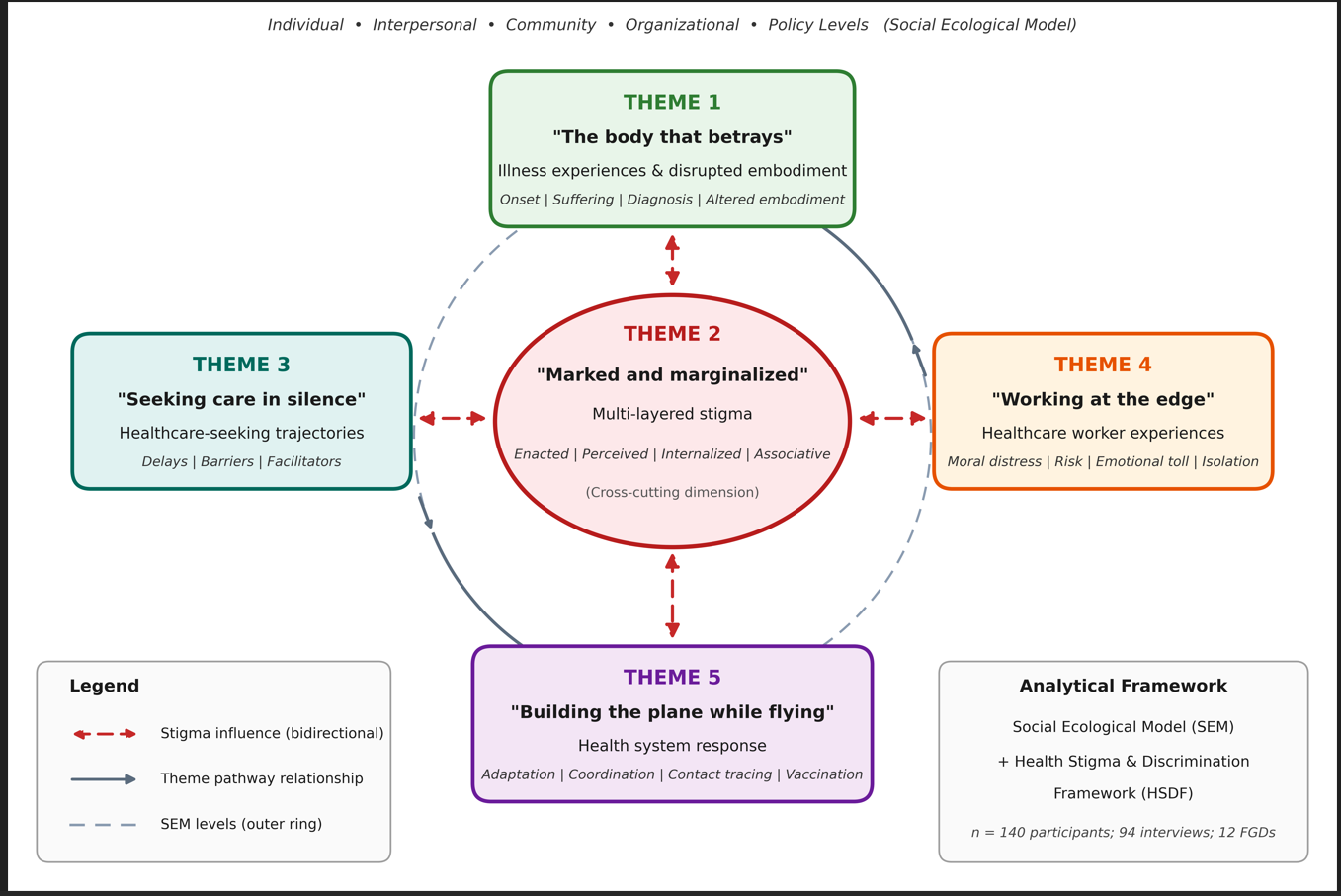


**Fig A. Thematic framework diagram showing five overarching themes and their interconnections, with Stigma (Theme 2) as a cross-cutting dimension.**

**Framework overview**

Our analytical framework combined two complementary theoretical perspectives: the Social Ecological Model (SEM) [1] and the Health Stigma and Discrimination Framework (HSDF) [2]. The SEM provides a multi-level structure for examining factors at individual, interpersonal, community, organizational, and policy levels. The HSDF offers a cross-cutting lens for understanding how stigma operates through drivers (such as fear, stereotypes, and power imbalances) and facilitators (social norms, policies, and structures).

**Application to mpox context**

In the context of the mpox outbreak, we applied this integrated framework to examine: (1) Individual level: Personal experiences of illness, coping strategies, and internalized stigma; (2) Interpersonal level: Family and peer responses, disclosure decisions, and support networks; (3) Community level: Social norms, community perceptions, enacted stigma, and collective responses; (4) Organizational level: Healthcare system responses, institutional stigma, and workplace dynamics; (5) Policy level: Public health policies, outbreak response frameworks, and structural barriers.

Stigma was examined as a cross-cutting dimension operating at and across all levels, consistent with the HSDF conceptualization. The framework guided both data collection (informing interview guide development) and analysis (providing sensitizing concepts for coding and interpretation).

**Thematic framework derived from data**

Analysis identified five overarching themes with multiple subthemes (Fig 1 above):

Theme 1: "The body that betrays"—Illness experiences and disrupted embodiment

- Onset and recognition

- Physical suffering

- Diagnostic journeys

- Altered embodiment

Theme 2: "Marked and marginalized"—Multi-layered stigma (cross-cutting)

- Enacted stigma and discrimination

- Perceived stigma and anticipated rejection

- Internalized stigma and self-blame

- Associative stigma

Theme 3: "Seeking care in silence"—Healthcare-seeking trajectories

- Delays and barriers

- Alternative care pathways

- Facilitators of timely care

Theme 4: "Working at the edge"—Healthcare worker experiences

- Clinical challenges and moral distress

- Occupational risk and protection

- Emotional toll and compound trauma

- Professional identity and isolation

Theme 5: "Building the plane while flying"—Health system response

- Adaptive capacity and innovation

- Coordination challenges

- Contact tracing barriers

- Vaccination experiences

**References for S1 Appendix**

1. McLeroy KR, Bibeau D, Steckler A, Glanz K. An ecological perspective on health promotion programs. Health Education Quarterly. 1988;15(4):351–377. doi:10.1177/109019818801500401.

2. Stangl AL, Earnshaw VA, Logie CH, van Brakel W, Simbayi LC, Barré I, Dovidio JF. The Health Stigma and Discrimination Framework: a global, crosscutting framework to inform research, intervention development, and policy on health-related stigmas. BMC Medicine. 2019;17:31. doi:10.1186/s12916-019-1271-3.
