# Supplementary material for: Suffering in silence: Stigma, healthcare barriers, and resilience during Sierra Leone’s 2025 clade IIb mpox outbreak—A multi-perspective qualitative study
Source: PLOS Glob Public Health. 2026 Jun 30;6(6):e0006686. doi: 10.1371/journal.pgph.0006686 (PMC13318003; doi:10.1371/journal.pgph.0006686)
Supplement: S2 Appendix — Completed Consolidated Criteria for Reporting Qualitative Research checklist. (DOCX) [file pgph.0006686.s002.docx]

**Supplementary Materials**

*Suffering in silence: Stigma, healthcare barriers, and resilience during Sierra Leone's 2025 clade IIb mpox outbreak—A multi-perspective qualitative study*

**S2 Appendix. COREQ checklist**

Consolidated Criteria for Reporting Qualitative Research (COREQ): 32-item checklist

***Note:*** *We report locations using manuscript section labels. Final page numbers will be updated after typesetting to match the final formatted submission.*

**Table A. COREQ checklist.**

| **Item No.** | **Guide question/description** | **Reported on page** |
| --- | --- | --- |
| Domain 1: Research team and reflexivity |  |  |
| 1 | Interviewer/facilitator: Which author(s) conducted the interviews or focus groups? | Methods: Data collection |
| 2 | Credentials: What were the researcher's credentials? | Methods: Research team |
| 3 | Occupation: What was their occupation at the time of the study? | Methods: Research team |
| 4 | Gender: Was the researcher male or female? | Methods: Research team |
| 5 | Experience and training: What experience or training did the researcher have? | Methods: Research team |
| 6 | Relationship established: Was a relationship established prior to study commencement? | Methods: Recruitment |
| 7 | Participant knowledge of the interviewer: What did the participants know about the researcher? | Methods: Informed consent |
| 8 | Interviewer characteristics: What characteristics were reported about the interviewer/facilitator? | Methods: Research team |
| Domain 2: Study design |  |  |
| 9 | Methodological orientation and theory: What methodological orientation was stated? | Methods: Study design |
| 10 | Sampling: How were participants selected? | Methods: Participants and sampling |
| 11 | Method of approach: How were participants approached? | Methods: Recruitment |
| 12 | Sample size: How many participants were in the study? | Results: Participant characteristics |
| 13 | Non-participation: How many people refused to participate or dropped out? | Methods: Recruitment |
| 14 | Setting of data collection: Where was the data collected? | Methods: Data collection |
| 15 | Presence of non-participants: Was anyone else present besides the participants and researchers? | Methods: Data collection |
| 16 | Description of sample: What are the important characteristics of the sample? | Results: Table 1 |
| Domain 3: Analysis and findings |  |  |
| 17 | Data collection: Were questions, prompts, guides provided by the authors? | S3 Appendix and S4 Appendix |
| 18 | Audio/visual recording: Was the interview audio/video recorded? | Methods: Data collection |
| 19 | Field notes: Were field notes made during and/or after the interview? | Methods: Research team |
| 20 | Duration: What was the duration of the interviews or focus groups? | Methods: Data collection |
| 21 | Data saturation: Was data saturation discussed? | Methods: Sampling |
| 22 | Transcripts returned: Were transcripts returned to participants for comment? | Methods: Data analysis |
| 23 | Number of data coders: How many data coders coded the data? | Methods: Data analysis |
| 24 | Description of the coding tree: Did authors provide a description of the coding tree? | S5 Appendix |
| 25 | Derivation of themes: Were themes identified in advance or derived from the data? | Methods: Data analysis |
| 26 | Software: What software, if applicable, was used to manage the data? | Methods: Data analysis |
| 27 | Participant checking: Did participants provide feedback on the findings? | Methods: Data analysis |
| 28 | Quotations presented: Were participant quotations presented to illustrate themes? | Results |
| 29 | Data and findings consistent: Was there consistency between the data presented and the findings? | Results |
| 30 | Clarity of major themes: Were major themes clearly presented in the findings? | Results |
| 31 | Clarity of minor themes: Is there a description of diverse cases or discussion of minor themes? | Results |
