# Supplementary material for: Suffering in silence: Stigma, healthcare barriers, and resilience during Sierra Leone’s 2025 clade IIb mpox outbreak—A multi-perspective qualitative study
Source: PLOS Glob Public Health. 2026 Jun 30;6(6):e0006686. doi: 10.1371/journal.pgph.0006686 (PMC13318003; doi:10.1371/journal.pgph.0006686)
Supplement: S3 Appendix — Interview guides for survivors, healthcare workers, community members, and contact tracers. (DOCX) [file pgph.0006686.s003.docx]

**Supplementary Materials**

*Suffering in silence: Stigma, healthcare barriers, and resilience during Sierra Leone's 2025 clade IIb mpox outbreak—A multi-perspective qualitative study*

**S3 Appendix. Semi-structured interview guides**

**Semi-structured interview guide for mpox survivors**

**Introduction and rapport building**

Thank you for agreeing to speak with me today. I am a trained member of the research team from the National Public Health Agency. We conduct this study to understand the experiences of people who experienced mpox in Sierra Leone. Your participation is voluntary. You can stop at any time or skip any question. We keep everything you share confidential and remove personal identifiers from transcripts. Do you have any questions before we begin?

**Section 1: Illness onset and trajectory**

1. Can you tell me about when you first started feeling unwell? What symptoms did you notice first?

2. What did you think was happening to you at that time?

3. Can you describe how your illness progressed over time?

4. What was the most difficult part of being ill with mpox?

Probes: Physical symptoms, pain, limitations on activities, emotional aspects

**Section 2: Healthcare-seeking experiences**

5. Can you walk me through what you did when you realized you might need medical care?

6. What factors influenced your decision about when and where to seek care?

7. Can you describe your experiences at the healthcare facility?

8. How did healthcare workers treat you?

Probes: Wait times, privacy, communication, respect, quality of care

**Section 3: Social responses and stigma**

9. Who did you tell about your diagnosis? How did you decide who to tell?

10. How did your family react when they learned about your illness?

11. How did people in your community respond?

12. Did you experience any negative treatment because of having mpox? If so, can you describe what happened?

Probes: Rejection, avoidance, discrimination, blame, isolation

**Section 4: Coping and support**

13. What helped you cope during your illness and recovery?

14. Who supported you the most during this time?

15. What additional support would have been helpful?

**Section 5: Recovery and ongoing impacts**

16. How are you feeling now that you have recovered?

17. Are there any lasting effects from your illness—physical, emotional, or social?

18. How has this experience changed you or your life?

**Section 6: Recommendations**

19. Based on your experience, what advice would you give to someone who thinks they might have mpox?

20. What could be done to improve the experience for people with mpox in Sierra Leone?

21. Is there anything else you would like to share about your experience?

**Semi-structured interview guide for healthcare workers**

**Introduction**

Thank you for agreeing to participate in this study. We are interested in understanding the experiences of healthcare workers involved in the mpox response in Sierra Leone. Your responses will be kept confidential.

**Section 1: Background and role**

1. Can you tell me about your role in the mpox response?

2. How did you become involved in mpox care/response?

3. What training did you receive for working with mpox patients?

**Section 2: Clinical experiences**

4. Can you describe what it has been like caring for mpox patients?

5. What have been the most challenging aspects of providing care?

6. Have there been situations where you felt unable to provide the care you wanted to? If so, can you describe these?

Probes: Resource constraints, knowledge gaps, ethical dilemmas

**Section 3: Occupational concerns**

7. What concerns, if any, do you have about your own health while working with mpox patients?

8. Do you feel adequately protected? Why or why not?

9. Have you been vaccinated against mpox? Can you tell me about that experience?

**Section 4: Emotional and social impacts**

10. How has working in the mpox response affected you emotionally?

11. How have your family and friends responded to your work in mpox care?

12. Have you experienced any negative treatment from others because of your work?

**Section 5: Observations about patients and community**

13. From your observations, how are mpox patients treated by their families and communities?

14. What barriers do you observe affecting patients' access to care?

15. How do patients seem to cope with their illness and any stigma they experience?

**Section 6: Recommendations**

16. What additional resources or support would help you in your work?

17. What recommendations do you have for improving the mpox response?

18. Is there anything else you would like to share?
