# Supplementary material for: Suffering in silence: Stigma, healthcare barriers, and resilience during Sierra Leone’s 2025 clade IIb mpox outbreak—A multi-perspective qualitative study
Source: PLOS Glob Public Health. 2026 Jun 30;6(6):e0006686. doi: 10.1371/journal.pgph.0006686 (PMC13318003; doi:10.1371/journal.pgph.0006686)
Supplement: S4 Appendix — Guide for focus group discussions with healthcare workers, community members, and contact tracers. (DOCX) [file pgph.0006686.s004.docx]

**Supplementary Materials**

*Suffering in silence: Stigma, healthcare barriers, and resilience during Sierra Leone's 2025 clade IIb mpox outbreak—A multi-perspective qualitative study*

**S4 Appendix. Focus group discussion guide**

Focus group discussions (FGDs) used a semi-structured guide aligned to the interview topic areas, adapted for each stakeholder group (healthcare workers, community members, contact tracers). Each FGD included 6–10 participants and lasted 60–90 minutes.

**Opening and ground rules (all groups)**

1. Please introduce yourself (first name or pseudonym) and your role in the community/response.

2. We discuss experiences of mpox in Sierra Leone. You can decline any question. Please respect confidentiality and do not share other participants' comments outside this group.

**Core questions (all groups)**

3. What did people in this community/workplace understand about mpox during the outbreak?

4. What fears or concerns did you see, and what drove them?

5. What forms of stigma or discrimination did you observe (in families, communities, workplaces, facilities)?

6. How did stigma affect disclosure, care-seeking, and contact tracing?

7. What helped people seek care or support others (facilitators, trusted messengers, services)?

8. What barriers to care or response efforts mattered most (costs, transport, information, privacy, facility capacity)?

9. What strategies reduced stigma or improved care and response coordination?

10. What practical recommendations do you propose for the next outbreak phase?

**Group-specific probes**

Healthcare workers: IPC constraints, workload, moral distress, training, vaccine access, patient communication, privacy.

Community members: rumors/media, social norms, gender/age dynamics, community leadership, support networks.

Contact tracers: disclosure barriers, safety concerns, trust, data privacy, household dynamics, follow-up challenges.

**Closing (all groups)**

11. What single change would most improve response and reduce stigma?

12. Is there anything we did not ask that matters?
