# Supplementary material for: Suffering in silence: Stigma, healthcare barriers, and resilience during Sierra Leone’s 2025 clade IIb mpox outbreak—A multi-perspective qualitative study
Source: PLOS Glob Public Health. 2026 Jun 30;6(6):e0006686. doi: 10.1371/journal.pgph.0006686 (PMC13318003; doi:10.1371/journal.pgph.0006686)
Supplement: S5 Appendix — Full codebook with code names, definitions, inclusion/exclusion criteria, and example excerpts. (DOCX) [file pgph.0006686.s005.docx]

**Supplementary Materials**

*Suffering in silence: Stigma, healthcare barriers, and resilience during Sierra Leone's 2025 clade IIb mpox outbreak—A multi-perspective qualitative study*

**S5 Appendix. Coding framework**

This appendix provides the final analytic framework (themes and subthemes) generated through the Framework Method. The full codebook with code names, definitions, inclusion/exclusion criteria, and example excerpts is provided below to support transparency and reproducibility.

**Table B. Full coding framework with definitions, inclusion/exclusion criteria, and example excerpts.**

| **Theme** | **Code** | **Definition** | **Inclusion criteria** | **Exclusion criteria** | **Example excerpt** |
| --- | --- | --- | --- | --- | --- |
| Theme 1: Illness experiences | Symptom onset | Initial symptoms and recognition of illness | First symptoms noticed; early illness recognition; prodromal symptoms | Symptoms during treatment or recovery phase | "I first felt the fever coming..." |
| Theme 1: Illness experiences | Physical suffering | Pain, discomfort, functional limitations during illness | Pain descriptions; physical limitations; symptom severity | Emotional or social suffering (coded separately) | "The pain was like nothing I have experienced before..." |
| Theme 1: Illness experiences | Diagnostic journey | Path to diagnosis, healthcare encounters before confirmation | Healthcare visits before diagnosis; misdiagnosis; testing process | Treatment after confirmed diagnosis | "The first clinic I went to, they said it was chickenpox..." |
| Theme 1: Illness experiences | Altered embodiment | Changes in body relationship, scarring, intimacy impacts | Body image changes; scarring concerns; intimacy/sexuality impacts | General illness symptoms | "Since I recovered, I have not been able to be intimate..." |
| Theme 2: Stigma | Enacted stigma | Direct discrimination experiences from others | Actual rejection, avoidance, or discrimination experienced | Fear of stigma without actual experience (perceived) | "My own brother told me I could not stay in the house..." |
| Theme 2: Stigma | Perceived stigma | Anticipation or expectation of negative treatment | Fear of discrimination; anticipated rejection; concealment due to expected stigma | Actual discrimination experienced (enacted) | "I was so afraid that if people knew, I would lose everything..." |
| Theme 2: Stigma | Internalized stigma | Self-blame, shame, acceptance of negative attitudes | Self-directed negative feelings; shame; self-blame; feeling deserving of stigma | Stigma from others | "I kept asking myself, what did I do to deserve this?" |
| Theme 2: Stigma | Associative stigma | Stigma toward family, caregivers, HCWs connected to affected persons | Stigma experienced by family members, HCWs, or others associated with patients | Direct stigma toward patients themselves | "Some of them have started avoiding me..." |
| Theme 3: Healthcare-seeking | Delays | Factors delaying care-seeking | Time between symptom onset and seeking care; reasons for delayed care | Barriers during care (access barriers) | "I waited because I was afraid of what would happen..." |
| Theme 3: Healthcare-seeking | Barriers | Obstacles to accessing care once decision made | Cost barriers; transport barriers; facility capacity; geographic access | Delays before deciding to seek care | "The distance to the hospital, the transport costs..." |
| Theme 3: Healthcare-seeking | Facilitators | Factors enabling timely care | Factors promoting early care-seeking; enablers; support systems | Barriers or delays | "I had seen the messages on the radio..." |
| Theme 4: HCW experiences | Moral distress | Ethical dilemmas, resource constraints affecting care provision | Unable to provide desired care; ethical conflicts; resource limitation impacts | General work challenges without ethical dimension | "We had to make difficult decisions about who could be admitted..." |
| Theme 4: HCW experiences | Emotional toll | Burnout, trauma, psychological impact of outbreak work | Emotional exhaustion; trauma responses; mental health impacts | Physical health concerns (occupational risk) | "It brought back all those memories from Ebola..." |
| Theme 4: HCW experiences | Occupational risk | Concerns about infection, protection adequacy | Infection fears; PPE concerns; vaccination access; workplace safety | Emotional/psychological impacts (emotional toll) | "I worry about bringing it home to my family..." |
| Theme 5: Health system | Adaptive capacity | Innovation, flexibility in response | New protocols; creative solutions; system adaptations; innovations | Challenges or failures | "We had to develop new protocols quickly..." |
| Theme 5: Health system | Coordination challenges | Gaps in multi-sector coordination | Communication failures; conflicting guidelines; inter-agency coordination problems | Individual-level challenges | "Sometimes the left hand doesn't know what the right is doing..." |
| Theme 5: Health system | Contact tracing barriers | Challenges in case investigation and contact follow-up | Contact disclosure reluctance; tracing difficulties; community resistance | General healthcare access barriers | "Many patients are reluctant to name their contacts..." |
