# Supplementary material for: Suffering in silence: Stigma, healthcare barriers, and resilience during Sierra Leone’s 2025 clade IIb mpox outbreak—A multi-perspective qualitative study
Source: PLOS Glob Public Health. 2026 Jun 30;6(6):e0006686. doi: 10.1371/journal.pgph.0006686 (PMC13318003; doi:10.1371/journal.pgph.0006686)
Supplement: S6 Appendix — Additional quotations supporting each theme and subtheme. (DOCX) [file pgph.0006686.s006.docx]

**Supplementary Materials**

*Suffering in silence: Stigma, healthcare barriers, and resilience during Sierra Leone's 2025 clade IIb mpox outbreak—A multi-perspective qualitative study*

**S6 Appendix. Extended participant quotations by theme**

This appendix provides additional quotations supporting each theme and subtheme identified in the analysis.

**Theme 1: "The body that betrays"—Illness experiences**

**Physical suffering**

"I could not even wear clothes because any fabric touching my skin was agony. I had to stay naked in my room, covered only with a sheet that I held away from my body. The lesions in my private areas were the worst—I could not urinate without crying from the pain." (S-F-29-WAU)

"At night the pain would wake me every hour. I tried everything—paracetamol, ibuprofen, even some traditional medicine my grandmother brought. Nothing really helped. The doctors at the center gave me what they had, but they admitted there wasn't enough pain medicine." (S-M-31-WAR)

**Diagnostic journeys**

"I went to three different places before anyone mentioned mpox. The private clinic said it was a skin allergy. The pharmacy gave me cream for fungal infection. It was only when I went to the government hospital and they saw how many lesions I had that they took it seriously." (S-F-24-BO)

**Theme 2: "Marked and marginalized"—Multi-layered stigma**

**Enacted stigma and discrimination**

"When I returned home after discharge, I found my landlord had put my belongings outside. He said he did not want 'that disease' in his property. I had nowhere to go with my children. My sister finally took us in, but even she kept us in a separate room for the first week." (S-F-38-WAU)

"At the health center where I used to go for my other health needs, after they found out I had mpox, the attitude changed completely. The nurses would put on extra gloves just to talk to me. They made me wait until all other patients were seen. It was humiliating." (S-M-42-KEN)

**Perceived stigma and anticipated rejection**

"I created an elaborate story about having severe malaria that required hospitalization. I told this to everyone—my employer, my neighbours, even some family members. I was too afraid of what would happen if the truth came out. Even now, months later, I have not told anyone the real diagnosis." (S-M-28-WAU)

**Internalized stigma and self-blame**

"I looked at myself in the mirror and all I could see was something dirty, something contaminated. I scrubbed my skin raw trying to feel clean again. I know now this thinking was not healthy, but at the time I truly believed I deserved what happened to me." (S-F-26-WAU)

**Theme 3: "Seeking care in silence"—Healthcare-seeking trajectories**

**Economic barriers**

"I sell vegetables in the market. If I go to the hospital and they keep me there, who will sell? Who will feed my children? I waited as long as I could, trying to manage at home, because I could not afford to stop working. By the time I went, I was very sick." (S-F-45-WAR)

**Geographic access**

"In our village, there is only a small health post. The nurse there had never seen mpox before. She told me I needed to go to Bo, but that is three hours by okada [motorcycle taxi] and costs SLE 150,000. For many people here, that is impossible." (S-M-35-BO)

**Theme 4: "Working at the edge"—Healthcare worker experiences**

**Clinical challenges and moral distress**

"We had a young woman, maybe 25 years old, in terrible pain. She was crying, begging us to help her. We had given her all the paracetamol we could, but she needed stronger pain relief. We had none. I went home that night and I cried myself because I could not help her." (HCW-F-32-WAU-Nurse)

"When the numbers were at their highest, we were admitting 15-20 new patients per day. We simply did not have enough beds, enough staff, enough supplies. I had to decide who got a bed and who had to wait. Those decisions haunt me still." (HCW-M-44-WAU-Physician)

**Personal protective concerns**

"I was not vaccinated until three months into working with mpox patients. Every day I wondered if today would be the day I got infected. I would check my body constantly for any spots. The anxiety was exhausting." (HCW-F-28-WAU-Nurse)

**Theme 5: "Building the plane while flying"—Health system response**

**Contact tracing barriers**

"I had one case where the patient named 15 contacts initially. When I followed up, I could only reach 4 of them. The rest either gave false phone numbers or refused to speak with me. Without cooperation, contact tracing becomes almost impossible." (CT-M-29-WAU)

"Some contacts become hostile when we visit. They say 'Why are you coming here, do you want the neighbors to think we have mpox?' They slam doors in our faces. I understand their fear, but it makes our job very difficult." (CT-F-34-WAR)

**Coordination challenges**

"There were times when the national level was saying one thing and the district was doing something different. The guidelines kept changing. This week we do this, next week we do that. It was confusing for us and for patients." (HCW-M-48-BO-IPC Officer)
