# Supplementary material for: Suffering in silence: Stigma, healthcare barriers, and resilience during Sierra Leone’s 2025 clade IIb mpox outbreak—A multi-perspective qualitative study
Source: PLOS Glob Public Health. 2026 Jun 30;6(6):e0006686. doi: 10.1371/journal.pgph.0006686 (PMC13318003; doi:10.1371/journal.pgph.0006686)
Supplement: S1 Checklist — (DOCX) [file pgph.0006686.s007.docx]

**Inclusivity in Global Research Questionnaire**

**Completed questionnaire for PLOS Global Public Health Manuscript ID: PGPH-D-26-00336 Suffering in silence: Stigma, healthcare barriers, and resilience during Sierra Leone's 2025 clade IIb mpox outbreak—A multi-perspective qualitative study**

**Author note:** This study was conceived, approved, led, analysed, and interpreted from within Sierra Leone’s national public health system. The research team comprised Sierra Leonean public health professionals and leaders at every level of the work. Specialist qualitative and mental health expertise was contributed by a PhD-trained co-investigator with prior experience in HIV and infectious disease research in East and West Africa. Page references correspond to the revised manuscript submitted with this revision.

# Ethical considerations, permits and authorship

This section is applicable to all research types.

**1. Provide details as to who granted permissions and/or consent for the study to take place in the Methods section of your manuscript. This should include the names of all ethics boards, governmental organizations, community leaders or other bodies that provided approval for the study. If individuals provided approval, refer to these people by role or title but do not list their names. Reported on page number:**

| **Response:** Ethical approval was granted by the Sierra Leone Ethics and Scientific Review Committee (SLESRC-2025-049) on 28 February 2025. The study was implemented under the institutional leadership of the National Public Health Agency (NPHA), Sierra Leone's national institution responsible for public health protection, disease surveillance, outbreak preparedness and response, and evidence generation to understand drivers of public health threats. Engagement was also conducted through District Health Management Teams, participating health facilities, treatment centres, surveillance teams, and community health structures. Community leaders and community health workers supported community entry but did not provide consent on behalf of individual participants. All participants provided individual written informed consent before data collection; for participants who could not write, witnessed thumbprint consent was obtained in accordance with SLESRC procedures. Reported on: Methods, Ethics Statement section, page 8. |
| --- |

**2. If there were any deviations from the study protocol after approval was obtained, please provide details of these changes in the Methods section of your manuscript.**

| **Response:** No deviations from the approved study protocol occurred. Operational adaptations—such as scheduling interviews at locations chosen by participants and conducting interviews in participant-preferred languages—were consistent with the approved procedures and designed to protect confidentiality and participant comfort. |
| --- |

**3. Did this study involve local collaborators who are residents of the country where the research was conducted or members of the community studied? If you do not have any authors from said communities, please provide an explanation below. Reported on page number:**

| **Response:** Yes. The study was conceived, approved, led, implemented, analysed, and interpreted from within Sierra Leone’s public health system and under the institutional mandate of the National Public Health Agency (NPHA). The author team comprises Sierra Leonean public health professionals who led and delivered every phase of the research. Professor Foday Sahr is a Sierra Leonean senior research professor and Executive Director of the NPHA. Dr Eric Nzirakaindi Ikoona, the corresponding and first author, is a physician-researcher based in Freetown whose public health practice, outbreak response work, and research leadership are embedded within Sierra Leone’s national public health system. Dr Mohamed Alex Vandi is a Sierra Leonean physician and Deputy Executive Director of the NPHA. Mary Magdalene Sinnah is a Sierra Leonean Principal Surveillance Officer with direct experience in national surveillance and outbreak response. Together, these authors brought institutional knowledge of Sierra Leone’s outbreak response structures, surveillance systems, and community-facing public health practice.  Lucy Namulemo was included for her specialist expertise as a PhD-trained researcher in qualitative methods and mental health, with prior experience in HIV and infectious disease research in East and West Africa. Her contribution strengthened the methodological and psychosocial dimensions of the analysis without displacing Sierra Leonean research leadership. All eight research assistants were Sierra Leoneans, fluent in the local languages used for the study—Krio, Mende, and Temne—and embedded in Sierra Leonean cultural contexts. All listed authors meet PLOS authorship criteria.  Reported on: Methods, Research team and reflexivity / Inclusivity in global research sections, pages 16–17. |
| --- |

# Human subjects research

Applicable to health research, medical research, and cross-cultural research involving human participants.

**4. Did you obtain written informed consent from a representative of the local community or region before the research took place? How did you establish who speaks for the community? Details of written informed consent obtained from study participants should be reported separately in the Methods section of your manuscript.**

| **Response:** Formal study approval was obtained through the Sierra Leone Ethics and Scientific Review Committee, and the study was implemented through Sierra Leone’s established public health emergency response structures. Community leaders and community health workers facilitated community entry and helped identify appropriate engagement pathways, but no individual was designated as speaking on behalf of a community for consent purposes. The study did not apply a community spokesperson model; individual informed consent was the operative standard for all participation decisions. This approach preserves individual autonomy and avoids conflating gatekeeper access with participant-level consent. |
| --- |

**5. How did members of the local community provide input on the aims of the research investigation, its methodology, and its anticipated outcome(s)?**

| **Response:** The study arose from the practical and ethical imperatives of Sierra Leone’s 2025 mpox outbreak response. Research aims—including the focus on stigma, healthcare-seeking barriers, healthcare worker experiences, and health system response—were shaped by priorities identified through the NPHA’s operational outbreak response and direct engagement with District Health Management Teams, facility staff, surveillance personnel, and community health workers. Interview and focus group guides were piloted with members of each relevant stakeholder group and revised before full data collection, incorporating feedback on question clarity, cultural appropriateness, and sensitivity to local stigma dynamics. Weekly team debriefings with Sierra Leonean research assistants allowed ongoing refinement of interviewing approaches based on emerging field experience. Member checking with participants from all four stakeholder groups ensured that preliminary interpretations reflected participants’ intended meanings. |
| --- |

**6. When engaging with the local community, how did you ensure that the informed consent documents and other materials could be understood by local stakeholders?**

| **Response:** Participant information sheets and consent processes were available in English, Krio, Mende, and Temne. All eight research assistants were Sierra Leoneans embedded in Sierra Leonean cultural contexts and collectively fluent in these languages. They explained the study verbally in each participant’s preferred language, covering study purpose, voluntary participation, confidentiality protections, potential emotional risks, right to decline any question, right to withdraw, and available referral support. Participants were given time to ask questions before consent was recorded. For participants who could not read, the information sheet was read aloud. For participants who could not write, witnessed thumbprint consent was obtained in accordance with SLESRC procedures. Interview guides were piloted and refined to improve clarity, cultural appropriateness, and sensitivity to the stigmatised nature of mpox. |
| --- |

**7. Will the findings of the research be made available in an understandable format to stakeholders in the community where the study was conducted, for example via a presentation, summary report, copies of publications, etc.? Please provide details of how this will be achieved.**

| **Response:** Yes. As the outbreak response continued, preliminary findings were shared with Sierra Leone public health stakeholders in formats appropriate to the sensitivity of the subject matter; further dissemination is planned following publication. Outputs have included and will include summaries for the NPHA, District Health Management Teams, participating facilities, and outbreak response stakeholders. These address the practical implications of the findings for stigma-informed outbreak response, survivor confidentiality, community-centred communication, contact tracing, vaccination communication, and healthcare worker support. All dissemination has been conducted with attention to participant confidentiality, avoiding information that could identify individuals, households, facilities, or small communities. Copies or summaries of the resulting publication will be made available to institutional stakeholders in Sierra Leone. |
| --- |

# Non-human subjects research using specimens/animals or archival collections

This study did not involve non-human subjects research, animals, imported specimens, archival specimens, cultural artefacts, archaeological material, botanical or zoological samples, or photographs of human remains.

**8. Did the permission you obtained from a local authority to perform the study include an agreement on access to outputs and benefit sharing?**

| **Response:** N/A. This study involved human subjects qualitative health research and did not involve specimens, animals, cultural artefacts, archaeological material, or archival collections. Local benefit is addressed through dissemination of findings to Sierra Leone public health stakeholders and direct application to outbreak response practice. |
| --- |

**9. If the material used in your study was imported, please provide the year it was imported, indicate whether permits were obtained to import/export the materials used, and provide details of any permits obtained. If this information is not available, please indicate this.**

| **Response:** N/A. No study material was imported or exported. |
| --- |

**10. If you used archival specimens, please state how the material used in your study was acquired by the institute it is held in and provide details of any permits obtained for the original excavations/sample collection. If this information is not available, please indicate this.**

| **Response:** N/A. No archival specimens were used. |
| --- |

**11. How was the potential cultural significance of the materials collected in your study to local communities considered in your research design? Were Indigenous peoples and/or local researchers and institutions involved with archaeological excavations/collection of specimens? If so, please provide a description of their involvement.**

| Response: N/A. The study did not involve archaeological, cultural artefact, botanical, or zoological materials. Cultural significance of qualitative data was addressed through local research leadership, language-appropriate consent processes, Sierra Leonean research assistants embedded in local cultural and linguistic contexts, confidentiality protections, sensitive interviewing, and careful reporting to avoid identifying participants in a highly stigmatised outbreak context. |
| --- |

**12. If your manuscript includes photographs of human remains, please indicate whether authors obtained permission from descendants or affiliated cultural communities to do so.**

| **Response:** N/A. The manuscript does not include photographs of human remains. |
| --- |
