# Supplementary material for: Suffering in silence: Stigma, healthcare barriers, and resilience during Sierra Leone’s 2025 clade IIb mpox outbreak—A multi-perspective qualitative study
Source: PLOS Glob Public Health. 2026 Jun 30;6(6):e0006686. doi: 10.1371/journal.pgph.0006686 (PMC13318003; doi:10.1371/journal.pgph.0006686)
Supplement: Attachment — Submitted filename: PGPH-D-26-00336_R1.docx [file pgph.0006686.s010.docx]

**PLOS Global Public Health**

# Suffering in silence: Stigma, healthcare barriers, and resilience during Sierra Leone's 2025 clade IIb mpox outbreak—A multi-perspective qualitative study

--Manuscript Draft--

| **Manuscript Number:** | PGPH-D-26-00336R1 |
| --- | --- |
| **Article Type:** | Research Article |
| **Full Title:** | Suffering in silence: Stigma, healthcare barriers, and resilience during Sierra Leone's 2025 clade IIb mpox outbreak—A multi-perspective qualitative study |
| **Short Title:** | Mpox stigma and healthcare experiences in Sierra Leone |
| **Corresponding Author:** | Eric Nzirakaindi Ikoona, MD, MMED, PhD, MPH National Public Health Agency  FREETOWN, SIERRA LEONE |
| **Order of Authors:** | Eric Nzirakaindi Ikoona, MD, MMED, PhD, MPH |
|  | Lucy Namulemo, MSc, PhD |
|  | Mary Magdalene Sinnah, MPH |
|  | Mohamed Alex Vandi, MD, MPH |
|  | Foday Sahr, MBChB, MMED, PhD |
| **Keywords:** | mpox, monkeypox, Sierra Leone, qualitative research, lived experience, stigma, healthcare workers, health systems, outbreak response, clade IIb |
| **Abstract:** | Background  Mpox was confirmed in Sierra Leone in January 2025, with a public health emergency declared shortly thereafter. By June 2025, the country reported over 4,000 confirmed cases caused by clade IIb. Limited qualitative research has explored the lived experiences of affected populations during mpox outbreaks in African settings. This study explored multi-stakeholder experiences of mpox, including illness trajectories, manifestations of stigma, healthcare-seeking behaviours, and challenges in health system responses.  Methods  We conducted a multi-perspective qualitative study using thematic framework analysis between March and August 2025 across four districts. Participants included mpox survivors (n=42), healthcare workers (n=38), community members (n=36), and contact tracers (n=24). Data were collected through 94 semi-structured interviews and 12 focus group discussions. Analysis was guided by a modified Social Ecological Model integrating the Health Stigma and Discrimination Framework.  Results  Five interconnected themes emerged: (1) illness characterised by physical suffering and lasting bodily changes; (2) multi-layered stigma operating across individual, interpersonal, community, and institutional levels; (3) healthcare-seeking shaped by fear of disclosure and economic constraints; (4) healthcare workers’ moral distress and professional isolation; (5) adaptive health system response alongside coordination challenges. Stigma operated as a cross-cutting dimension that intersected with all other themes, shaping illness experience, care-seeking behaviour, professional practice, and system-level response simultaneously.  Conclusions  Stigma permeated all dimensions of the mpox outbreak experience, delaying care- seeking, undermining contact tracing, and compounding healthcare worker distress. Compound trauma from sequential epidemic exposure emerged as a distinct burden among frontline workers. These findings point to the need for stigma-informed outbreak response strategies, sustained healthcare worker support mechanisms, and community-centred communication approaches in post-conflict, resource-constrained settings facing recurrent epidemics. |

*Powered by Editorial Manager® and ProduXion Manager® from Aries Systems Corporation*

| **Additional Information:** |  |
| --- | --- |
| **Question** | **Response** |
| **Financial Disclosure**  Enter a financial disclosure statement that describes the sources of funding for the work included in this submission and the role the funder(s) played. This includes grants and any commercial funding of the work or authors.  This statement will be typeset if the manuscript is accepted for publication.  *Please review the* [*submission guidelines*](http://journals.plos.org/globalpublichealth/s/submission-guidelines#loc-financial-disclosure-statement) *and the instructions link below for detailed requirements and guidance.* | The authors received no specific funding for this work. |
| **Competing Interests**  On behalf of all authors, disclose any competing interests that could be perceived to bias this work.  This statement will be typeset if the manuscript is accepted for publication.  *Please review the instructions link below and PLOS Global Public Health’s* [*competing interests*](https://journals.plos.org/globalpublichealth/s/competing-interests) *policy to determine what information must be disclosed at submission.* | The authors have declared that no competing interests exist. |
| <b>Data Availability</b>  <br><br>Before publication, Authors are required to make fully available and without restriction all data underlying their findings. Sharing the data underlying your findings increases the visibility, credibility, and impact of your work, while supporting transparency and future scientific discovery. Please see our <a href="https://journals.plos.org/globalpublic health/s/data-availability" target="_blank">PLOS Data Policy</a> page for detailed information on this policy. | De-identified qualitative data (full transcripts) cannot be publicly shared because they contain sensitive information and indirect identifiers related to a stigmatizing infectious disease outbreak context, and public release may risk participant re-identification given the small number of mpox cases in specific geographic areas during the study period. The study materials that support transparency and reproducibility are available in the Supporting Information files: interview and focus group discussion guides (S2–S3 Appendix), the full coding framework with inclusion/exclusion criteria (S4 Appendix), the completed COREQ checklist (S5 Appendix), and extended de-identified quotations mapped to themes (S6 Appendix). Researchers may request access to further de- identified data excerpts under a data-sharing agreement, subject to approval by the Sierra Leone Ethics and Scientific Review Committee. Requests should be directed to the corresponding author (Eric Nzirakaindi Ikoona, [ikoonae@yahoo.com)](mailto:ikoonae@yahoo.com) with a brief |

*Powered by Editorial Manager® and ProduXion Manager® from Aries Systems Corporation*

| <br><br>A <b>Data Availability Statement</b>, detailing where the data can be accessed, is required at first submission. Insert your Data Availability Statement in the box below.  <br><br>Please see the <a href="https://journals.plos.org/globalpublic health/s/submission-guidelines#loc-data- reporting" target="_blank">data reporting</a> section of our submission guidelines for instructions on what you need to include in your Data Availability Statement. Two examples of acceptable Data Availability Statements are included here for reference:  <ol>  <li>The minimal data set [and accompanying code, where generated] is available at [repository name] via [DOI hyperlink].  <li>No data was generated by this study. The following existing data sources were used: [dataset name] from [dataset location] available via [web address or DOI hyperlink]</li>  </ol>  <br><i>PLOS allows rare exemptions to address legal and ethical concerns. If you have legal or ethical restrictions, please use the box below to detail these in full sentences for the Journal team to consider.</i></p>  <br>This statement will be typeset if the manuscript is accepted for publication. | proposal describing the intended use and data protection safeguards. |
| --- | --- |

*Powered by Editorial Manager® and ProduXion Manager® from Aries Systems Corporation*

<https://www2.cloud.editorialmanager.com/pgph/download.aspx?id=799189&guid=f761d2fd-df7f-4fb1-9cf6-25c1cb5399ea&scheme=1>Manuscript Clean	[Click here to access/download;Manuscript;PGPH-D-26- 00336_Manuscript_Clean2_revised.docx](https://www2.cloud.editorialmanager.com/pgph/download.aspx?id=799189&guid=f761d2fd-df7f-4fb1-9cf6-25c1cb5399ea&scheme=1)

#### Suffering in silence: Stigma, healthcare barriers, and resilience during Sierra Leone’s 2025 clade IIb mpox outbreak—A multi-perspective qualitative study

**Short Title: Mpox stigma and healthcare experiences in Sierra Leone**

Eric Nzirakaindi Ikoona¹*, Lucy Namulemo²·³, Mary Magdalene Sinnah¹, Mohamed Alex Vandi¹, Foday Sahr¹

¹ National Public Health Agency, Freetown, Sierra Leone

² Foothills Community-Based Interventions, Monticello, Kentucky, United States

³ Uganda Counselling and Support Services, Kampala, Uganda

* Corresponding author

E-mail: [ikoonae@yahoo.com](mailto:ikoonae@yahoo.com) (ENI)

# Abstract

#### Background

Mpox was confirmed in Sierra Leone in January 2025, with a public health emergency declared shortly thereafter. By June 2025, the country reported over 4,000 confirmed cases caused by clade IIb. Limited qualitative research has explored the lived experiences of affected populations during mpox outbreaks in African settings. This study explored multi- stakeholder experiences of mpox, including illness trajectories, stigma manifestations, healthcare-seeking behaviours, and health system response challenges.

#### Methods

We conducted a multi-perspective qualitative study using thematic framework analysis between March and August 2025 across four districts. Participants included mpox survivors (n=42), healthcare workers (n=38), community members (n=36), and contact tracers (n=24). Data were collected through 94 semi-structured interviews and 12 focus group discussions. Analysis was guided by a modified Social Ecological Model (SEM) integrating the Health Stigma and Discrimination Framework (HSDF).

#### Results

Five interconnected themes emerged: (1) illness characterised by physical suffering and lasting bodily changes; (2) multi-layered stigma operating across individual, interpersonal, community, and institutional levels; (3) healthcare-seeking shaped by fear of disclosure and economic constraints; (4) healthcare workers’ moral distress and professional isolation; (5) adaptive health system response alongside coordination challenges. Stigma operated as a cross-cutting dimension that intersected with all other themes, shaping illness experience, care-seeking behaviour, professional practice, and system-level response simultaneously.

#### Conclusions

Stigma permeated all dimensions of the mpox outbreak experience, delaying care- seeking, undermining contact tracing, and compounding healthcare worker distress. Compound trauma from sequential epidemic exposure emerged as a distinct burden among frontline workers. These findings point to the need for stigma-informed outbreak response strategies, sustained healthcare worker support mechanisms, and community-

centred communication approaches in post-conflict, resource-constrained settings facing recurrent epidemics.

**Keywords:** mpox, monkeypox virus, Sierra Leone, qualitative research, lived experience, stigma, healthcare workers, health systems, outbreak response, clade IIb

# Introduction

Mpox (formerly monkeypox) remains a significant public health concern. The World Health Organization (WHO) declared the mpox upsurge a Public Health Emergency of International Concern (PHEIC) on 14 August 2024 [1]. Mpox was first confirmed in Sierra Leone on 10 January 2025, the outbreak was officially declared on 13 January 2025, and a national public health emergency was declared on 16 January 2025 [2,3]. By 22 June 2025, the country reported 4,294 confirmed cases and 28 deaths [4]. In early May 2025, Sierra Leone accounted for approximately half of Africa’s confirmed mpox cases, underscoring the intensity of transmission during the outbreak acceleration phase [5].

The Sierra Leone outbreak presents distinctive epidemiological and social characteristics. Genomic evidence indicates that most sequences fall within clade IIb and cluster as a distinct group descended from lineage A.2.2, consistent with sustained human-to-human transmission; a proposed designation identifies this newly observed Sierra Leone lineage as G.1 (alias of A.2.2.1) [6]. Frequent genital involvement among cases supports the plausibility of sexual networks contributing to clade IIb transmission in this setting [7,8]. Sexual exposure histories are socially and legally sensitive, and self-reported accounts of exposure have raised the possibility of underreporting, stigma, and disclosure constraints [8]. These epidemiological and social features carry significant implications for understanding disease spread, designing targeted interventions, and addressing the

complex social dimensions of the outbreak.

### The Sierra Leone context

Sierra Leone’s mpox response occurs within a health system shaped by post-conflict rebuilding and the 2014–2016 Ebola epidemic, which disrupted services and workforce capacity [9,10]. More recently, the country navigated the COVID-19 pandemic while simultaneously addressing endemic challenges, including malaria, tuberculosis, and HIV [8]. These cumulative experiences have shaped both the health system’s adaptive capacity and community perceptions of outbreak response, creating a distinctive context in which trust, institutional memory, and resource constraints interact in ways that may differ from settings without comparable histories of sequential health emergencies.

The concentration of cases in the Western Area, encompassing Freetown and surrounding districts, reflects underlying patterns of urbanisation, population density, and healthcare access [4]. By mid-2025, four dedicated treatment centres had opened, a 400-bed facility had become operational in Freetown, and over 43,000 individuals had received vaccination— yet these resources remained insufficient for the scale of the epidemic [4,11].

### Stigma and infectious disease outbreaks

Infectious disease outbreaks have historically been accompanied by stigmatisation of affected individuals and communities [12,13]. Link and Phelan’s foundational conceptualisation describes stigma as occurring when elements of labelling, stereotyping, separation, status loss, and discrimination converge within a context of power imbalance [14]. Disease-related stigma manifests through multiple mechanisms: enacted stigma (actual experiences of discrimination), perceived stigma (expectations of mistreatment),

internalised stigma (acceptance of negative societal attitudes), and associative stigma (stigma experienced by those connected to affected individuals) [15].

The mpox outbreak presents particular stigma challenges given its visible cutaneous manifestations, perceived associations with sexual transmission, and historical framings as an “African disease” [16,17]. Evidence from the 2022 global outbreak documented significant stigma among affected populations, including personalised stigma, negative self-image, and disclosure concerns [18,19]. Healthcare workers have similarly reported fear of contagion and social isolation [20,21]. While these dynamics are well documented in high-income country contexts, significant gaps remain in understanding how mpox- related stigma operates in African settings, where different cultural meanings, health system structures, and prior outbreak experiences may produce distinct stigma configurations [22].

### Healthcare workers' experiences in outbreak response

Healthcare workers (HCWs) occupy a critical yet vulnerable position during infectious disease outbreaks [23]. Research from the 2022 mpox response documented knowledge gaps, with 30% of HCWs having never heard of mpox before the outbreak and over one- quarter initially misdiagnosing cases [24]. HCWs reported inadequate access to vaccines, concerns about occupational exposure, and moral distress related to resource constraints [25]. The psychological toll of outbreak response—compounded for many by the recent COVID-19 pandemic—has contributed to burnout, compassion fatigue, and professional attrition [26,27].

### Gaps in current knowledge

Despite growing epidemiological surveillance of mpox in Africa, qualitative research examining lived experiences remains markedly limited [28,29]. To our knowledge, no published qualitative studies have examined mpox experiences from endemic African settings during the current PHEIC period. Existing qualitative research has predominantly emerged from high-income country contexts, primarily focusing on men who have sex with men populations affected by clade IIb during the 2022 outbreak [18,19,30]. This leaves significant knowledge gaps regarding how mpox illness is experienced in African contexts, the specific manifestations and mechanisms of mpox-related stigma in these settings, and healthcare-seeking behaviours and barriers in resource-constrained environments.

### Study aims and theoretical framework

This study aimed to provide a comprehensive qualitative exploration of mpox outbreak experiences in Sierra Leone, addressing the following research questions: (1) How do individuals who have experienced mpox understand and narrate their illness trajectories?

(2) What forms and mechanisms of stigma emerge during mpox outbreaks, and how do these affect individuals, families, and communities? (3) What factors shape healthcare-seeking behaviors among those affected by or at risk of mpox? (4) How do healthcare workers and public health personnel experience outbreak response? (5) How do experiences vary across different social positions and geographic contexts?

Our analysis was guided by a modified Social Ecological Model (SEM) integrating the Health Stigma and Discrimination Framework (HSDF) [15,31] (S1 Appendix). The SEM

provides a multi-level structure for examining individual, interpersonal, community, organisational, and policy factors that shape health experiences. The HSDF offers a cross- cutting framework for understanding how stigma operates through marking, labelling, separation, status loss, and discrimination within specific contexts. We employed these frameworks as sensitising tools rather than rigid analytical templates, remaining open to findings that might challenge, extend, or complicate the frameworks’ assumptions— particularly regarding the interplay between structural constraints and individual-level processes in a post-conflict, resource-constrained setting.

# Methods

### Ethics Statement

Ethical approval was obtained from the Sierra Leone Ethics and Scientific Review Committee (SLESRC-2025-049) on 28 February 2025. All participants provided written informed consent before participation. For participants who could not write, witnessed thumbprint consent was obtained in accordance with SLESRC guidance. The consent process covered the study purpose, procedures, voluntary participation, the right to withdraw, confidentiality protections, potential emotional risks, referral options, audio recording, data storage, dissemination plans, and contact information for the research team and the ethics committee.

Given the potentially stigmatising nature of mpox, confidentiality protections were central to the study design. Participants selected interview locations that offered privacy; identifiers were removed during transcription; quotations are presented using pseudonyms, and with identifying details altered; and data files are stored on encrypted, password-protected servers accessible only to the research team. Emotional distress protocols were established, and research assistants were trained to recognise distress

signals and offer supportive responses. Referral pathways to mental health services were established in each district through a partnership with Partners in Health Sierra Leone. Participants received transportation reimbursement (NLE 50, approximately USD 2.50 at the June 2025 exchange rate) and refreshments, but no other compensation, to minimise coercion.

### Study design and period

We conducted a multi-perspective qualitative study using thematic framework analysis to examine lived experiences of the mpox outbreak [32,33]. The study was conducted between 10 March and 28 August 2025, spanning the outbreak’s acceleration, peak (May– June 2025), and initial decline phases. Thematic framework analysis is well-suited for policy-relevant health research involving multiple participant groups, enabling systematic comparison across cases while maintaining analytic transparency [32,34]. Our analytical approach was informed by the SEM and the HSDF, which provided sensitising concepts for theme development [15,31].

We incorporated multiple stakeholder perspectives to triangulate and develop a comprehensive understanding of the outbreak experience. This multi-perspective approach aligns with calls for qualitative research that moves beyond single-stakeholder views to capture the relational and systemic dimensions of health phenomena [35]. The four participant groups—survivors, healthcare workers, community members, and contact tracers—occupy distinct epistemic positions. Survivors provide experiential knowledge grounded in embodied illness experience; healthcare workers contribute professional and observational accounts shaped by clinical training and institutional roles; community members offer perspectives on social responses and collective meanings; and contact tracers provide operational insights from the interface between surveillance systems and community engagement. We treated these perspectives as

complementary but epistemically distinct, and our analytical approach was designed to differentiate between lived experience and professional or observational discourse (see Data analysis). The study followed the Consolidated Criteria for Reporting Qualitative Research (COREQ) guidelines [36] (S5 Appendix).

### Setting

The study was conducted across four districts representing diverse epidemiological and geographic contexts: Western Area Urban (Freetown), Western Area Rural, Bo, and Kenema. These districts were selected based on: (1) case burden, with Western Area accounting for approximately 80% of confirmed cases; (2) geographic diversity, including urban, peri-urban, and rural settings; (3) healthcare infrastructure variation, from tertiary facilities to community health centres; and (4) prior outbreak experience, with all districts having been affected by the 2014–2016 Ebola epidemic [9].

At the time of data collection, the four districts exhibited distinct epidemiological profiles. Western Area Urban reported the highest case concentration, driven by population density and social mixing patterns characteristic of the capital. Western Area

Rural, while geographically proximate, exhibited different transmission dynamics associated with peri-urban settlement patterns and more limited healthcare access. Bo and Kenema districts in the south and east reported lower overall case counts but faced particular challenges related to diagnostic capacity, delayed specimen transport, and greater distances to treatment centres. Across all four districts, cases were predominantly among young adults aged 15–35 years, with males predominating among reported cases [7].

### Participants and sampling

We recruited 140 participants across four stakeholder groups: mpox survivors who had completed isolation (n=42), healthcare workers involved in mpox response (n=38), community members from affected areas (n=36), and contact tracers/surveillance officers (n=24). Purposive sampling with maximum variation was employed to ensure diversity across demographic characteristics (age, gender, education), geographic location (urban/peri-urban/rural), and, for survivors, illness severity and time since recovery.

Inclusion criteria for survivors required: confirmed mpox diagnosis (laboratory or clinical), completion of isolation period, age 18 years or older, capacity to provide informed consent, and willingness to discuss experiences. Healthcare worker eligibility required direct involvement in mpox clinical care, laboratory services, or infection prevention and control. Community member eligibility required residence in a community with documented mpox cases. Contact tracers required current or recent employment in mpox surveillance activities.

### Participant recruitment and contact

Recruitment utilised multiple pathways tailored to each stakeholder group. For survivor participants, treatment centre staff at the four designated mpox treatment facilities (Connaught Hospital, 34 Military Hospital, Kenema Government Hospital, and Bo Government Hospital) provided study information sheets to patients nearing discharge. Patients who expressed interest were referred to research assistants, who contacted them via telephone 2–4 weeks post-discharge to confirm eligibility and schedule interviews. Community health workers also identified survivors in their catchment areas who had completed home-based isolation.

Healthcare workers were recruited through facility-based approaches. Research team members visited hospitals, peripheral health units, and laboratories involved in the mpox response to present the study at staff meetings. Interested staff provided contact information and were subsequently contacted to arrange interviews at convenient times. Contact tracers were recruited through District Health Management Teams (DHMTs); surveillance focal persons distributed study information during routine team meetings, and interested personnel were contacted directly by research assistants.

Community members were identified through community leaders and existing community health structures. Community Health Officers introduced the study at community meetings in areas with documented mpox cases. Interested community members provided contact details and were subsequently contacted to arrange participation. We assessed theme stability iteratively within each stakeholder group and district; recruitment stopped when additional interviews did not generate new codes or meaningfully extend existing themes.

### Data collection

Data collection employed semi-structured interviews (SSIs) and focus group discussions (FGDs), with methods matched to participant group, preferences, topic sensitivity, and feasibility. Individual SSIs were preferred for survivors because illness narratives, perceived sexual exposure, stigma, and family or workplace disclosure were highly sensitive. For healthcare workers, SSIs were used to elicit private accounts of moral distress, resource constraints, occupational risk, and emotional burden, while FGDs were used to explore shared operational experiences, collective reflections, and points of convergence or disagreement across professional roles.

In total, we conducted 94 SSIs (42 with survivors, 28 with healthcare workers, 12 with community members, 12 with contact tracers) and 12 FGDs (4 with healthcare workers, 6 with community members, 2 with contact tracers). FGDs comprised 6–10 participants each, grouped by stakeholder type and district to enable comfortable discussion among peers with shared experiences. For healthcare worker FGDs, we attempted to group participants by cadre or level of seniority where feasible, but full stratification was not always possible because of small numbers of eligible staff at some facilities, particularly in Bo and Kenema.

Because FGDs in clinical settings can suppress disclosure, facilitators monitored group dynamics, invited quieter participants to speak, reminded participants that disagreement was acceptable, and avoided questions that required disclosure of identifiable information about colleagues or patients. Where participants appeared uncomfortable or where sensitive issues required more private exploration, follow-up SSIs were offered. We therefore interpret healthcare worker FGDs as collective professional discourse and place greater evidentiary weight on SSIs for sensitive individual experiences.

Interview guides were developed separately for each stakeholder group, informed by the

theoretical framework and existing literature (S2 and S3 Appendices). Survivor guides explored illness onset and trajectory, healthcare-seeking experiences, social responses and stigma, coping strategies, and perspectives on recovery. Healthcare worker guides examined clinical experiences, occupational challenges, resource constraints, emotional impacts, and recommendations. Community member guides explored knowledge and perceptions, community responses, fears and concerns, and recommendations. Contact tracer guides examined work experiences, challenges encountered, community responses, and support needs.

All guides were piloted with five individuals from each stakeholder group and refined based on feedback. Interviews were conducted in Krio, Mende, Temne, or English, according to participant preference, by trained Sierra Leonean research assistants fluent in the local languages used in the study. The research assistants were embedded in Sierra Leonean cultural contexts and, when needed, explained study procedures and sensitive concepts in locally meaningful terms. Interviews averaged 62 minutes for survivors (range: 45–95 minutes), 48 minutes for other SSIs (range: 35–75 minutes), and 87 minutes for FGDs (range: 72–110 minutes). All sessions were audio-recorded with permission and transcribed verbatim in the original language before being translated into English by bilingual team members.

Translations were verified by back-translating randomly selected segments (20% of transcripts).

### Informed consent process

The informed consent process was designed to ensure voluntary, informed participation while being sensitive to the stigmatising nature of mpox. Prior to each interview or FGD, research assistants met with potential participants in a private setting of their choosing (typically at their home, in a private room at a health facility, or at a community centre).

Research assistants explained the study using a standardised information sheet available in English, Krio, Mende, and Temne.

The consent process covered: (1) study purpose and procedures, including the expected duration and topics to be discussed; (2) voluntary nature of participation, with explicit emphasis that declining would not affect access to healthcare or other services; (3) right to withdraw at any time without penalty; (4) right to skip any question or stop the interview at any time; (5) confidentiality protections, including use of pseudonyms, secure data storage, and removal of identifying information; (6) potential risks including emotional distress when discussing difficult experiences; (7) available support including referral to mental health services if needed; (8) audio-recording procedures and data storage; (9) plans for data use and dissemination; and (10) contact information for the research team and ethics committee for questions or concerns.

Participants were given time to ask questions and were provided with the information sheet to keep. For participants who could not read, the information sheet was read aloud in their preferred language. Written informed consent was obtained from all participants before data collection commenced. For participants who could not write, witnessed thumbprint consent was obtained in accordance with Sierra Leone Ethics and Scientific Review Committee guidelines. A copy of the signed consent form was provided to each participant.

### Research team and reflexivity

The research team comprised investigators with backgrounds in public health, medicine, nursing, medical anthropology, and community health. The study was locally led from within Sierra Leone's public health system and institutionally anchored in the National Public Health Agency (NPHA), the national institution responsible for public health protection, surveillance, outbreak preparedness and response, and evidence generation to understand drivers of public health threats. The principal investigator (ENI) and co- principal investigator (FS) are physicians and public health researchers based in Sierra Leone with extensive experience in qualitative health research and implementation science. FS is the Executive Director, and ENI is the Strategic Advisor of the NPHA. The co- investigators, MAV and MMS, are Sierra Leonean public health professionals with direct experience in Sierra Leone’s outbreak response architecture, surveillance systems, and community-facing public health practice. MAV is a physician and senior public health leader within the NPHA, and MMS is a Principal Surveillance Officer with experience in national surveillance and outbreak response.

LN is a doctorate-trained social scientist and mental health expert with training in qualitative methods and prior experience in HIV and infectious disease research in East and West Africa. Research assistants (n=8) were recruited from Sierra Leone; all were held at

minimum a bachelor’s degree in a health-related field, and four had prior experience in qualitative data collection during the Ebola response. Research assistants received five days of training covering qualitative methods, ethical considerations, sensitive interviewing, provision of emotional support, and mpox-specific knowledge. The team included members with prior experience in Ebola response research, providing valuable contextual understanding [9,10].

We maintained reflexive practices throughout, recognising that researchers’ positions—as healthcare professionals, public health practitioners, and Sierra Leoneans navigating the same outbreak—shaped data collection and interpretation. Weekly team debriefings addressed emerging insights, analytic decisions, and the researcher's well-being. Reflexive memos documented team members’ evolving understandings, emotional responses, and methodological considerations.

### Inclusivity in global research

Additional information regarding ethical, cultural, and scientific considerations specific to inclusivity in global research is included in the Supporting Information (S7 Appendix). This study was conceived, approved, implemented, analysed, and interpreted from within Sierra Leone’s public health system. It was not organised as a short-term external or visiting research project. The inclusion of LN reflected complementary qualitative and mental health expertise, while study leadership, institutional authority, field implementation, and interpretation remained grounded in Sierra Leonean public health institutions and local research personnel.

### Data analysis

Analysis followed the Framework Method, enabling systematic cross-case comparison and transparent matrix-based synthesis [32,33]. Analysis proceeded through six phases:

Familiarisation: Analysts read complete transcripts multiple times, noting initial

observations; (2) Developing an analytical framework: Initial coding of a subset of transcripts to develop a working codebook informed by the SEM/HSDF theoretical framework and emergent concepts; (3) Indexing: Systematic coding of all transcripts using the agreed codebook; (4) Charting: Summarising coded data into framework matrices organised by theme and participant group; (5) Cross-case analysis: Identifying patterns and variations across participants within and between stakeholder groups; (6) Interpretation: Developing higher-order interpretations connecting findings to broader literature and implications.

Because stigma formed part of the study's aims and the HSDF sensitising framework, the analysis did not treat the mere presence of stigma as wholly inductive. The primary analytical question was not whether stigma existed, but what forms and mechanisms of stigma emerged and how these affected individuals, families, communities, healthcare workers, contact tracing, and system response. We therefore distinguished between framework-informed domains and data-led extensions of those domains during coding, charting, and interpretation.

A critical analytical decision concerned how to integrate and differentiate perspectives across the four participant groups. We constructed separate framework matrices for each stakeholder group before conducting cross-group analysis, ensuring that patterns within each group were identified before comparisons were drawn. In our interpretive process, we explicitly distinguished between first-person experiential accounts (primarily from survivors), professional observational accounts (primarily from healthcare workers and contact tracers), and community normative perspectives (primarily from community members). Where healthcare workers or contact tracers spoke about patient experiences,

we treated these as institutionally situated observations rather than direct evidence of lived experience, and we note this distinction in our presentation of findings.

Convergence across groups was interpreted as strengthening credibility, while divergence prompted further analytical interrogation of the epistemic basis for differing accounts.

Data management was conducted using NVivo 14 (Lumivero, 2023). Coding was led by ENI and LN, both of whom had doctoral-level training in qualitative research methods. ENI brought embedded knowledge of Sierra Leone’s health system and outbreak response expertise, while LN brought a comparative perspective from qualitative research experience in East African settings. A team-based approach to coding was employed, with initial independent coding by both analysts followed by consensus discussions to resolve discrepancies. An audit trail documented all analytic decisions (S4 Appendix). Subgroup analyses examined variations by gender, age group (18–24, 25–34, 35–49, 50+), district, urban/rural residence, education level, and, for healthcare workers, professional role and facility type. Given the qualitative nature of the study and modest subgroup sizes, we report observed patterns as exploratory observations rather than generalisable subgroup differences, and we exercise caution in interpretation where cell sizes are small. We also attended to how social positions may intersect—for example, how gender and rural residence jointly shaped stigma experiences—rather than treating demographic categories as independent, parallel variables. Member checking was conducted with 20 participants (5 from each stakeholder group) who reviewed preliminary findings and provided feedback that was incorporated into the final analysis.

# Results

### Participant characteristics

Table 1 presents participant demographics. Among the 42 mpox survivors, 26 (61.9%) were male and 16 (38.1%) were female, with a mean age of 28.4 years (range: 18–52). The majority resided in Western Area Urban (n=25, 59.5%), followed by Western Area

Rural (n=9, 21.4%), Bo (n=5, 11.9%), and Kenema (n=3, 7.1%). Time since recovery ranged from 2–14 weeks at interview. Among healthcare workers (n=38), 22 (57.9%) were nurses, 8 (21.1%) were physicians, 5 (13.2%) were laboratory technicians, and 3 (7.9%) were IPC officers. Community members (n=36) were distributed across all four districts with deliberate oversampling from communities with the highest case burdens. Contact tracers (n=24) represented all DHMTs involved in the mpox response.

#### Table 1. Demographic characteristics of study participants.

| **Characteristic** | **Survivors**  **(n=42)** | **Healthcare workers**  **(n=38)** | **Community**  **(n=36)** | **Contact Tracers**  **(n=24)** |
| --- | --- | --- | --- | --- |
| Gender – Male | 26 (61.9%) | 15 (39.5%) | 18 (50.0%) | 16 (66.7%) |
| Gender – Female | 16 (38.1%) | 23 (60.5%) | 18 (50.0%) | 8 (33.3%) |
| Age (mean, range) | 28.4 (18–52) | 36.2 (24–58) | 38.7 (22–65) | 32.1 (23–48) |
| Western Area  Urban | 25 (59.5%) | 22 (57.9%) | 14 (38.9%) | 10 (41.7%) |
| Western Area  Rural | 9 (21.4%) | 8 (21.1%) | 10 (27.8%) | 6 (25.0%) |
| Bo District | 5 (11.9%) | 5 (13.2%) | 6 (16.7%) | 4 (16.7%) |
| Kenema District | 3 (7.1%) | 3 (7.9%) | 6 (16.7%) | 4 (16.7%) |

Analysis identified five overarching themes with multiple subthemes, summarised in the thematic framework (S1 Fig), with the full diagram and explanation provided in S1

Appendix. Distinct mechanisms through which stigma manifested appeared across all five themes. We present each theme with illustrative quotations and indicate participant characteristics using codes (e.g., S-M-28-WAU = survivor, male, age 28, Western Area Urban). Extended quotations supporting each theme and subtheme appear in S6 Appendix.

### Theme 1: “The body that betrays”—Illness experiences

Survivors described illness experiences characterised by physical suffering, diagnostic uncertainty, and lasting bodily changes. This theme encompassed four subthemes: onset and recognition, physical suffering, diagnostic journeys, and altered body experience.

#### Onset and recognition

Initial symptom recognition was frequently delayed or misattributed, particularly in the outbreak’s early phases. Many survivors described initially interpreting symptoms through familiar illness categories—malaria, skin infections, or sexually transmitted infections:

*“I first felt the fever coming and thought, this is malaria again. I took the [antimalarial] tablets and waited. When the bumps started appearing on my skin, I thought maybe it was an allergic reaction. It was only when my friend told me about mpox being in Freetown that I started to worry it could be that.” (S-M-24- WAU)*

#### Physical suffering

Survivors described intense physical suffering that often exceeded their expectations. Pain was a dominant feature, particularly associated with lesions, lymphadenopathy, and systemic symptoms. Survivors frequently reported limited access to effective pain relief (S6 Appendix):

*“The pain was like nothing I have experienced before. Every lesion felt like fire burning on my skin. I could not sleep, I could not eat, and I could not even lie down comfortably. The lymph nodes under my arms were so swollen that I could not lower my arms to my sides. I thought I was going to die.” (S-M-35-WAU)*

#### Diagnostic journeys

Pathways to diagnosis were often circuitous, involving multiple healthcare encounters. Several survivors described initial misdiagnosis:

*“The first clinic I went to, they said it was chickenpox and gave me calamine lotion. After two days, I went back because it was getting worse, and they said maybe it was syphilis and gave me injections. It was only when I went to [government hospital] that they finally said it could be mpox and took the swab.” (S-M-29-WAU)*

#### Altered body experience

Beyond acute illness, survivors described lasting changes in their relationship with their bodies, including scarring and impacts on sexuality and intimacy:

*“Since I recovered, I have not been able to be intimate with my husband. Every time I think about it, I remember the sores in that area, and I feel afraid and*

*ashamed. He is patient with me, but I can see he is frustrated. I do not know if this feeling will ever go away.” (S-F-34-WAU)*

### Theme 2: “Marked and marginalised”—Multi-layered stigma

Distinct mechanisms through which stigma manifested emerged across all participant groups and study sites. Stigma operated at multiple levels and through four interconnected forms: enacted stigma and discrimination, perceived stigma and anticipated rejection, internalised stigma and self-blame, and associative stigma (see S6 Appendix).

#### Enacted stigma and discrimination

Survivors reported direct experiences of discrimination from multiple sources, including family rejection, community avoidance, and healthcare discrimination:

*“When my family found out I had mpox, my own brother told me I could not stay in the house anymore. He said I would infect his children. I had to go and stay with a friend who was the only one willing to take me in.” (S-M-26-WAR)*

#### Perceived stigma and anticipated rejection

Perceived stigma shaped behaviour, particularly around disclosure:

*“I told no one except my mother and one close friend. Not even my employer—I said I had malaria and needed rest. I was so afraid that if people knew, I would lose everything.” (S-F-27-BO)*

#### Internalised stigma and self-blame

Many survivors described accepting negative social messages, leading to shame and self- blame, particularly when illness was attributed to sexual behaviour:

*“I kept asking myself, what did I do to deserve this? I felt so dirty, so ashamed. I thought maybe God was punishing me for something I had done. I could not even look at myself in the mirror.” (S-M-25-WAU)*

#### Associative stigma

Stigma extended to families, caregivers, and those associated with outbreak response. Both survivors and healthcare workers described this pattern. Healthcare workers reported being avoided by neighbours and community members because of their roles:

*“My neighbours know I work at the mpox treatment centre. Some of them have started avoiding me. One woman actually told me she does not want me coming near her children because I might bring the disease to the community. It is hurtful because I am trying to help people.” (HCW-F-34-WAU-Nurse)*

### Theme 3: “Seeking care in silence”—Healthcare-seeking trajectories

Multiple factors influenced healthcare-seeking behaviours, including stigma, knowledge, economic constraints, and health system accessibility. Three subthemes characterised these trajectories: delays and barriers, alternative care pathways, and facilitators of timely care (S6 Appendix).

#### Delays and barriers

Fear of stigma was the most frequently cited reason for delayed care-seeking:

*“I waited because I was afraid of what would happen if people found out. I thought maybe if I just stayed home, it would go away on its own, and no one would ever need to know.” (S-M-22-WAU)*

Care-seeking delays varied across social position and geography, but these observations were exploratory rather than comparative. Urban participants more often described concealment strategies shaped by fear of disclosure at work or within dense social networks. Rural participants more often described transport costs, distance to facilities, and limited access to diagnostic services. Participants in formal employment described fear of workplace disclosure and job loss, while those in informal work emphasised income loss from missed daily labour. Because several subgroups were small, particularly outside the Western Area, these patterns are presented narratively rather than as subgroup comparisons.

#### Facilitators of timely care

Survivors who sought care promptly described factors that facilitated their decision, including awareness of mpox symptoms from public health messaging, social support, and prior positive healthcare experiences:

*“I had seen the messages on the radio about mpox and what signs to look for. When I started having those exact symptoms, I knew I should go get tested right away.” (S-M-33-WAU)*

### Theme 4: “Working at the edge”—Healthcare worker experiences

Healthcare workers described experiences of responding to the outbreak while navigating resource constraints, occupational risks, emotional burdens, and community pressures.

Four subthemes captured these experiences (S6 Appendix).

#### Clinical challenges and moral distress

Healthcare workers described confronting clinical situations for which they felt

inadequately prepared:

*“There were times when we had more patients than beds. We had to make difficult decisions about who could be admitted and who had to go home. Some patients we sent home were very sick, but we had no space for them. I would lie awake at night wondering if I had made the right choice.” (HCW-M-38-WAU- Physician)*

#### Emotional toll and compound trauma

The emotional burden was substantial. Many healthcare workers who had served during previous epidemics described the mpox response reactivating earlier distress:

*“When mpox started spreading, it brought back all those memories from Ebola. The fear, the death, watching colleagues get sick. I thought I had processed all of*

*that but it came flooding back. Some nights I could not sleep.” (HCW-F-45-WAU- Nurse)*

### Theme 5: “Building the plane while flying”—Health system response

The final theme addresses the health system response, drawing primarily on accounts from healthcare workers and contact tracers. Participants described a response that was adaptive but strained, with frontline teams often adjusting referral pathways, communication practices, and community engagement strategies as the outbreak evolved. Four subthemes characterised this domain: adaptive capacity and improvisation, coordination challenges, vaccination experiences, and contact tracing barriers (S6 Appendix).

#### Adaptive capacity and improvisation

Frontline teams described adjusting their work in real time as case numbers increased and formal systems struggled to keep pace.

*“There was no perfect system at the beginning. Every day the situation changed, so we also changed how we worked. Sometimes the official message came late, and we used phone calls, WhatsApp groups, and people we trusted in the community to move faster. It was not ideal, but if we waited for everything to be arranged, the cases would move ahead of us.” (CT-M-32-WAU)*

Beyond communication improvisation, frontline adaptation extended to treatment capacity management. Healthcare workers described facilities that reached capacity rapidly during the outbreak’s acceleration phase, requiring informal arrangements for patient transfers between centres, staff rotation, and triage of cases for home-based isolation when admission was not possible. Referral pathways were negotiated in real time rather than through formal protocols, and transport availability—for both patients and specimens—was a recurrent constraint that affected the speed of both clinical response and surveillance (S6 Appendix).

#### Coordination challenges

Participants described delays and fragmentation in information flow across laboratory, surveillance, treatment, and field teams.

*“Sometimes the laboratory result, the case list, and the field team were not moving at the same speed. A patient could be confirmed, but by the time the information reached us, the family had already moved or the contacts were no longer easy to find. The problem was not that people refused to work. The problem was that the information was coming in pieces.” (CT-F-29-WAR)*

Transport constraints compounded information delays. Specimen movement between collection points and laboratories relied on vehicles, which were not always prioritised for surveillance. Contact tracers in rural and peri-urban areas described situations in which the window for identifying and isolating contacts had passed by the time

laboratory confirmation reached field teams. These gaps were understood by participants as systemic failures of coordination rather than individual lapses, but they accumulated into measurable delays in the response chain (S6 Appendix).

#### Vaccination experiences

Vaccination was described as protective, but also socially sensitive in communities where mpox was associated with shame, exposure, or sexual transmission.

*“People wanted protection, but they were also afraid of what others would think if they joined the vaccination line. Some said, ‘If they see me there, they will say I have been exposed.’ So the vaccine itself became connected to the same shame as the disease. We had to explain that vaccination was protection, not a confession.” (HCW-F-36-WAU-Nurse)*

Vaccine prioritisation and eligibility also created operational and social tension. Initial supplies were limited, and allocation decisions—across healthcare workers, close contacts, and community members—were experienced as inconsistent. Healthcare workers at peripheral facilities described receiving later access to vaccination than those at referral centres, which they perceived as increasing their occupational exposure during the outbreak’s early phase. Community members described uncertainty about eligibility criteria: some understood vaccination as reserved for confirmed contacts, while others were unsure whether seeking vaccination required disclosure of potential exposure. These ambiguities, compounded by the social stigma attached to vaccination in some communities, limited uptake among those who might have benefited most (S6 Appendix).

#### Contact tracing barriers

Contact tracing was particularly difficult when naming contacts could expose sexual relationships, household conflict, workplace absence, or socially disapproved behaviour.

*“Many patients are reluctant to name their contacts, especially if the contact was through sexual activity. They are afraid of what will happen to those people, or they do not want to reveal their private behaviour.” (CT-M-34-WAU)*

Beyond sexual exposure, contact tracers described how household tensions, employment concerns, and community visibility created additional barriers. Participants in informal employment feared that being named as a contact would render their illness visible to employers or community leaders, threatening their livelihoods. In some cases, contact tracers described extending timelines informally to allow participants to disclose to family members before notification proceeded, balancing surveillance objectives against participant safety and consent (S6 Appendix).

### Subgroup variations

Across the findings, subgroup variation appeared suggestive but not definitive. Female survivors more frequently described internalised stigma linked to perceived sexual transmission and intimate relationships, while male survivors more often emphasised economic impacts and work disruption. Younger adults described peer-level stigma, social media disclosure fears, and economic vulnerability. Older participants more often described family-based discrimination and concerns compounded by pre-existing health conditions. Rural participants generally described longer care-seeking pathways, whereas urban participants described more elaborate concealment strategies. These observations are exploratory and should not be interpreted as generalisable subgroup differences.

# Discussion

This study provides an in-depth qualitative exploration of mpox outbreak experiences in Sierra Leone, revealing physical suffering, multi-layered stigma, healthcare-seeking challenges, healthcare worker distress, and health system response dynamics during the clade IIb epidemic. While certain themes, including disease-related stigma, healthcare-

seeking delays, and healthcare worker moral distress, resonate with prior research on infectious disease outbreaks, our findings contribute empirical and conceptual insights that extend beyond contextual confirmation of known patterns [28,29].

Three contributions merit particular emphasis. First, this study provides what is, to our knowledge, the first qualitative account of mpox illness experiences from an African setting during the current PHEIC, offering empirical grounding for understanding how mpox is experienced in a context characterised by post-conflict health system fragility, sequential epidemic exposure, and cultural frameworks distinct from those documented in high-income country studies of the 2022 outbreak. Second, our multi-perspective design reveals how stigma operates not merely as a barrier at discrete points in the care pathway but as a recursive structural process that simultaneously shapes illness experience, healthcare-seeking, professional practice, contact tracing, and system-level response. Third, the phenomenon of compound trauma among healthcare workers, where mpox response reactivated unresolved distress from Ebola and COVID-19 experiences, represents a distinctive finding with implications for workforce sustainability in settings with histories of sequential health emergencies.

### Illness experiences and disrupted embodiment

Survivors’ narratives of physical suffering align with emerging clinical literature documenting severe pain as a hallmark of mpox, particularly when genital lesions are present [7,37]. Our qualitative findings add experiential depth to these clinical observations, revealing how inadequate pain management compounded suffering and eroded trust in healthcare. The phenomenon of “disrupted embodiment”—fundamentally altered relationships with one’s own body—has been documented in other illness contexts but has not previously been explored in relation to mpox [38]. Visible scarring and impacts on sexuality represent lasting consequences extending well beyond acute illness

resolution. It should be noted, however, that our cross-sectional design captures experiences at a single point in recovery; longitudinal research would be needed to understand how embodied experiences evolve over time and whether the disruptions described here are enduring or gradually resolve [18].

### Stigma mechanisms and impacts

Our findings extend the HSDF to the mpox context, documenting how stigma operates through multiple mechanisms—enacted, perceived, internalised, and associative—at multiple levels from individual to institutional [15]. The visible nature of mpox lesions, coupled with perceived associations with sexual transmission, creates a particularly stigmatising configuration [16,39]. Comparisons to HIV disease were explicit in participant accounts, echoing patterns documented in the 2022 global outbreak [18,19,22].

Critically, our findings illustrate how stigma undermined outbreak control in this setting. Fear of stigma delayed care-seeking, enabling ongoing transmission. Anticipated discrimination reduced disclosure to contacts, hampering contact tracing. Internalised stigma affected mental health and recovery. These pathways indicate that stigma warrants attention not only as a social harm but also as a public health concern integral to the effectiveness of outbreak response [12,22,40].

### Theoretical implications

Our findings have implications for the theoretical frameworks that guided the analysis. The study did not seek to establish whether stigma existed during the outbreak; rather, it examined the forms, mechanisms, pathways, and effects by which stigma shaped lived experience and response practices. This distinction matters theoretically because the empirical contribution lies in the movement of stigma across levels, not in its presence as a theme. The SEM provided a useful heuristic for organising multi-level findings, but the

empirical data suggest that relationships between levels are more dynamic and recursive than the model’s nested structure implies. Stigma did not operate as a discrete factor at any single level. It functioned as a cross-cutting structural force that simultaneously shaped individual cognition, interpersonal relationships, community norms, healthcare encounters, surveillance practices, and institutional response. This finding suggests that the SEM’s conventional representation of levels as relatively independent layers may need to be adapted for outbreak contexts, where a single social process can permeate and connect all levels simultaneously.

Similarly, the HSDF proved valuable for categorising stigma mechanisms, but our findings suggest areas for extension. The framework’s typology of enacted, perceived, internalised, and associative stigma captured important dimensions of participants’ accounts, yet these mechanisms operated in reinforcing cycles rather than as parallel categories. Enacted stigma fuelled perceived stigma, perceived stigma encouraged concealment, concealment delayed care-seeking and contact disclosure, and delayed disclosure intensified community suspicion and further enacted stigma. This cyclical dynamic, while implied by the framework, deserves more explicit theorisation, particularly in outbreak contexts where the temporal compression of an epidemic may accelerate stigma cycles in ways not fully captured by frameworks developed for chronic conditions such as HIV. Future research might explore how the HSDF could be adapted to account for the rapid onset, acute visibility, sexual and moral associations, and epidemic tempo of mpox-related stigma compared with the more gradual stigma trajectories characteristic of chronic infectious diseases [40].

Beyond the cyclical dynamic, several inductive extensions of the HSDF emerged from the data that were not anticipated by the framework. Associative stigma, conventionally documented toward family members and caregivers, was extended in this study to contact tracers and outbreak response personnel who were stigmatised by association with the

response itself rather than with illness. Institutional settings associated with mpox care— treatment centres and vaccination sites—also carried stigmatising meaning: participants avoided vaccination queues and treatment facilities to prevent public identification with the disease, a form of institutionally located stigma not captured by the framework’s original typology. Religious and moral idioms shaped internalised stigma in ways that extended beyond the framework’s conception of self-stigma; survivors drew on spiritual frameworks of punishment and moral failing to account for their illness, reflecting culturally specific self-attribution processes absent from frameworks developed primarily in secular, high-income contexts. Finally, informal stigma-mitigation practices emerged spontaneously among frontline response teams: contact tracers and healthcare workers developed strategies to reduce the visibility of response activities, adopt neutral language in community settings, and avoid publicly identifying individuals through observable behaviour. These adaptations represent a form of stigma-responsive practice grounded in lived outbreak experience and warrant recognition in response training and preparedness guidance.

### Healthcare workers’ experiences

Healthcare workers’ narratives of moral distress, occupational risk anxiety, and emotional burden align with documented experiences from other outbreaks while revealing mpox- specific dimensions [22,23,24,25,26]. The compound effect of sequential outbreak response was especially salient. Many participants had served during both Ebola and COVID-19 before being asked to respond to mpox, and they described earlier memories, fears, and losses returning during the new response. This pattern is not fully captured by burnout alone. Burnout offers an important comparator because it describes emotional exhaustion, depersonalisation, and reduced personal accomplishment arising from

chronic occupational strain [27,41]. In this study, however, healthcare workers described the reactivation of prior outbreak-related fear, grief, and moral conflict across sequential public health emergencies. We therefore use compound trauma descriptively to refer to the cumulative emotional and professional burden produced when successive epidemic responses reactivate prior exposure before adequate recovery or institutional repair occurs.

This descriptive interpretation is anchored in, but does not attempt to develop, the wider literature on moral distress, moral injury, and cumulative disaster exposure. Moral distress describes knowing the ethically appropriate action while being constrained from acting [42], and the crescendo effect explains how moral residue can accumulate across repeated incidents [43]. Moral injury encompasses long-term psychological, social, moral, and identity-related effects of witnessing, failing to prevent, or participating in events that violate deeply held moral beliefs [44,45]. Disaster literature similarly shows that multiple or successive exposures can deepen mental health effects and delay recovery, particularly when events occur in close temporal proximity [46,47,48]. A living systematic review on health and care workers during COVID-19 and other public health emergencies of international concern likewise highlights mental health, well-being, and quality-of-life impacts as core workforce consequences of emergencies [49]. In Sierra Leone, the relevance of these concepts is heightened by the memory of Ebola, the COVID-19 response, recurrent resource scarcity, and repeated expectations that frontline staff absorb risk on behalf of the public [9,22]. We therefore present compound trauma as an empirical pattern that requires further dedicated conceptual and longitudinal study, rather than as a fully settled diagnostic or theoretical construct.

The implication is that psychosocial support for healthcare workers cannot be treated as an episodic add-on during each outbreak. Settings with recurrent epidemic exposure require continuity of workforce well-being programmes, access to confidential mental health

support, peer support systems, supportive supervision, and institutional recognition of moral distress. We note, however, that healthcare worker accounts in this study primarily reflect the perspectives of those who remained in their posts during the outbreak; the experiences of workers who left clinical practice or were unable to continue responding remain undocumented and may differ substantially.

### Implications for practice and policy

Our findings suggest several implications for mpox response in Sierra Leone and potentially in similar settings, though we note that these are indicative rather than prescriptive, and their applicability will depend on local context and implementation considerations.

First, the pervasive role of stigma across all domains of the outbreak experience suggests that stigma-informed strategies merit integration into outbreak management frameworks from the outset, rather than being treated as secondary concerns [22,40]. This could include attention to language used in public communications, protection of patient confidentiality, community engagement to address misinformation and fear, and support for affected individuals facing discrimination [50]. The specific forms that such strategies might take require further development and evaluation.

Second, accounts from healthcare workers suggest that support mechanisms, including adequate PPE, access to vaccination, psychosocial support, strategies to address associative stigma, and career protections for outbreak responders, should be established as integral components of the response architecture rather than afterthoughts [25,26]. The compound trauma observed among workers with prior epidemic experience further suggests the potential value of continuity in workforce wellbeing programmes across successive outbreaks.

Third, the care-seeking patterns observed in this study suggest that community-centred communication approaches—emphasising symptom recognition and care availability, delivered through trusted community channels—may facilitate timely care-seeking [40,50]. However, our data captures what participants reported about their care-seeking experiences and decisions, and social desirability considerations may have shaped these accounts. Intervention research is needed to evaluate the effectiveness of specific communication strategies.

### Limitations

Several limitations should be acknowledged. First, although the sample was purposively diverse across participant groups and districts, it may not represent all experiences of the outbreak. In particular, people who never accessed healthcare, those who declined participation because of stigma, and those who experienced severe outcomes may be underrepresented. The geographic concentration of cases in the Western Area may also limit the transferability of findings to districts with different epidemiological and social profiles.

Second, the study relied on retrospective self-reported accounts collected during a highly sensitive outbreak. Recall bias may have affected illness narratives, and social desirability bias may have shaped responses across participant groups. Survivors may have understated socially sensitive exposures or behaviours, community members may have moderated stigmatising attitudes, and healthcare workers may have emphasised operational constraints. Sexual exposures and partner-related experiences were also likely underreported because of stigma, fear of disclosure, and the social risks attached to discussing sexual behaviour in this setting. We therefore make no claims about individual transmission routes.

Third, the study's aims and the HSDF-informed sensitising framework directed analytical attention toward stigma-related experiences. This may have increased attention to stigma

compared with other possible dimensions of the outbreak experience. To reduce this risk, we treated the framework as a sensitising tool rather than a rigid coding template, and the specific forms, mechanisms, pathways, and effects of stigma reported here were developed from participant accounts rather than imposed in advance.

Fourth, although individual interviews were used for sensitive survivor accounts and for healthcare workers’ more personal experiences, some healthcare worker FGDs may have been affected by hierarchy and workplace dynamics. Facilitators attempted to mitigate this by monitoring group interactions, inviting dissenting views, and offering follow-up SSIs as needed, but some participants may still have moderated their criticism of colleagues, supervisors, or institutional practices.

Fifth, the cross-sectional nature of data collection means that the study captures experiences at particular points in the outbreak trajectory and recovery process. How stigma, embodiment, care-seeking, and healthcare worker distress evolve over time remains an important question for longitudinal research. The absence of male partner perspectives, additional family members, and individuals who declined healthcare also limits the completeness of our account of the relational dimensions of mpox experience.

Despite these limitations, the multi-perspective design, diverse sample, member checking, framework-based analysis, and triangulation across survivor, healthcare worker, community, and contact tracer accounts support the credibility of the findings within the study's scope.

# Conclusions

Stigma was the dominant social force shaping the mpox outbreak experience in Sierra Leone. It delayed care-seeking, undermined contact tracing, eroded trust in health services, and compounded the emotional burden on healthcare workers, all of which directly compromised outbreak control. Because stigma was anticipated in the analytical framework, the key finding is not simply that stigma existed, but that it operated recursively across individual, interpersonal, community, institutional, and system levels.

Healthcare workers with prior Ebola and COVID-19 experience described a compound trauma pattern that current support systems did not fully address. This should not be read as a settled diagnostic construct; rather, it signals that prior outbreak exposure, unresolved moral distress, and recurrent expectations of risk need to be considered in workforce support planning. Health worker support systems should therefore extend across successive outbreaks rather than being rebuilt for each emergency.

Three priorities emerge for outbreak preparedness in similar post-conflict, resource-constrained settings: (1) stigma-informed strategies integrated from the outset of outbreak response, not appended later; (2) healthcare worker support systems that extend across successive outbreaks rather than being rebuilt each time; and (3) community-centred communication, vaccination, and contact tracing approaches that protect confidentiality and reach people before stigma drives them into silence. These priorities require further evaluation, but the experiences documented here show that technical response alone is insufficient without attention to the social and workforce dimensions of epidemic disease.

# Acknowledgments

We extend our profound gratitude to all study participants who shared their experiences during an extraordinarily difficult time. We thank the National Public Health Agency leadership for institutional support; the District Health Management Teams of Western Area Urban, Western Area Rural, Bo, and Kenema for facilitating fieldwork; the research assistants who conducted data collection with sensitivity and skill; and the transcriptionists and translators who enabled accurate representation of participants’ words. We acknowledge technical input from Partners in Health Sierra Leone for mental health referral support.

# Author contributions

**Conceptualization:** Eric Nzirakaindi Ikoona.

**Data curation:** Eric Nzirakaindi Ikoona, Lucy Namulemo.

**Formal analysis:** Eric Nzirakaindi Ikoona, Lucy Namulemo.

**Investigation:** Eric Nzirakaindi Ikoona, Lucy Namulemo, Mary Magdalene Sinnah.

**Methodology:** Eric Nzirakaindi Ikoona, Mohamed Alex Vandi.

**Project administration:** Eric Nzirakaindi Ikoona.

**Supervision:** Foday Sahr.

**Writing – original draft:** Eric Nzirakaindi Ikoona.

**Writing – review & editing:** Eric Nzirakaindi Ikoona, Lucy Namulemo, Mary Magdalene Sinnah, Mohamed Alex Vandi, Foday Sahr.

# Funding

The authors received no specific funding for this work.

# Competing interests

The authors have declared that no competing interests exist.

# Data availability statement

De-identified qualitative data (full transcripts) cannot be shared publicly because they contain sensitive information and carry a risk of participant re-identification, given the small number of mpox cases in specific geographic areas during the study period. Study materials supporting transparency and reproducibility are available in the Supporting Information files: interview and focus group discussion guides (S2-S3 Appendices), the full coding framework (S4 Appendix), the completed COREQ checklist (S5 Appendix), extended de-identified quotations mapped to themes (S6 Appendix), and the completed PLOS Inclusivity in Global Research Questionnaire (S7 Appendix). Researchers may request access to further de-identified data excerpts under a data-sharing agreement. Requests should be directed to the institutional data steward, Dr. Mohamed Boie Jalloh, Director of Planning, Policy and Research, National Public Health Agency (email: info@npha.gov.sl; tel. +23277121212), along with a brief proposal describing the intended use, data protection safeguards, and an ethics-compliant data management plan. Access is subject to approval by the Sierra Leone Ethics and Scientific Review Committee, consistent with the ethics clearance for this study (SLESRC-2025-049). Data will be retained by the National Public Health Agency for a minimum of five years following study completion.

# References

1. World Health Organization. 2024. WHO Director-General declares mpox outbreak a public health emergency of international concern. Aug 14 [cited 2026 Jan 26]. Available from: [https://www.who.int/news/item/14-08-2024-who-director-general-declares-mpox-](https://www.who.int/news/item/14-08-2024-who-director-general-declares-mpox-outbreak-a-public-health-emergency-of-international-concern)

[outbreak-a-public-health-emergency-of-international-concern](https://www.who.int/news/item/14-08-2024-who-director-general-declares-mpox-outbreak-a-public-health-emergency-of-international-concern)

2. Ministry of Health (Sierra Leone). 2025. Mpox declared a public health emergency in Sierra Leone. Jan 16 [cited 2026 Jan 26]. Available from: [https://mohs.gov.sl/mpox-](https://mohs.gov.sl/mpox-declared-a-public-health-emergency-in-sierra-leone/)

[declared-a-public-health-emergency-in-sierra-leone/](https://mohs.gov.sl/mpox-declared-a-public-health-emergency-in-sierra-leone/)

3. UNICEF Sierra Leone. 2025. Fighting Mpox: UNICEF scales up critical support in Sierra Leone. Jun 18 [cited 2026 Jan 26]. Available from: [https://www.unicef.org/sierraleone/press-releases/fighting-mpox-unicef-scales-critical-](https://www.unicef.org/sierraleone/press-releases/fighting-mpox-unicef-scales-critical-support-sierra-leone)

[support-sierra-leone](https://www.unicef.org/sierraleone/press-releases/fighting-mpox-unicef-scales-critical-support-sierra-leone)

4. World Health Organization. 2025. Multi-country outbreak of mpox, external situation report #54 - 27 June 2025. Jun 27 [cited 2026 Jan 26]. Available from: [https://www.who.int/publications/m/item/multi-country-outbreak-of-mpox--external-](https://www.who.int/publications/m/item/multi-country-outbreak-of-mpox--external-situation-report--54---27-june-2025)

[situation-report--54	27-june-2025](https://www.who.int/publications/m/item/multi-country-outbreak-of-mpox--external-situation-report--54---27-june-2025)

5. Africa Centres for Disease Control and Prevention. 2025. Sierra Leone fights mpox.

May 18 [cited 2026 Jan 26]. Available from: [https://africacdc.org/news-item/sierra-leone-](https://africacdc.org/news-item/sierra-leone-fights-mpox/)

[fights-mpox/](https://africacdc.org/news-item/sierra-leone-fights-mpox/)

6. Sandi JD, Grant DS, Sahr F, Harding A. 2025. Genomic epidemiology of Mpox virus in Sierra Leone. Virological [Internet]. May 28 [cited 2026 Jan 26]. Available from: <https://virological.org/t/genomic-epidemiology-of-mpox-virus-in-sierra-leone/995>
7. Kangbai JB, Saidu E, Foday IK, et al. 2025. Clinical and epidemiological characteristics among probable and confirmed patients with mpox in Sierra Leone reported from January to May 2025. medRxiv [Preprint] [cited 2025 Aug 30]. doi:10.1101/2025.05.30.25328691
8. Mathiang JR, Sahr F, Kamara IF, Ansumana R, Rogers MK, Lee SS, et al. 2025. Rapid

surge of mpox in Sierra Leone: public health challenges. Int J Infect Dis. 159:107987.

doi:10.1016/j.ijid.2025.107987

9. Raven J, Wurie H, Witter S. 2018. Health workers’ experiences of coping with the Ebola epidemic in Sierra Leone’s health system: a qualitative study. BMC Health Serv Res. 18:251. doi:10.1186/s12913-018-3072-3
10. Bah AJ, James PB, Bah N, et al. 2020. Prevalence of anxiety, depression, and post- traumatic stress disorder among Ebola survivors in northern Sierra Leone: a cross- sectional study. BMC Public Health. 20:1391. doi:10.1186/s12889-020-09507-6
11. Médecins Sans Frontières. 2025. Responding to an mpox outbreak in the heart of Sierra Leone. Jul 8 [cited 2025 Aug 30]. Available from: [https://www.msf.org/responding-mpox-](https://www.msf.org/responding-mpox-outbreak-heart-sierra-leone)

[outbreak-heart-sierra-leone](https://www.msf.org/responding-mpox-outbreak-heart-sierra-leone)

12. Fischer LS, Mansergh G, Lynch J, Santibanez S. 2019. Addressing disease-related stigma during infectious disease outbreaks. Disaster Med Public Health Prep. 13:989-994. doi:10.1017/dmp.2018.157
13. Paterson A, Olliaro P, Rojek A. 2023. Addressing stigma in infectious disease outbreaks: a crucial step in pandemic preparedness. Front Public Health. 11:1303679. doi:10.3389/fpubh.2023.1303679
14. Link BG, Phelan JC. 2001. Conceptualizing stigma. Annu Rev Sociol. 27:363-385. doi:10.1146/annurev.soc.27.1.363
15. Stangl AL, Earnshaw VA, Logie CH, et al. 2019. The health stigma and discrimination framework: a global, crosscutting framework to inform research, intervention development, and policy on health-related stigmas. BMC Med. 17:31. doi:10.1186/s12916-019-1271-3
16. Sah R, Mohanty A, Reda A, Padhi BK, Rodriguez-Morales AJ. 2022. Stigma during monkeypox outbreak. Front Public Health. 10:1023519. doi:10.3389/fpubh.2022.1023519
17. Orsini D, Sartini M, Spagnolo AM, Cristina ML, Martini M. 2024. Mpox: ‘the stigma

is as dangerous as the virus.’ Historical, social, ethical issues and future forthcoming. J

Prev Med Hyg. 64(4):E398-E404. doi:10.15167/2421-4248/jpmh2023.64.4.3144

18. Smith AKJ, Storer D, Lancaster K, et al. 2024. Mpox illness narratives: stigmatising care and recovery during and after an emergency outbreak. Qual Health Res. 34(12):1161- 1174. doi:10.1177/10497323241234482
19. Takenaka BP, Kirklewski SJ, Griffith FJ, et al. 2024. ‘It’s another gay disease’: an intersectional qualitative approach contextualizing the lived experiences of young gay, bisexual, and other sexual minoritized men in the United States during the mpox outbreak. BMC Public Health. 24:1574. doi:10.1186/s12889-024-19062-z
20. Witzel TC, Ghobrial A, Palich R, et al. 2024. Experiences of mpox illness and case management among cis and trans gay, bisexual and other men who have sex with men in England: a qualitative study. EClinicalMedicine. 70:102522. doi:10.1016/j.eclinm.2024.102522
21. Biesty CP, Hemingway C, Woolgar J, et al. 2024. Community led health promotion to counter stigma and increase trust amongst priority populations: lessons from the 2022- 2023 UK mpox outbreak. BMC Public Health. 24:1638. doi:10.1186/s12889-024-19142- 2
22. Logie CH. 2022. What can we learn from HIV, COVID-19 and mpox stigma to guide stigma-informed pandemic preparedness? J Int AIDS Soc. 25:e26060. doi:10.1002/jia2.26060
23. Billings J, Ching BCF, Gkofa V, et al. 2021. Experiences of frontline healthcare workers and their views about support during COVID-19 and previous pandemics: a systematic review and qualitative meta-synthesis. BMC Health Serv Res. 21(1):923. doi:10.1186/s12913-021-06917-z
24. Apea V, Titanji BK, Dakin FH, et al. 2025. International healthcare workers’ experiences and perceptions of the 2022 multi-country mpox outbreak. PLOS Glob Public

Health. 5(2):e0003704. doi:10.1371/journal.pgph.0003704

25. Bow SMA, Schröder-Bäck P, Norcliffe-Brown D, et al. 2023. Moral distress and injury in the public health professional workforce during the COVID-19 pandemic. J Public Health (Oxf). 45(3):697-705. doi:10.1093/pubmed/fdad010
26. Jahromi AS, Jokar M, Sharifi N, et al. 2024. Global knowledge and attitudes towards mpox (monkeypox) among healthcare workers: a systematic review and meta-analysis. Int Health. 16:487-498. doi:10.1093/inthealth/ihad107
27. West CP, Dyrbye LN, Sinsky C, et al. 2020. Resilience and burnout among physicians and the general US working population. JAMA Netw Open. 3(7):e209385. doi:10.1001/jamanetworkopen.2020.9385
28. Hussein M, et al. 2024. Ethical considerations during mpox outbreak: a scoping review.

BMC Med Ethics. 25:78. doi:10.1186/s12910-024-01080-4

29. May T, Towler L, Smith LE, et al. 2023. Mpox knowledge, behaviours and barriers to public health measures among gay, bisexual and other men who have sex with men in the UK: a qualitative study to inform public health guidance and messaging. BMC Public Health. 23(1):2265. doi:10.1186/s12889-023-17134-4
30. Torres TS, Silva MST, Coutinho C, et al. 2023. Evaluation of mpox knowledge, stigma, and willingness to vaccinate for mpox: cross-sectional web-based survey among sexual and gender minorities. JMIR Public Health Surveill. 9:e46489. doi:10.2196/46489
31. McLeroy KR, Bibeau D, Steckler A, Glanz K. 1988. An ecological perspective on health promotion programs. Health Educ Q. 15(4):351-377. doi:10.1177/109019818801500401
32. Gale NK, Heath G, Cameron E, Rashid S, Redwood S. 2013. Using the framework method for the analysis of qualitative data in multi-disciplinary health research. BMC Med Res Methodol. 13:117. doi:10.1186/1471-2288-13-117
33. Ritchie J, Spencer L. 1994. Qualitative data analysis for applied policy research. In:

Bryman A, Burgess RG, editors. Analyzing qualitative data. London: Routledge; p. 173-

194.

34. Pope C, Ziebland S, Mays N. 2000. Qualitative research in health care: analysing qualitative data. BMJ. 320(7227):114-116. doi:10.1136/bmj.320.7227.114
35. May CR, Johnson M, Finch T. 2016. Implementation, context and complexity.

Implement Sci. 11:141. doi:10.1186/s13012-016-0506-3

36. Tong A, Sainsbury P, Craig J. 2007. Consolidated criteria for reporting qualitative research (COREQ): a 32-item checklist for interviews and focus groups. Int J Qual Health Care. 19:349-357. doi:10.1093/intqhc/mzm042
37. Schmalzle SA, Grant M, Lovelace S, et al. 2024. Survey of pain and stigma experiences in people diagnosed with mpox in Baltimore, Maryland during 2022 global outbreak. PLoS ONE. 19(5):e0299587. doi:10.1371/journal.pone.0299587
38. Bury M. 1982. Chronic illness as biographical disruption. Sociol Health Illn. 4:167-

182. doi:10.1111/1467-9566.ep11339939

39. Hatzenbuehler ML, Phelan JC, Link BG. 2013. Stigma as a fundamental cause of population health inequalities. Am J Public Health. 103:813-821. doi:10.2105/AJPH.2012.301069
40. Paterson A, Cheyne A, Jones B, et al. 2025. An hourglass model for conceptualising stigma in infectious disease outbreaks. Sci Rep. 15:15339. doi:10.1038/s41598-025- 98995-w
41. Maslach C, Jackson SE. 1981. The measurement of experienced burnout. J Occup Behav. 2(2):99-113. doi:10.1002/job.4030020205
42. Jameton A. 1984. Nursing practice: The ethical issues. Englewood Cliffs (NJ): Prentice-Hall.
43. Epstein EG, Hamric AB. 2009. Moral distress, moral residue, and the crescendo effect.

J Clin Ethics. 20(4):330-342.

44. Litz BT, Stein N, Delaney E, Lebowitz L, Nash WP, Silva C, et al. 2009. Moral injury

and moral repair in war veterans: a preliminary model and intervention strategy. Clin Psychol Rev. 29(8):695-706. doi:10.1016/j.cpr.2009.07.003

45. Dean W, Talbot SG, Dean A. 2019. Reframing clinician distress: moral injury not burnout. Fed Pract. 36(9):400-402.
46. Leppold C, Gibbs L, Block K, Reifels L, Quinn P. 2022. Public health implications of multiple disaster exposures. Lancet Public Health. 7(3):e274-e286. doi:10.1016/S2468- 2667(21)00255-3
47. Newnham EA, Mergelsberg ELP, Chen Y, Kim Y, Gibbs L, Dzidic PL, et al. 2022.

Long term mental health trajectories after disasters and pandemics: a multilingual systematic review of prevalence, risk and protective factors. Clin Psychol Rev. 97:102203. doi:10.1016/j.cpr.2022.102203

48. Li A, Leppold C. 2025. Long-term mental health trajectories across multiple exposures to climate disasters in Australia: a population-based cohort study. Lancet Public Health. 10(5):e391-e400. doi:10.1016/S2468-2667(25)00068-4
49. Fronteira I, Mathews V, Bandeira Dos Santos RL, Matsumoto K, Amde W, Pereira A, et al. 2024. Impacts for health and care workers of COVID-19 and other public health emergencies of international concern: living systematic review, meta-analysis and policy recommendations. Hum Resour Health. 22(1):10. doi:10.1186/s12960-024-00892-2
50. Betsch C, Wieler LH, Habersaat K. 2020. Monitoring behavioural insights related to COVID-19. Lancet. 395(10232):1255-1256. doi:10.1016/S0140-6736(20)30729-7

# Supporting information

**S1 Appendix. Framework description and thematic framework summary.** Integrated SEM+HSDF analytical framework, S1 Fig thematic framework diagram, and thematic framework overview.

**S2 Appendix. Semi-structured interview guides.** Interview guides for survivors,

healthcare workers, community members, and contact tracers.

**S3 Appendix.** Focus group discussion guide. Guide for focus group discussions with healthcare workers, community members, and contact tracers.

**S4 Appendix.** Coding framework. Full codebook with code names, definitions, inclusion/exclusion criteria, and example excerpts.

**S5 Appendix.** COREQ checklist. Completed the Consolidated Criteria for Reporting Qualitative Research checklist.

**S6 Appendix.** Extended participant quotations by theme. Additional quotations supporting each theme and subtheme.

**S7 Appendix.** PLOS Inclusivity in Global Research Questionnaire. Completed questionnaire describing local leadership, Sierra Leonean authorship, community and institutional embeddedness, and the contribution of regional qualitative and mental health expertise.

**S1 Fig.** Thematic framework showing five overarching themes and their interconnections. The full thematic framework diagram, including the five overarching themes, their associated subthemes, and the theoretical grounding within the modified Social Ecological Model and the Health Stigma and Discrimination Framework, is presented in this figure. The complete framework description and explanatory text are provided in S1 Appendix.

S1 Fig. Thematic framework diagram

[Click here to access/download](https://www2.cloud.editorialmanager.com/pgph/download.aspx?id=795925&guid=607ae92d-395f-4844-b325-ed93e7a6e835&scheme=1)

[**Supporting Information** Figure1.tif](https://www2.cloud.editorialmanager.com/pgph/download.aspx?id=795925&guid=607ae92d-395f-4844-b325-ed93e7a6e835&scheme=1)

Supplementary Documents

## [Click here to access/download](https://www2.cloud.editorialmanager.com/pgph/download.aspx?id=795926&guid=dcd1683c-deae-4bb2-bcbb-5f2accda56d5&scheme=1)

[**Supporting Information** mpox_PLOS_supplementary_comprehensive.docx](https://www2.cloud.editorialmanager.com/pgph/download.aspx?id=795926&guid=dcd1683c-deae-4bb2-bcbb-5f2accda56d5&scheme=1)

Inclusivity Questionnaire

## [Click here to access/download](https://www2.cloud.editorialmanager.com/pgph/download.aspx?id=795927&guid=abbeaec4-4f07-43d9-9432-4a3607c7a1b0&scheme=1)

[**Supporting Information**](https://www2.cloud.editorialmanager.com/pgph/download.aspx?id=795927&guid=abbeaec4-4f07-43d9-9432-4a3607c7a1b0&scheme=1)

[PGPH-D-26-00336_S7_Inclusivity_Questionnaire.docx](https://www2.cloud.editorialmanager.com/pgph/download.aspx?id=795927&guid=abbeaec4-4f07-43d9-9432-4a3607c7a1b0&scheme=1)

Revised Manuscript with Track Changes

**Suffering in silence: Stigma, healthcare barriers, and resilience during Sierra Leone’s 2025 clade IIb mpox ~~outbreak: A~~outbreak—A multi- perspective qualitative study**

**Short Title: Mpox stigma and healthcare experiences in Sierra Leone**

Eric Nzirakaindi Ikoona¹*, Lucy Namulemo²·³, Mary Magdalene Sinnah¹, Mohamed Alex Vandi¹, Foday Sahr¹

¹ National Public Health Agency, Freetown, Sierra Leone

² Foothills Community-Based Interventions, Monticello, Kentucky, United States of America

³ Uganda Counselling and Support Services, Kampala, Uganda

* Corresponding author

E-mail: [ikoonae@yahoo.com](mailto:ikoonae@yahoo.com) (ENI)

**Abstract**

**Background**

Mpox was confirmed in Sierra Leone in January 2025, with a public health emergency declared shortly thereafter. By June 2025, the country reported over 4,000 confirmed cases caused by clade IIb. Limited qualitative research has explored the lived experiences of affected populations during mpox outbreaks in African settings. This study explored multi- stakeholder experiences of mpox, including illness trajectories, stigma manifestations, healthcare-seeking behaviours, and health system response challenges.

**Formatted:** List Paragraph, Left, None, Indent: Left: 0", First line: 0", Right: 0", Line spacing: single, Widow/Orphan control, Adjust space between Latin and Asian text, Adjust space between Asian text and numbers

**Formatted:** Not Highlight

**Methods**

We conducted a multi-perspective qualitative study using thematic framework analysis between March and August 2025 across four districts. Participants included mpox survivors (n=42), healthcare workers (n=38), community members (n=36), and contact tracers (n=24). Data were collected through 94 semi-structured interviews and 12 focus group discussions. Analysis was guided by a modified Social Ecological Model (SEM)

integrating the Health Stigma and Discrimination Framework (HSDF).

**Results**

Five interconnected themes emerged: (1) illness characterised by physical suffering and lasting bodily changes; (2) multi-layered stigma operating across individual, interpersonal, community, and institutional levels; (3) healthcare-seeking shaped by fear of disclosure and economic constraints; (4) healthcare workers’ moral distress and professional isolation; (5) adaptive health system response alongside coordination challenges. Stigma operated as a cross-cutting dimension that intersected with all other themes, shaping illness experience, care-seeking behaviour, professional practice, and system-level response simultaneously.

**Conclusions**

Stigma permeated all dimensions of the mpox outbreak experience, delaying care- seeking, undermining contact tracing, and compounding healthcare worker distress. Compound trauma from sequential epidemic exposure emerged as a distinct burden among frontline workers. These findings point to the need for stigma-informed outbreak response strategies, sustained healthcare worker support mechanisms, and community-

centred communication approaches in post-conflict, resource-constrained settings facing recurrent epidemics.

**Keywords: mpox, monkeypox, Sierra Leone, qualitative research, lived experience, stigma, healthcare workers, health systems, outbreak response, clade IIb**

**Introduction**

Mpox (formerly monkeypox) remains a significant public health concern. The World Health Organization (WHO) declared the mpox upsurge a Public Health Emergency of International Concern (PHEIC) on 14 August 2024 [~~4~~1]. Mpox was first confirmed in

Sierra Leone on 10 January 2025, the outbreak was officially declared on 13 January 2025, and a national public health emergency was declared on 16 January 2025 [~~1~~2,~~2~~3]. By 22

June 2025, the country reported 4,294 confirmed cases and 28 deaths [~~3~~4]. In early May

2025, Sierra Leone accounted for approximately half of Africa’s confirmed mpox cases, underscoring the intensity of transmission during the outbreak acceleration phase [5].

The Sierra Leone outbreak presents distinctive epidemiological and social characteristics. Genomic evidence indicates that most sequences fall within clade IIb and cluster as a distinct group descended from lineage A.2.2, consistent with sustained human-to-human transmission; a proposed designation identifies this newly observed Sierra Leone lineage as G.1 (alias of A.2.2.1) [6]. Frequent genital involvement among cases supports the plausibility of sexual networks contributing to clade IIb transmission in this setting [7,8].

Sexual exposure histories are socially and legally sensitive, ~~carrying~~and self-reported

accounts of exposure have raised the possibility of underreporting, stigma, and disclosure

constraints [8]. These epidemiological and social features carry significant implications

for understanding disease spread, designing targeted interventions, and addressing the complex social dimensions of the outbreak.

**The Sierra Leone context**

Sierra Leone’s mpox response occurs within a health system shaped by post-conflict rebuilding and the 2014–2016 Ebola epidemic, which disrupted services and workforce capacity [9,10]. More recently, the country navigated the COVID-19 pandemic while simultaneously addressing endemic challenges, including malaria, tuberculosis, and HIV

[8]. These cumulative experiences have shaped both the health system’s adaptive capacity and community perceptions of outbreak response, creating a distinctive context in which trust, institutional memory, and resource constraints interact in ways that may differ from settings without comparable histories of sequential health emergencies.

The concentration of cases in the Western Area, encompassing Freetown and surrounding districts, reflects underlying patterns of urbanisation, population density, and healthcare access [34]. By mid-

2025, four dedicated treatment centres had opened, a 400-bed facility ~~became~~had become

operational in Freetown, and over 43,000 individuals had received ~~vaccination—yet~~vaccination—

yet these resources remained insufficient for the scale of the epidemic [34,11].

**Stigma and infectious disease outbreaks**

Infectious disease outbreaks have historically been accompanied by stigmatisation of affected individuals and communities [12,13~~,14~~]. Link and Phelan’s foundational

conceptualisation describes stigma as occurring when elements of labelling, stereotyping, separation, status loss, and discrimination converge ~~in~~within a context of power imbalance

[~~15~~14]. Disease-related stigma manifests through multiple mechanisms: enacted stigma

(actual experiences of discrimination), perceived stigma (expectations of mistreatment), ~~internalised stigma (acceptance of negative societal attitudes), and associative stigma~~

~~(stigma experienced by those connected to affected individuals) [16].~~

internalised stigma (acceptance of negative societal attitudes), and associative stigma

(stigma experienced by those connected to affected individuals) [15].

The mpox outbreak presents particular stigma challenges given its visible cutaneous manifestations, perceived associations with sexual transmission, and historical framings as an “African disease” [16,17~~,18~~]. Evidence from the 2022 global outbreak documented

significant stigma among affected populations, including personalised stigma, negative self-image, and disclosure concerns [18,19~~,20~~]. Healthcare workers have similarly

reported fear of contagion and social isolation [20,21~~,22~~]. While these dynamics are well

documented in high-income country contexts, significant gaps remain in understanding how mpox- related stigma operates in African settings, where different cultural meanings,

health system structures, and prior outbreak experiences may produce distinct stigma configurations [~~23~~22].

**Healthcare ~~worker~~workers' experiences in outbreak response**

Healthcare workers occupy a critical yet vulnerable position during infectious disease outbreaks [~~24~~23]. Research from the 2022 mpox response documented knowledge gaps,

with 30% of healthcare workers having never heard of mpox before the outbreak and over one- quarter initially misdiagnosing cases [~~25~~24]. Healthcare workers reported inadequate

access to vaccines, concerns about occupational exposure, and moral distress related to resource constraints [~~26~~25]. The psychological toll of outbreak response—compounded

for many by the recent COVID-19 pandemic—has contributed to burnout, compassion fatigue, and professional attrition [26,27~~,28~~].

**Gaps in current knowledge**

Despite growing epidemiological surveillance of mpox in Africa, qualitative research examining lived experiences remains markedly limited [28,29~~,30~~]. To our knowledge, no

published qualitative studies have examined mpox experiences from endemic African settings during the current PHEIC period. Existing qualitative research has predominantly emerged from high-income country contexts, primarily focusing on men who have sex with men populations affected by clade IIb during the 2022 outbreak [18,19,~~20,31~~30]. This

leaves significant knowledge gaps regarding how mpox illness is experienced in African contexts, the specific manifestations and mechanisms of mpox-related stigma in these settings, and healthcare-seeking behaviours and barriers in resource-constrained environments.

**Study aims and theoretical framework**

This study aimed to provide a comprehensive qualitative exploration of mpox outbreak experiences in Sierra Leone, addressing the following research questions: (1) How do individuals who have experienced mpox understand and narrate their illness trajectories?

(2) What forms and mechanisms of stigma emerge during mpox outbreaks, and how do

these affect individuals, families, and communities? (3) What factors shape healthcare- seeking behaviours among those affected by or at risk of mpox? (4) How do healthcare workers and public health personnel experience outbreak response? (5) How do experiences vary across different social positions and geographic contexts?

Our analysis was guided by a modified Social Ecological Model (SEM) integrating the

Health Stigma and Discrimination Framework (HSDF) [15,31] (S1 Appendix). The SEM

~~Our analysis was guided by a modified Social Ecological Model (SEM) integrating the~~

~~Health Stigma and Discrimination Framework (HSDF) [16,32] (S1 Appendix). The SEM~~

provides a multi-level structure for examining individual, interpersonal, community, organisational, and policy factors ~~shaping~~that shape health experiences. The HSDF offers

a cross- cutting framework for understanding how stigma operates through marking,

labelling, separation, status loss, and discrimination within specific contexts. We employed these frameworks as sensitising tools rather than ~~as~~ rigid analytical templates,

remaining open to findings that might challenge, extend, or complicate the frameworks’ ~~assumptions—particularly~~assumptions— particularly regarding the interplay between

structural constraints and individual-level processes in a post-conflict, resource- constrained setting.

**Methods**

**Ethics Statement**

Ethical approval was obtained from the Sierra Leone Ethics and Scientific Review

Committee (SLESRC-2025-049) on 28 February 2025. All participants provided written

informed consent before participation. For participants who could not write, witnessed

thumbprint consent was obtained in accordance with SLESRC guidance. The consent

process covered the study purpose, procedures, voluntary participation, right to withdraw,

confidentiality protections, potential emotional risks, referral options, audio-recording,

data storage, dissemination plans, and contact information for the research team and

ethics committee.

Given the potentially stigmatising nature of mpox, confidentiality protections were

central to the study design. Participants selected interview locations that offered privacy;

identifiers were removed during transcription; quotations are presented using

pseudonyms and with identifying details altered; and data files are stored on encrypted,

password-protected servers accessible only to the research team. Emotional distress

protocols were established, and research assistants were trained to recognise distress

signals and offer supportive responses. Referral pathways to mental health services were

established in each district through a partnership with Partners in Health Sierra Leone.

Participants received transportation reimbursement (NLE 50, approximately USD 2.50 at

the June 2025 exchange rate) and refreshments, but no other compensation, to minimise

coercion.

**Study design and period**

We conducted a multi-perspective qualitative study using thematic framework analysis to examine lived experiences of the mpox outbreak [32,33~~,34~~]. The study was conducted

between 10 March and 28 August 2025, spanning the outbreak’s acceleration, peak (May– June 2025), and initial decline phases. Thematic framework analysis is well-suited for policy-relevant health research involving multiple participant groups, enabling systematic comparison across cases while maintaining analytic transparency [~~33~~32,~~35~~34]. Our

analytical approach was informed by the ~~Social Ecological Model~~SEM and the ~~Health~~

~~Stigma and Discrimination Framework~~HSDF, which provided sensitising concepts for

theme development [~~16~~15,~~32~~31].

We incorporated multiple stakeholder perspectives to triangulate and develop a comprehensive understanding of the outbreak experience. This multi-perspective approach aligns with calls for qualitative research that moves beyond single stakeholder

views to capture the relational and systemic dimensions of health phenomena [~~36~~35]. The

four participant groups—survivors, healthcare workers, community members, and contact tracers—occupy distinct epistemic positions. Survivors provide experiential knowledge grounded in embodied illness experience; healthcare workers contribute professional and observational accounts shaped by clinical training and institutional roles; community members offer perspectives on social responses and collective meanings; and contact tracers provide operational insights from the interface between surveillance systems and community engagement. We treated these perspectives as complementary but epistemically distinct, and our analytical approach was designed to differentiate between lived experience and professional or observational discourse (see Data analysis). The study followed the Consolidated Criteria for Reporting Qualitative Research (COREQ) guidelines [~~37~~36] (S5 Appendix).

**Setting**

The study was conducted across four districts representing diverse epidemiological and geographic contexts: Western Area Urban (Freetown), Western Area Rural, Bo, and Kenema. These districts were selected based on: (1) case burden, with Western Area accounting for approximately 80% of confirmed cases; (2) geographic diversity, including urban, peri-urban, and rural settings; (3) healthcare infrastructure variation, from tertiary facilities to community health centres; and (4) prior outbreak experience, with all districts having been affected by the 2014–2016 Ebola epidemic [9].

At the time of data collection, the four districts exhibited distinct epidemiological profiles. Western Area Urban reported the highest case concentration, driven by population density and social mixing patterns characteristic of the capital. Western Area

Rural, while geographically proximate, ~~presented~~exhibited different transmission

dynamics associated with peri-urban settlement patterns and more limited healthcare access. Bo and Kenema districts in the south and east reported lower overall case counts but faced particular challenges related to diagnostic capacity, delayed specimen transport, and greater distances to treatment centres. Across all four districts, cases were predominantly among young adults aged 15–35 years, with male predominance in reported cases [7].

**Participants and sampling**

We recruited 140 participants across four stakeholder groups: mpox survivors who had completed isolation (n=42), healthcare workers involved in mpox response (n=38), community members from affected areas (n=36), and contact tracers/surveillance officers (n=24). Purposive sampling with maximum variation was employed to ensure diversity across demographic characteristics (age, gender, education), geographic location (urban/peri-urban/rural), and for survivors, illness severity and time since recovery.

Inclusion criteria for survivors required: confirmed mpox diagnosis (laboratory or clinical), completion of isolation period, age 18 years or older, capacity to provide informed consent, and willingness to discuss experiences. Healthcare worker eligibility required direct involvement in mpox clinical care, laboratory services, or infection prevention and control. Community member eligibility required residence in a community with documented mpox cases. Contact tracers required current or recent employment in mpox surveillance activities.

**Participant recruitment and contact**

Recruitment utilised multiple pathways tailored to each stakeholder group. For survivor participants, treatment centre staff at the four designated mpox treatment facilities (Connaught Hospital, 34 Military Hospital, Kenema Government Hospital, and Bo Government Hospital) provided study information sheets to patients nearing discharge. Patients who expressed interest were referred to research assistants, who contacted them via telephone 2–4 weeks post-discharge to confirm eligibility and schedule interviews. Community health workers also identified survivors in their catchment areas who had completed home-based isolation.

Healthcare workers were recruited through facility-based approaches. Research team members visited hospitals, peripheral health units, and laboratories involved in the mpox

response to present the study at staff meetings. Interested staff provided contact information and were subsequently contacted to arrange interviews at convenient times. Contact tracers were recruited through District Health Management Teams (DHMTs); surveillance focal persons distributed study information during routine team meetings, and interested personnel were contacted directly by research assistants.

Community members were identified through community leaders and existing community health structures. Community Health Officers introduced the study at community meetings in areas with documented mpox cases. Interested community members provided contact details and were subsequently contacted to arrange participation. We assessed theme stability iteratively within each stakeholder group and district; recruitment stopped when additional interviews did not generate new codes or meaningfully extend existing themes.

**Data collection**

Data collection employed semi-structured interviews (SSIs) and focus group discussions (FGDs), with methods matched to participant group, preferences ~~and~~, topic sensitivity, and

feasibility. Individual SSIs were preferred for survivors ~~given the personal and potentially~~

~~stigmatising nature of~~because illness narratives, perceived sexual exposure, stigma, and

family or workplace disclosure were highly sensitive. For healthcare workers, SSIs were

used to elicit private accounts of moral distress, resource constraints, occupational risk,

and emotional burden, while FGDs were used to explore ~~community~~shared

~~perspectives~~operational experiences, collective reflections, and ~~healthcare~~points ~~worker~~of

~~experiences~~convergence ~~where~~or ~~group~~disagreement ~~dynamics~~across ~~could~~professional

~~generate richer discussion~~roles.

In total, we conducted 94 SSIs (42 with survivors, 28 with healthcare workers, 12 with community members, 12 with contact tracers) and 12 FGDs (4 with healthcare workers, 6 with community members, 2 with contact tracers). FGDs comprised 6–10 participants each, grouped by stakeholder type and district to enable comfortable discussion among peers with shared experiences. For healthcare worker FGDs, we attempted to group

participants by cadre or level of seniority where feasible, but full stratification was not

always possible because of small numbers of eligible staff at some facilities, particularly

in Bo and Kenema.

Because FGDs in clinical settings can suppress disclosure, facilitators monitored group

dynamics, invited quieter participants to speak, reminded participants that disagreement

was acceptable, and avoided questions that required disclosure of identifiable colleague or

patient information. Where participants appeared uncomfortable or where sensitive issues

required more private exploration, follow-up SSIs were offered. We therefore interpret

healthcare worker FGDs as collective professional discourse and place greater evidentiary

weight on SSIs for sensitive individual experiences.

Interview guides were developed separately for each stakeholder group, informed by the theoretical framework and existing literature (S2 and S3 Appendices). Survivor guides explored illness onset and trajectory, healthcare-seeking experiences, social responses and stigma, coping strategies, and perspectives on recovery. Healthcare worker guides examined clinical experiences, occupational challenges, resource constraints, emotional impacts, and recommendations. Community member guides explored knowledge and perceptions, community responses, fears and concerns, and recommendations. Contact tracer guides examined work experiences, challenges encountered, community responses, and support needs.

All guides were piloted with five individuals from each stakeholder group and refined based on feedback. Interviews were conducted in Krio, Mende, Temne, or English according to participant preference by trained Sierra Leonean research assistants fluent

in ~~these~~the local languages used for the study. The research assistants were embedded in

Sierra Leonean cultural contexts and explained study procedures and sensitive concepts

in locally meaningful terms when needed. Interviews averaged 62 minutes for survivors

(range: 45–95 minutes), 48 minutes for other SSIs (range: 35–75 minutes), and 87 minutes for FGDs (range: 72–110 minutes). All sessions were audio-recorded with permission and transcribed verbatim in the original language before ~~translation~~being

~~to~~translated into English by bilingual team members. ~~Translations were verified through~~

~~back-translation of randomly selected segments (20% of transcripts).~~

Translations were verified by back-translating randomly selected segments (20% of

transcripts).

**Informed consent process**

The informed consent process was designed to ensure voluntary, informed participation while being sensitive to the stigmatising nature of mpox. Prior to each interview or FGD, research assistants met with potential participants in a private setting of their choosing (typically their home, a private room at a health facility, or a community centre). ~~Research~~

~~assistants explained the study using a standardised information sheet available in English,~~

~~Krio, Mende, and Temne.~~

Research assistants explained the study using a standardised information sheet available

in English, Krio, Mende, and Temne.

The consent process covered: (1) study purpose and procedures, including the expected duration and topics to be discussed; (2) voluntary nature of participation, with explicit emphasis that declining would not affect access to healthcare or other services; (3) right to withdraw at any time without penalty; (4) right to skip any question or stop the interview at any time; (5) confidentiality protections, including use of pseudonyms, secure data storage, and removal of identifying information; (6) potential risks including emotional distress when discussing difficult experiences; (7) available support including referral to mental health services if needed; (8) audio-recording procedures and data storage; (9) plans for data use and dissemination; and (10) contact information for the research team and ethics committee for questions or concerns.

Participants were given time to ask questions and were provided with the information sheet to keep. For participants who could not read, the information sheet was read aloud in their preferred language. Written informed consent was obtained from all participants before data collection commenced. For participants who could not write, witnessed thumbprint consent was obtained in accordance with Sierra Leone Ethics and Scientific Review Committee guidelines. A copy of the signed consent form was provided to each participant.

**Research team and reflexivity**

**Formatted:** Font: 13 pt, Bold, Not Highlight, Ligatures: None

The research team comprised investigators with backgrounds in public health, medicine, nursing, medical anthropology, and community health. The study was locally led from within Sierra Leone's public health system and institutionally anchored in the National

**Formatted:** Not Highlight

**Formatted:** Space Before: 12.3 pt

Public Health Agency (NPHA), the national institution responsible for public health

protection, surveillance, outbreak preparedness and response, and evidence generation to

understand drivers of public health threats. The principal investigator (ENI) and co-

principal investigator (FS) are physicians and public health researchers based in Sierra

Leone with extensive experience in qualitative health research and implementation

science. The co-investigators MAV and MMS are Sierra Leonean public health

professionals with direct experience in Sierra Leone’s outbreak response architecture,

surveillance systems, and community-facing public health practice. MAV is a physician

and senior public health leader within the NPHA, and MMS is a Principal Surveillance

Officer with experience in national surveillance and outbreak response.

~~The research team comprised investigators with backgrounds in public health, medicine,~~

~~nursing, medical anthropology, and community health. The principal investigator~~

~~(ENI)~~LN is a ~~physician and public health researcher based in Sierra Leone with extensive~~

~~experience in qualitative health research and implementation science. The co~~doctorate-

~~investigator (LN) is a~~trained social scientist and mental health expert with training in

qualitative methods and prior experience in HIV and infectious disease research in East and West Africa. Research assistants (n=8) were recruited from Sierra Leone; all held at minimum a bachelor’s degree in a health-related field, and four had prior experience in qualitative data collection during the Ebola response. Research assistants received five days of training covering qualitative methods, ethical considerations, sensitive interviewing, emotional support provision, and mpox-specific knowledge. The team included members with prior experience in Ebola response research, providing valuable contextual understanding [~~12~~9,~~13~~10].

We maintained reflexive practices throughout, recognising that researchers’ positions— as healthcare professionals, public health practitioners, and Sierra Leoneans navigating the same outbreak—shaped data collection and interpretation. Weekly team debriefings addressed emerging insights, analytic decisions, and researcher ~~wellbeing~~well-being.

Reflexive memos documented team members’ evolving understandings, emotional responses, and methodological considerations.

**Inclusivity in global research**

Additional information regarding ethical, cultural, and scientific considerations specific

**Formatted:** Not Highlight

to inclusivity in global research is included in the Supporting Information (S7 Appendix).

This study was conceived, approved, implemented, analysed, and interpreted from within

Sierra Leone’s public health system. It was not organised as a short-term external or

visiting research project. The inclusion of LN reflected complementary qualitative and

mental health expertise, while study leadership, institutional authority, field

implementation, and interpretation remained grounded in Sierra Leonean public health

institutions and local research personnel.

**Data analysis**

Analysis followed the Framework Method, enabling systematic cross-case comparison and

transparent matrix-based synthesis [32,33]. Analysis proceeded through six phases:

~~Analysis followed the Framework Method, enabling systematic cross-case comparison and~~

~~transparent matrix-based synthesis [33,34]. Analysis proceeded through six phases: (1)~~

Familiarisation: Analysts read complete transcripts multiple times, noting initial observations; (2) Developing an analytical framework: Initial coding of a subset of transcripts to develop a working codebook informed by the SEM/HSDF theoretical framework and emergent concepts; (3) Indexing: Systematic coding of all transcripts using the agreed codebook; (4) Charting: Summarising coded data into framework matrices organised by theme and participant group; (5) Cross-case analysis: Identifying patterns and variations across participants within and between stakeholder groups; (6) Interpretation: Developing higher-order interpretations connecting findings to broader literature and implications.

Because stigma formed part of the study aims and the HSDF sensitising framework, the

analysis did not treat the mere presence of stigma as wholly inductive. The primary

analytical question was not whether stigma existed, but what forms and mechanisms of

stigma emerged and how these affected individuals, families, communities, healthcare

workers, contact tracing, and system response. We therefore distinguished between

framework-informed domains and data-led extensions of those domains during coding,

charting, and interpretation.

A critical analytical decision concerned how to integrate and differentiate perspectives across the four participant groups. We constructed separate framework matrices for each stakeholder group before conducting cross-group analysis, ensuring that patterns within each group were identified before comparisons were drawn. In our interpretive process, we explicitly distinguished between first-person experiential accounts (primarily from

survivors), professional observational accounts (primarily from healthcare workers and contact tracers), and community normative perspectives (primarily from community members). Where healthcare workers or contact tracers spoke about patient experiences, ~~we treated these as institutionally situated observations rather than direct evidence of lived~~

~~experience, and we note this distinction in our presentation of findings. Convergence~~

~~across groups was interpreted as strengthening credibility, while divergence prompted~~

~~further analytical interrogation of the epistemic basis for differing accounts.~~

we treated these as institutionally situated observations rather than direct evidence of

lived experience, and we note this distinction in our presentation of findings.

Convergence across groups was interpreted as strengthening credibility, while divergence

prompted further analytical interrogation of the epistemic basis for differing accounts.

Data management ~~utilised~~was conducted using NVivo 14 (Lumivero, 2023). Coding was

led by ENI and LN, both of whom had doctoral-level training in qualitative research

methods ~~at doctoral level~~. ENI ~~is based in Sierra Leone and~~ brought ~~contextual~~embedded

knowledge of ~~the~~Sierra Leone’s health system and outbreak response; expertise while LN

brought a comparative perspective from qualitative research experience in East African

settings. A team-based approach to coding was employed, with initial independent coding by both analysts followed by consensus discussions to resolve discrepancies. An audit trail documented all analytic decisions (S4 Appendix). Subgroup analyses examined variations by gender, age group (18–24, 25–34, 35–49, 50+), district, urban/rural residence, education level, and for healthcare workers, professional role and facility type. Given the qualitative nature of the study and modest subgroup sizes, we report observed patterns as exploratory

observations rather than ~~statistically~~generalisable ~~tested~~subgroup differences, and we

exercise caution in interpretation where cell sizes are small. We also attended to how social positions may intersect—for example, how gender and rural residence jointly shaped stigma experiences—rather than treating demographic categories as independent, parallel

variables. Member checking was conducted with 20 participants (5 from each stakeholder group) who reviewed preliminary findings and provided feedback that was incorporated

into the final analysis.

~~Ethical considerations~~

~~Ethical approval was obtained from the Sierra Leone Ethics and Scientific Review Committee (SLESRC-2025-049) on 28~~ ~~February 2025. Given the potentially stigmatising nature of mpox, particular attention was paid to confidentiality protections:~~ ~~participants selected interview locations offering privacy; identifiers were removed during transcription; quotes are presented~~ ~~using pseudonyms and with identifying details altered; and data files are stored on encrypted, password-protected servers~~ ~~accessible only to the research team.~~

~~Emotional distress protocols were established given the sensitive subject matter. Research assistants were trained to~~ ~~recognise distress signals and offer supportive responses. Referral pathways to mental health services were established in~~ ~~each district through partnership with Partners in Health Sierra Leone. Participants were informed they could pause, skip~~ ~~questions, or terminate interviews at any time. Post-interview check-ins were conducted via telephone within one week for~~ ~~survivor participants. Participants received transportation reimbursement (NLE 50, approximately USD 2.50 at June 2025~~ ~~exchange rates) and refreshments but no other compensation to minimise coercion.~~

**Results**

**Participant characteristics**

Table 1 presents participant demographics. Among the 42 mpox survivors, 26 (61.9%)

were male and 16 (38.1%) were female, with a mean age of 28.4 years (range: 18–52).

The majority resided in Western Area Urban (n=25, 59.5%), followed by Western Area

~~Table 1 presents participant demographics. Among the 42 mpox survivors, 26 (61.9%)~~

~~were male and 16 (38.1%) were female, with a mean age of 28.4 years (range: 18–52).~~

~~The majority resided in Western Area Urban (n=25, 59.5%), followed by Western Area~~

Rural (n=9, 21.4%), Bo (n=5, 11.9%), and Kenema (n=3, 7.1%). Time since recovery ranged from 2–14 weeks at interview. Among healthcare workers (n=38), 22 (57.9%) were nurses, 8 (21.1%) were physicians, 5 (13.2%) were laboratory technicians, and 3 (7.9%) were IPC officers. Community members (n=36) were distributed across all four districts with deliberate oversampling from communities with the highest case burdens.

~~Contact tracers (n=24) represented all District Health Management Teams involved in~~

~~mpox response.~~

Contact tracers (n=24) represented all DHMTs involved in mpox response.

**Table 1. Demographic characteristics of study participants.**

| **Characteristic** | **Survivors (n=42)** | **Healthcare Workers~~CWs~~ (n=38)** | **Community ~~(n=36)~~**  **(n=36)** | **Contact Tracers ~~(n=24)~~**  **(n=24)** |
| --- | --- | --- | --- | --- |
| Gender – Male | 26 (61.9%) | 15 (39.5%) | 18 (50.0%) | 16 (66.7%) |
| Gender – Female | 16 (38.1%) | 23 (60.5%) | 18 (50.0%) | 8 (33.3%) |
| Age (mean, range) | 28.4 (18–52) | 36.2 (24–58) | 38.7 (22–65) | 32.1 (23–48) |
| Western Area ~~Urban~~  Urban | 25 (59.5%) | 22 (57.9%) | 14 (38.9%) | 10 (41.7%) |
| Western Area ~~Rura~~l  Rural | 9 (21.4%) | 8 (21.1%) | 10 (27.8%) | 6 (25.0%) |
| Bo District | 5 (11.9%) | 5 (13.2%) | 6 (16.7%) | 4 (16.7%) |
| Kenema District | 3 (7.1%) | 3 (7.9%) | 6 (16.7%) | 4 (16.7%) |

Analysis identified five overarching themes with multiple subthemes, summarised in the thematic framework (S1 Fig), with the full diagram and explanation provided in S1 Appendix. ~~Stigma~~Distinct mechanisms through which stigma manifested appeared across

**Formatted Table**

all five themes. We present each theme with illustrative quotations, indicating participant characteristics using codes (e.g., S-M-28-WAU = survivor, male, age 28, Western Area Urban). Extended quotations supporting each theme and subtheme appear in S6 Appendix.

**~~S1 Fig. Thematic framework showing five overarching themes and their~~**

**~~interconnections.~~** *~~The full framework diagram and explanation are provided in S1~~*

*~~Appendix.~~*

**Theme 1: “The body that betrays”—Illness experiences**

Survivors described illness experiences characterised by physical suffering, diagnostic uncertainty, and lasting bodily changes. This theme encompassed four subthemes: onset and recognition, physical suffering, diagnostic journeys, and altered body experience.

**Onset and recognition**

Initial symptom recognition was frequently delayed or misattributed, particularly in the outbreak’s early phases. Many survivors described initially interpreting symptoms through familiar illness categories—malaria, skin infections, or sexually transmitted infections:

*“I first felt the fever coming and thought, this is malaria again. I took the [antimalarial] tablets and waited. When the bumps started appearing on my skin, I thought maybe it was an allergic reaction. It was only when my friend told me about mpox being in Freetown that I started to worry it could be that.” (S-M-24- WAU)*

**Physical suffering**

Survivors described intense physical suffering that often exceeded their expectations. Pain was a dominant feature, particularly associated with lesions, lymphadenopathy, and systemic symptoms. Survivors frequently reported limited access to effective pain relief (S6 Appendix):

*“The pain was like nothing I have experienced before. Every lesion felt like fire burning on my skin. I could not sleep, I could not eat, and I could not even lie*

*down comfortably. The lymph nodes under my arms were so swollen that I could*

*not lower my arms to my sides. I thought I was going to die.” (S-M-35-WAU)*

**Diagnostic journeys**

Pathways to diagnosis were often circuitous, involving multiple healthcare encounters. Several survivors described initial misdiagnosis:

*“The first clinic I went to, they said it was chickenpox and gave me calamine lotion. After two days, I went back because it was getting worse, and they said*

*maybe it was syphilis and gave me injections. It was only when I went to [government hospital] that they finally said it could be mpox and took the swab.” (S-M-29-WAU)*

**Altered body experience**

Beyond acute illness, survivors described lasting changes in their relationship with their bodies, including scarring and impacts on sexuality and intimacy:

*“Since I recovered, I have not been able to be intimate with my husband. Every time I think about it, I remember the sores in that area, and I feel afraid and*

*~~ashamed. He is patient with me but I can see he is frustrated. I do not know if~~*

*~~this feeling will ever go away.” (S-F-34-WAU)~~*

*ashamed. He is patient with me, but I can see he is frustrated. I do not know if this*

*feeling will ever go away.” (S-F-34-WAU)*

**Theme 2: “Marked and marginalised”—Multi-layered stigma**

~~Stigma~~Distinct mechanisms through which stigma manifested emerged across all

participant groups and study sites. ~~It~~Stigma operated at multiple levels and through

~~various mechanisms. Four~~four interconnected ~~subthemes were identified~~forms: enacted

stigma and discrimination, perceived stigma and anticipated rejection, internalised stigma and self-blame, and associative stigma (see S6 Appendix).

**Enacted stigma and discrimination**

Survivors reported direct experiences of discrimination from multiple sources, including family rejection, community avoidance, and healthcare discrimination:

*“When my family found out I had mpox, my own brother told me I could not stay in the house anymore. He said I would infect his children. I had to go and stay with a friend who was the only one willing to take me in.” (S-M-26-WAR)*

**Perceived stigma and anticipated rejection**

Perceived stigma shaped behaviour, particularly around disclosure:

*“I told no one except my mother and one close friend. Not even my employer—I said I had malaria and needed rest. I was so afraid that if people knew, I would lose everything.” (S-F-27-BO)*

**Internalised stigma and self-blame**

Many survivors described accepting negative social messages, leading to shame and self- blame, particularly when illness was attributed to sexual behaviour:

*“I kept asking myself, what did I do to deserve this? I felt so dirty, so ashamed. I thought maybe God was punishing me for something I had done. I could not even look at myself in the mirror.” (S-M-25-WAU)*

**Associative stigma**

Stigma extended to families, caregivers, and those associated with outbreak response. Both survivors and healthcare workers described this pattern. Healthcare workers reported being avoided by neighbours and community members because of their roles:

*“My neighbours know I work at the mpox treatment centre. Some of them have started avoiding me. One woman actually told me she does not want me coming near her children because I might bring the disease to the community. It is hurtful because I am trying to help people.” (HCW-F-34-WAU-Nurse)*

**Theme 3: “Seeking care in silence”—Healthcare-seeking trajectories**

Multiple factors influenced healthcare-seeking behaviours, including stigma, knowledge, economic constraints, and health system accessibility. Three subthemes characterised these trajectories: delays and barriers, alternative care pathways, and facilitators of timely care (S6 Appendix).

**Delays and barriers**

Fear of stigma was the most frequently cited reason for delayed care-seeking:

*“I waited because I was afraid of what would happen if people found out. I thought maybe if I just stayed home, it would go away on its own, and no one*

*would ever need to know.” (S-M-22-WAU)*

Care-seeking delays varied across social position and geography, but these observations

were exploratory rather than comparative. Urban participants more often described

concealment strategies shaped by fear of disclosure at work or within dense social

networks. Rural participants more often described transport costs, distance to facilities,

and limited diagnostic access. Participants in formal employment described fear of

workplace disclosure and job loss, while those in informal work emphasised income loss

from missing daily labour. Because several subgroups were small, particularly outside

Western Area, these patterns are presented narratively rather than as subgroup

comparisons.

~~Patterns in care-seeking delays varied across participant subgroups. Table 2 summarises the barriers most frequently~~ ~~described by each group. We note that subgroup sizes are modest, and these comparisons illustrate qualitative variation~~ ~~rather than generalisable differences.~~

**~~Table 2. Observed patterns in healthcare-seeking delays by subgroup characteristics.~~**

**~~Subgroup~~**

**~~Key barriers described by participants~~**

~~Urban residents~~

~~Fear of disclosure, work concerns, elaborate concealment strategies~~ ~~Rural residents~~

~~Distance to facilities, transport costs, limited diagnostic capacity~~ ~~Higher education~~

~~Disclosure concerns, workplace stigma, awareness of long-term implications~~ ~~Lower education~~

~~Limited symptom knowledge, financial constraints, reliance on familiar illness categories~~ ~~Formal employment~~

~~Fear of job loss, disclosure to employer, navigating sick leave~~ ~~Informal employmen~~t

~~Income loss from missed work, no sick leave provisions, competing livelihood priorities~~

**Facilitators of timely care**

Survivors who sought care promptly described factors that facilitated their decision,

including awareness of mpox symptoms from public health messaging, social support, and prior positive healthcare experiences:

*“I had seen the messages on the radio about mpox and what signs to look for. When I started having those exact symptoms, I knew I should go get tested right away.” (S-M-33-WAU)*

**Theme 4: “Working at the edge”—Healthcare worker experiences**

Healthcare workers described experiences of responding to the outbreak while navigating resource constraints, occupational risks, emotional burdens, and community pressures. ~~Four subthemes captured these experiences (S6 Appendix).~~

Four subthemes captured these experiences (S6 Appendix).

**Clinical challenges and moral distress**

Healthcare workers described confronting clinical situations for which they felt inadequately prepared:

*“There were times when we had more patients than beds. We had to make difficult decisions about who could be admitted and who had to go home. Some patients we sent home were very sick, but we had no space for them. I would lie awake at night wondering if I had made the right choice.” (HCW-M-38-WAU- Physician)*

**Emotional toll and compound trauma**

The emotional burden was substantial. Many healthcare workers who had served during previous epidemics described the mpox response reactivating earlier distress:

*“When mpox started spreading, it brought back all those memories from Ebola. The fear, the death, watching colleagues get sick. I thought I had processed all of ~~that but it came flooding back. Some nights I could not sleep.” (HCW-F-45-WAU-~~*

*~~Nurse)~~*

*that but it came flooding back. Some nights I could not sleep.” (HCW-F-45-WAU-*

*Nurse)*

**Theme 5: “Building the plane while flying”—Health system response**

The final theme addresses the health system response, drawing primarily on accounts

from	healthcare	~~worker~~workers	and	contact	~~tracer	accounts~~tracers.

~~Subthemes~~Participants ~~included~~described a response that was adaptive but strained, with

frontline teams often adjusting referral pathways, communication practices, and

community engagement strategies as the outbreak evolved. Four subthemes characterised

this domain: adaptive capacity and ~~innovation~~improvisation, coordination challenges,

vaccination experiences, and contact tracing barriers~~, and vaccination experiences~~ (S6

Appendix).

Adaptive capacity and improvisation

Frontline teams described adjusting their work in real time as case numbers increased and

formal systems struggled to keep pace.

*“There was no perfect system at the beginning. Every day the situation changed,*

*so we also changed how we worked. Sometimes the official message came late, and*

*we used phone calls, WhatsApp groups, and people we trusted in the community to*

*move faster. It was not ideal, but if we waited for everything to be arranged, the*

*cases would move ahead of us.” (CT-M-32-WAU)*

Beyond communication improvisation, frontline adaptation extended to treatment

capacity management. Healthcare workers described facilities that reached capacity

rapidly during the outbreak’s acceleration phase, requiring informal arrangements for

patient transfer between centres, rotation of staff, and triage of cases for home-based

isolation when admission was not possible. Referral pathways were negotiated in real

time rather than through formal protocols, and transport availability—for both patients

and specimens—was a recurrent constraint that affected the speed of both clinical

response and surveillance (S6 Appendix).

*Coordination challenges*

*Participants described delays and fragmentation in information flow across*

*laboratory, surveillance, treatment, and field teams.*

*“Sometimes the laboratory result, the case list, and the field team were not moving*

*at the same speed. A patient could be confirmed, but by the time the information*

*reached us, the family had already moved or the contacts were no longer easy to*

*find. The problem was not that people refused to work. The problem was that the*

*information was coming in pieces.” (CT-F-29-WAR)*

Transport constraints compounded information delays. Specimen movement between

collection points and laboratories depended on vehicles not always prioritised for

surveillance functions. Contact tracers in rural and peri-urban areas described situations

in which the window for identifying and isolating contacts had passed by the time

laboratory confirmation reached field teams. These gaps were understood by participants

as systemic failures of coordination rather than individual lapses, but they accumulated

into measurable delays in the response chain (S6 Appendix).

*Vaccination experiences*

*Vaccination was described as protective, but also socially sensitive in communities*

*where mpox was associated with shame, exposure, or sexual transmission.*

*“People wanted protection, but they were also afraid of what others would think if*

*they joined the vaccination line. Some said, ‘If they see me there, they will say I*

*have been exposed.’ So the vaccine itself became connected to the same shame as*

*the disease. We had to explain that vaccination was protection, not a confession.”*

*(HCW-F-36-WAU-Nurse)*

Vaccine prioritisation and eligibility also created operational and social tension. Initial

supplies were limited, and allocation decisions—across healthcare workers, close

contacts, and community members—were experienced as inconsistent. Healthcare

workers at peripheral facilities described receiving later access to vaccination than those

at referral centres, which they perceived as increasing their occupational exposure during

the outbreak’s early phase. Community members described uncertainty about eligibility

criteria: some understood vaccination as reserved for confirmed contacts, while others

were unsure whether seeking vaccination required disclosure of potential exposure. These

ambiguities, compounded by the social stigma attached to vaccination in some

communities, limited uptake among those who might have benefited most (S6 Appendix).

**Contact tracing barriers**

**Formatted:** Not Highlight

*Contact tracing was particularly difficult when naming contacts could expose*

*sexual relationships, household conflict, workplace absence, or socially*

*disapproved behaviour.*

~~Contact tracing was described as particularly challenging, with stigma undermining cooperation:~~

*“Many patients are reluctant to name their contacts, especially if the contact was through sexual activity. They are afraid of what will happen to those people, or they do not want to reveal their private behaviour.” (CT-M-34-WAU)*

**Formatted:** Not Highlight

Beyond sexual exposure, contact tracers described how household tensions, employment

concerns, and community visibility created additional barriers. Participants in informal

employment feared that being named as a contact would render their illness visible to

employers or community leaders, threatening their livelihoods. In some cases, contact

tracers described extending timelines informally to allow participants to disclose to

family members before notification proceeded, balancing surveillance objectives against

participant safety and consent (S6 Appendix).

**Subgroup variations**

*~~Table~~Across ~~3~~the ~~summarises~~findings, ~~variations~~subgroup ~~observed~~variation*

*~~across~~appeared ~~subgroups~~suggestive but not definitive. ~~Gender differences were~~*

*~~notable: female~~Female survivors more frequently described internalised stigma*

*~~connected~~linked to perceived sexual transmission ~~implications~~and intimate*

*relationships, while male survivors more often ~~described~~emphasised economic*

*impacts ~~from~~and work disruption. Younger adults described peer-level stigma,*

*social media disclosure fears, and economic vulnerability. Older participants more*

*often described family-based discrimination and concerns compounded by pre-*

*existing health conditions. Rural participants generally described longer care-*

*seeking pathways, whereas urban participants described more elaborate*

*concealment strategies. These ~~gendered~~observations ~~patterns~~are ~~were~~exploratory*

*and should not ~~uniform—younger~~be ~~women~~interpreted ~~in~~as ~~urban~~generalisable*

*~~areas~~subgroup ~~also described economic concerns similar to those raised by male~~*

*~~participants~~differences.*

~~Rural participants generally described longer pathways to care, though this varied with proximity to treatment centres and~~ ~~prior health system engagement. Urban participants described more elaborate concealment strategies. We present these~~ ~~observations as descriptive patterns from a modest sample rather than definitive subgroup differences.~~

**~~Table 3. Summary of observed subgroup variations in key findings.~~**

**~~Subgroup~~**

**~~Key observations~~**

~~Female survivors~~

~~More frequently described internalised stigma; concerns about perceived sexual transmission implications~~ ~~Male survivors~~

~~Greater emphasis on economic impacts and work disruption~~ ~~Age 18–24~~

~~More peer-level stigma described; social media disclosure fears; economic vulnerability among those in informal work~~ ~~Age 50+~~

~~More family-based discrimination described; intergenerational tensions; compounded by pre-existing health concerns~~ ~~Rural residence~~

~~Longer care-seeking pathways described; limited facility access; varied with proximity to treatment centres~~ ~~Urban residence~~

~~More elaborate concealment strategies described; workplace concerns~~

**Discussion**

This study provides an in-depth qualitative exploration of mpox outbreak experiences in Sierra Leone, revealing ~~the~~ physical suffering, multi-layered stigma, healthcare-seeking

challenges, healthcare worker distress, and health system response dynamics

~~characterising~~during the clade IIb epidemic. While certain ~~themes—such~~themes,

~~as~~including disease-related stigma, healthcare-seeking delays, and healthcare worker

moral ~~distress—resonate~~distress, resonate with prior research on infectious disease

outbreaks, our findings contribute ~~novel~~empirical and conceptual insights ~~in several~~

~~respects~~ that extend beyond contextual confirmation of known patterns [28,29~~,30~~].

Three contributions merit particular emphasis. First, this study provides what is, to our knowledge, the first qualitative account of mpox illness experiences from an African setting during the current PHEIC, offering empirical grounding for understanding how mpox is experienced in a context characterised by post-conflict health system fragility, sequential epidemic exposure, and cultural frameworks distinct from those documented in high-income country studies of the 2022 outbreak. Second, our multi-perspective design reveals how stigma operates not merely as a barrier at discrete points in the care pathway but as a ~~pervasive~~recursive structural ~~force~~process that simultaneously shapes illness

experience, healthcare-seeking, professional practice, contact tracing, and system-level

~~response—a cross-cutting dynamic that existing frameworks may inadequately~~

~~capture~~response. Third, the phenomenon of compound trauma among healthcare

~~workers—where~~workers, where mpox response reactivated unresolved distress from

Ebola and COVID-19 ~~experiences—represents~~experiences, represents a distinctive

finding with implications for workforce sustainability in settings with histories of sequential health emergencies.

**Illness experiences and disrupted embodiment**

Survivors’ narratives of physical suffering align with emerging clinical literature documenting severe pain as a hallmark of mpox, particularly when genital lesions are present [7,~~38~~37]. Our qualitative findings add experiential depth to these clinical

observations, revealing how inadequate pain management compounded suffering and eroded trust in healthcare. The phenomenon of “disrupted embodiment”—fundamentally altered relationships with one’s own body—has been documented in other illness contexts but has not previously been explored in relation to mpox [~~39~~38]. Visible scarring and

impacts on sexuality represent lasting consequences extending well beyond acute illness resolution. It should be noted, however, that our cross-sectional design captures experiences at a single point in recovery; longitudinal research would be needed to understand how embodied experiences evolve over time and whether the disruptions described here are enduring or gradually resolve [~~19~~18].

**Stigma mechanisms and impacts**

Our findings extend the ~~Health Stigma and Discrimination Framework~~HSDF to the mpox

context, documenting how stigma operates through multiple mechanisms—enacted, perceived, internalised, and associative—at multiple levels from individual to institutional [~~16~~15]. The visible nature of mpox lesions, coupled with perceived associations with

sexual transmission, creates a particularly stigmatising configuration [~~17~~16,~~40~~39].

Comparisons to HIV disease were explicit in participant accounts, echoing patterns

documented in the 2022 global outbreak [18,19,~~20,23~~22].

Critically, our findings illustrate how stigma undermined outbreak control in this setting. Fear of stigma delayed care-seeking, enabling ongoing transmission. Anticipated discrimination reduced disclosure to contacts, hampering contact tracing. Internalised stigma affected mental health and recovery. These pathways indicate that stigma warrants attention not only as a social harm but as a public health concern integral to outbreak response effectiveness [~~13~~12,~~23~~22,~~41~~40].

**Theoretical implications**

Our findings have implications for the theoretical frameworks that guided the analysis. The

~~Social~~study ~~Ecologica~~ldid ~~Model~~not seek to establish whether stigma existed in the outbreak;

rather, it examined the forms, mechanisms, pathways, and effects through which stigma shaped

lived experience and response practice. This distinction matters theoretically because the empirical

contribution lies in the movement of stigma across levels, not in its presence as a theme. The SEM

provided a useful heuristic for organising multi-level findings, but the empirical data suggest that ~~the~~ relationships between levels are more dynamic and recursive than the model’s nested structure

implies. Stigma~~, in particular,~~ did not operate as a discrete factor at any single level. ~~but~~It functioned

as a cross-cutting structural force that simultaneously shaped individual cognition, interpersonal relationships, community norms, healthcare encounters, surveillance practices, and institutional

~~practices~~response. This finding suggests that the SEM’s conventional representation of levels as

relatively independent layers may need adaptation for outbreak contexts, where a single social

~~process—stigma—permeates~~process can permeate and ~~connects~~connect all levels simultaneously.

Similarly, the ~~Health Stigma and Discrimination Framework~~HSDF proved valuable for

categorising stigma mechanisms, but our findings suggest areas for extension. The framework’s typology of enacted, perceived, internalised, and associative stigma captured important dimensions~~,~~ ~~yet~~of participants’ accounts, ~~revealed that~~yet these mechanisms

operated in reinforcing cycles rather than as parallel categories. Enacted stigma fuelled

perceived stigma, ~~which~~perceived ~~in~~stigma ~~turn~~encouraged ~~drove~~concealment,

concealment ~~behaviours~~delayed ~~that~~care-seeking ~~paradoxically~~and contact disclosure, and

delayed disclosure intensified community suspicion and further enacted stigma. This

cyclical dynamic, while implied by the framework, deserves more explicit theorisation, particularly in outbreak contexts where the temporal compression of an epidemic may accelerate stigma cycles in ways not fully captured by frameworks developed for chronic conditions such as HIV. Future research might explore how the HSDF could be adapted to account for the rapid onset, acute visibility, sexual and moral associations, and epidemic

tempo of mpox-related stigma compared ~~to~~with the more gradual stigma trajectories

characteristic of chronic infectious diseases [~~41~~40].

Beyond the cyclical dynamic, several inductive extensions of the HSDF emerged from the

data that were not anticipated by the framework. Associative stigma, conventionally

documented toward family members and caregivers, extended in this study to contact

tracers and outbreak response personnel who were stigmatised by association with the

response itself rather than with illness. Institutional settings associated with mpox care—

treatment centres and vaccination sites—also carried stigmatising meaning: participants

avoided vaccination queues and treatment facilities to prevent public identification with

the disease, a form of institutionally located stigma not captured by the framework’s

original typology. Religious and moral idioms shaped internalised stigma in ways that

extended beyond the framework’s conception of self-stigma; survivors drew on spiritual

frameworks of punishment and moral failing to account for their illness, reflecting

culturally specific self-attribution processes absent from frameworks developed primarily

in secular, high-income contexts. Finally, informal stigma-mitigation practices emerged

spontaneously among frontline response teams: contact tracers and healthcare workers

developed strategies to reduce the visibility of response activities, adopt neutral language

in community settings, and avoid publicly identifying individuals through observable

behaviour. These adaptations represent a form of stigma-responsive practice grounded in

lived outbreak experience and warrant recognition in response training and preparedness

guidance.

**Healthcare ~~worker~~workers’ experiences**

Healthcare workers’ narratives of moral distress, occupational risk anxiety, and emotional burden align with documented experiences from other outbreaks while revealing mpox-specific dimensions [22,23,24,25,26~~,27~~]. The compound effect of sequential outbreak ~~response—~~

**Formatted:** Not Highlight

~~many~~response was especially salient. Many participants had served during both Ebola and COVID-

~~19—suggests~~19 ~~cumulative~~before ~~workforce~~being ~~impacts~~asked ~~warranting~~to ~~attention~~respond

~~[9,28]~~.to ~~Moral distress arising from resource constraints represents a systemic issue requiring~~

~~health system strengthening~~mpox, ~~not~~and ~~merely~~they ~~individual-level~~described ~~support~~earlier

~~[24]~~.memories, ~~We~~fears, ~~note~~and ~~that~~losses ~~healthcare worker accounts in this study primarily~~

~~reflect the perspectives of those who remained in their posts~~returning during the ~~outbreak;~~new

response. This pattern is not fully captured by burnout alone. Burnout offers an important

comparator because it describes emotional exhaustion, depersonalisation, and reduced personal

accomplishment arising through chronic occupational strain [27,41]. In this study, however,

healthcare workers described the ~~experiences~~reactivation of ~~those~~prior ~~who~~outbreak-related

~~left~~fear, ~~clinical~~grief, ~~practice~~and moral conflict across sequential public health emergencies. We

therefore use compound trauma descriptively to refer to the cumulative emotional and professional

burden produced when successive epidemic responses reactivate prior exposure before adequate

recovery or ~~were~~institutional ~~unable~~repair ~~to continue responding remain undocumented and may~~

~~differ substantially~~occurs.

This descriptive interpretation is anchored in, but does not attempt to develop, the wider literature

on moral distress, moral injury, and cumulative disaster exposure. Moral distress describes knowing

the ethically appropriate action while being constrained from acting [42], and the crescendo effect

explains how moral residue can accumulate across repeated incidents [43]. Moral injury captures

longer-term psychological, social, moral, and identity-related effects of witnessing, failing to

prevent, or participating in events that transgress deeply held moral beliefs [44,45]. Disaster

literature similarly shows that multiple or successive exposures can deepen mental health effects

and delay recovery, particularly when events occur in close temporal proximity [46,47,48]. A living

systematic review on health and care workers during COVID-19 and other public health

emergencies of international concern likewise highlights mental health, well-being, and quality-of-

life impacts as core workforce consequences of emergencies [49]. In Sierra Leone, the relevance

of these concepts is heightened by the memory of Ebola, the COVID-19 response, recurrent

resource scarcity, and repeated expectations that frontline staff absorb risk on behalf of the public

[9,22]. We therefore present compound trauma as an empirical pattern requiring further dedicated

conceptual and longitudinal study, not as a fully settled diagnostic or theoretical construct.

The implication is that psychosocial support for healthcare workers cannot be treated as an episodic

add-on during each outbreak. Settings with recurrent epidemic exposure require continuity of

workforce well-being programmes, access to confidential mental health support, peer support

systems, supportive supervision, and institutional recognition of moral distress. We note, however,

that healthcare worker accounts in this study primarily reflect the perspectives of those who

remained in their posts during the outbreak; the experiences of workers who left clinical practice

or were unable to continue responding remain undocumented and may differ substantially.

**Implications for practice and policy**

Our findings suggest several implications for mpox response in Sierra Leone and potentially in similar settings, though we note that these are indicative rather than ~~prescriptive, and their applicability will depend on local context and implementation~~

~~considerations.~~

prescriptive, and their applicability will depend on local context and implementation

considerations.

First, the pervasive role of stigma across all domains of the outbreak experience suggests that stigma-informed strategies merit integration into outbreak management frameworks from the outset, rather than being treated as secondary concerns [~~23~~22,~~41~~40]. This could

include attention to language used in public communications, protection of patient confidentiality, community engagement to address misinformation and fear, and support for affected individuals facing discrimination [~~42~~50]. The specific forms that such

strategies might take require further development and evaluation.

Second,	healthcare worker accounts suggest that support	~~mechanisms—~~

~~including~~mechanisms, including adequate PPE, ~~and~~access to vaccination ~~access~~,

psychosocial support, strategies to address associative stigma, and career protections for outbreak ~~responders—may~~responders, ~~warrant~~should ~~establishment~~be established as

integral components of the response ~~components~~architecture rather than as afterthoughts

[25,26~~,27~~]. The compound trauma observed among workers with prior epidemic

experience further suggests the potential value of continuity in workforce wellbeing programmes across successive outbreaks.

Third, the care-seeking patterns observed in this study suggest that community-centred communication approaches—emphasising symptom recognition and care availability, delivered through trusted community channels—may facilitate timely care-seeking [~~41~~40,~~42~~50]. However, our data capture what participants reported about their care-

seeking experiences and decisions, and social desirability considerations may have shaped these accounts. Intervention research would be needed to evaluate the effectiveness of specific communication strategies.

**Limitations**

Several limitations should be acknowledged~~, some of which have already been noted in~~

~~the discussion of specific findings~~. First, ~~our~~although the sample~~,~~ ~~while~~was purposively

diverse across participant groups and districts, it may not represent all ~~experiences—~~

~~particularly~~experiences ~~those~~of the outbreak. In particular, people who never accessed

healthcare, ~~or~~those who declined participation because of stigma, and those who

experienced severe outcomes~~. Survivor participants, by definition, had recovered~~

~~sufficiently to participate, potentially excluding the most severe cases. Second, recall bias~~

may ~~affect~~be ~~accounts of illness trajectories, particularly given the emotional intensity of~~

~~the experiences described~~underrepresented. ~~Third, social desirability bias may have~~

~~influenced responses across all participant groups: survivors may have understated risky~~

~~behaviours, community members may have moderated stigmatising attitudes, and~~

~~healthcare workers may have emphasised challenges to advocate for resources. Fourth,~~The

geographic concentration of cases in Western Area may also limit transferability ~~of~~

~~findings~~ to districts with different epidemiological and social profiles.

Second, the study relied on retrospective self-reported accounts collected during a highly

sensitive outbreak. Recall bias may have affected illness narratives, and social desirability

bias may have shaped responses across participant groups. Survivors may have understated

socially sensitive exposures or behaviours, community members may have moderated

stigmatising attitudes, and healthcare workers may have emphasised operational

constraints. Sexual exposures and partner-related experiences were also likely

underreported because of stigma, fear of disclosure, and the social risks attached to

discussing sexual behaviour in this setting. We therefore make no claims about individual

transmission routes.

Third, the study aims and HSDF-informed sensitising framework directed analytical

attention toward stigma-related experience. This may have increased attention to stigma

compared with other possible dimensions of the outbreak experience. To reduce this risk,

we treated the framework as a sensitising tool rather than a rigid coding template, and the

specific forms, mechanisms, pathways, and effects of stigma reported here were developed

from participant accounts rather than imposed in advance.

Fourth, although individual interviews were used for sensitive survivor accounts and for

healthcare workers’ more personal experiences, some healthcare worker FGDs may have

been affected by hierarchy and workplace dynamics. Facilitators attempted to mitigate this

by monitoring group interaction, inviting dissenting views, and offering follow-up SSIs

where needed, but some participants may still have moderated criticism of colleagues,

supervisors, or institutional practices.

Fifth, the cross-sectional nature of data collection means that ~~our~~the ~~findings~~study

~~capture~~captures experiences at particular points in the outbreak trajectory and ~~in individual~~

recovery ~~processes;~~process. ~~how these experiences evolve over time—particularly~~

~~regarding~~How stigma, embodiment, care-seeking, and healthcare worker ~~wellbeing—~~

~~remains~~distress evolve over time remains an important question for longitudinal research.

~~Sixth, the~~The absence of male partner perspectives, additional family members ~~beyond the~~

~~survivors themselves~~, and individuals who declined healthcare also limits the

~~comprehensiveness~~completeness of our account of the relational dimensions of mpox

experience. ~~Despite these limitations, our multi-perspective approach, diverse sample, and~~

~~rigorous analytic procedures support the credibility of findings within the scope of what~~

~~the study was designed to address.~~

Despite these limitations, the multi-perspective design, diverse sample, member checking,

framework-based analysis, and triangulation across survivor, healthcare worker,

community, and contact tracer accounts support the credibility of the findings within the

scope of the study.

**Conclusions**

Stigma was the dominant social force shaping the mpox outbreak experience in Sierra Leone. It delayed care-seeking, undermined contact tracing, eroded trust in health services, and compounded the emotional burden on healthcare ~~workers—all~~workers, all

of which directly compromised outbreak control. ~~These~~Because ~~findings~~stigma

~~indicate~~was anticipated in the analytical framework, the key finding is not simply that

stigma ~~is not a peripheral social concern~~existed, but athat ~~central~~it ~~obstacle~~operated

~~to~~recursively ~~effective~~across ~~epidemic~~individual, ~~response~~interpersonal, community,

institutional, and system levels.

Healthcare workers with prior Ebola and COVID-19 experience ~~carried~~described a

compound trauma ~~burden~~pattern that current support systems did not fully address.

~~Their~~This ~~accounts~~should ~~point~~not be read as a settled diagnostic construct; rather, it

signals that prior outbreak exposure, unresolved moral distress, and recurrent

expectations of risk need to abe ~~cumulative~~considered in workforce ~~crisis~~support

~~that~~planning. ~~risks~~Health ~~deepening~~worker ~~with~~support systems should therefore extend

across successive outbreaks rather than being rebuilt for each ~~successive epidemic unless~~

~~sustained psychosocial support and career protections are built into response~~

~~architectures~~emergency.

Three priorities emerge for outbreak preparedness in similar post-conflict, resource- constrained settings: (1) stigma-informed strategies integrated from the outset of outbreak response, not appended later; (2) healthcare worker support systems that extend across successive outbreaks rather than being rebuilt each time; and (3) community-centred communication, vaccination, and contact tracing approaches that ~~reaches~~protect

confidentiality and reach people before stigma drives them into silence. These priorities

require further evaluation, but the experiences documented ~~here—from~~here ~~those who~~

~~lived through the outbreak, treated its patients, and traced its spread—make clear~~show that

technical response alone is insufficient without attention to the social and workforce

dimensions of epidemic disease.

**Acknowledgments**

We extend our profound gratitude to all study participants who shared their experiences during an extraordinarily difficult time. We thank the National Public Health Agency leadership for institutional support; the District Health Management Teams of Western Area Urban, Western Area Rural, Bo, and Kenema for facilitating fieldwork; the research assistants who conducted data collection with sensitivity and skill; and the transcriptionists and translators who enabled accurate representation of participants’ words. We acknowledge technical input from Partners in Health Sierra Leone for mental health referral support.

**Author contributions**

**Conceptualization:** Eric Nzirakaindi Ikoona.

**Data curation:** Eric Nzirakaindi Ikoona, Lucy Namulemo.

**Formal analysis:** Eric Nzirakaindi Ikoona, Lucy Namulemo.

**Investigation:** Eric Nzirakaindi Ikoona, Lucy Namulemo, Mary Magdalene Sinnah. Methodology: Eric Nzirakaindi Ikoona, Mohamed Alex Vandi.

**Project administration:** Eric Nzirakaindi Ikoona.

**Supervision:** Foday Sahr.

**Writing – original draft:** Eric Nzirakaindi Ikoona.

**Writing – review & editing:** Eric Nzirakaindi Ikoona, Lucy Namulemo, Mary Magdalene Sinnah, Mohamed Alex Vandi, Foday Sahr.

**Funding**

The authors received no specific funding for this work.

**Competing interests**

The authors have declared that no competing interests exist.

**Data availability statement**

De-identified qualitative data (full transcripts) cannot be shared publicly because they

contain sensitive information and carry a risk of participant re-identification given the

small number of mpox cases in specific geographic areas during the study period. Study

materials supporting transparency and reproducibility are available in the Supporting

Information files: interview and focus group discussion guides (S2–S3 Appendices), the

full coding framework (S4 Appendix), the completed COREQ checklist (S5 Appendix),

and extended de-identified quotations mapped to themes (S6 Appendix). Researchers may

request access to further de-identified data excerpts under a data-sharing agreement.

Requests should be directed—with a brief proposal describing the intended use, data

protection safeguards, and an ethics-compliant data management plan—to the institutional

data steward: Dr. Mohamed Boie Jalloh, Director of Planning, Policy and Research,

National Public Health Agency (email: info@npha.gov.sl; tel. +23277121212). Access is

subject to approval by the Sierra Leone Ethics and Scientific Review Committee,

consistent with the ethics clearance for this study (SLESRC-2025-049). Data will be

retained by the National Public Health Agency for a minimum of five years following

study completion.~~De-identified qualitative data (full transcripts) cannot be shared publicly~~

~~because they contain sensitive information and carry a risk of participant re-identification.~~

~~Researchers can request access by contacting the corresponding author~~

~~(ikoonae@yahoo.com) with a brief proposal describing the intended use, data protection~~

~~safeguards, and an ethics- compliant data management plan. Access is subject to approval~~

~~conditions consistent with the ethics clearance for this study and may require a data sharing~~

~~agreement.~~

**References**

1. World Health Organization. 2024. WHO Director-General declares mpox outbreak a

public health emergency of international concern. Aug 14 [cited 2026 Jan 26]. Available

from: https://[www.who.int/news/item/14-08-2024-who-director-general-declares-mpox-](http://www.who.int/news/item/14-08-2024-who-director-general-declares-mpox-)

outbreak-a-public-health-emergency-of-international-concern

~~1~~2. Ministry of Health (Sierra Leone). 2025. Mpox declared a public health emergency in

Sierra Leone. ~~2025~~ Jan 16 [cited 2026 Jan 26]. Available from: https://mohs.gov.sl/mpox-

declared-a-public-health-emergency-in-sierra-leone/

~~2~~3. UNICEF Sierra Leone. 2025. Fighting Mpox: UNICEF scales up critical support in

Sierra	Leone. ~~2025~~	Jun	18	[cited	2026	Jan	26].	Available	from:

https://[www.unicef.org/sierraleone/press-releases/fighting-mpox-unicef-scales-critical-](http://www.unicef.org/sierraleone/press-releases/fighting-mpox-unicef-scales-critical-) support-sierra-leone

~~3~~4. World Health Organization. 2025. Multi-country outbreak of mpox, external situation

report #54 ~~–~~- 27 June 2025. ~~2025~~ Jun 27 [cited 2026 Jan 26]. Available from:

https://[www.who.int/publications/m/item/multi-country-outbreak-of-mpox--external-](http://www.who.int/publications/m/item/multi-country-outbreak-of-mpox--external-) situation-report--54	27-june-2025

4. ~~World Health Organization. WHO Director-General declares mpox outbreak a public health emergency of internationa~~l ~~concern. 2024 Aug 14 [cited 2026 Jan 26]. Available from: https://~~[~~www.who.int/news/item/14-08-2024-who-director-general-~~](http://www.who.int/news/item/14-08-2024-who-director-general-) ~~declares-mpox-outbreak-a-public-health-emergency-of-international-concern~~
5. Africa Centres for Disease Control and Prevention. 2025. Sierra Leone fights mpox.

~~2025~~ May 18 [cited 2026 Jan 26]. Available from: https://africacdc.org/news-item/sierra-

leone-fights-mpox/

6. Sandi JD, Grant DS, Sahr F, Harding A. 2025. Genomic epidemiology of Mpox virus

in Sierra Leone. Virological [Internet]. ~~2025~~ May 28 [cited 2026 Jan 26]. Available from:

https://virological.org/t/genomic-epidemiology-of-mpox-virus-in-sierra-leone/995

7. Kangbai JB, Saidu E, Foday IK, et al. 2025. Clinical and epidemiological characteristics

among probable and confirmed patients with mpox in Sierra Leone reported from January to	May	2025.	medRxiv.	~~2025~~[Preprint]	[cited	2025	Aug	30].

doi:10.1101/2025.05.30.25328691

8. Mathiang JR, Sahr F, Kamara IF, Ansumana R, Rogers MK, Lee SS, et al. 2025. Rapid

surge of mpox in Sierra Leone: ~~Public~~public health challenges. Int J Infect Dis.

~~2025;~~159:107987. doi:10.1016/j.ijid.2025.107987

9. Raven J, Wurie H, Witter S. 2018. Health workers’ experiences of coping with the Ebola

epidemic in Sierra Leone’s health system: a qualitative study. BMC Health Serv Res. ~~2018;~~18:251. doi:10.1186/s12913-018-3072-3

10. Bah AJ, James PB, Bah N, et al. 2020. Prevalence of anxiety, depression, and post-

traumatic stress disorder among Ebola survivors in northern Sierra Leone: a cross- sectional study. BMC Public Health. ~~2020;~~20:1391. doi:10.1186/s12889-020-09507-6

11. Médecins Sans Frontières. 2025. Responding to an mpox outbreak in the heart of Sierra

Leone. ~~MSF;	2025~~	Jul	8	[cited	2025	Aug	30].	Available	from:

https://[www.msf.org/responding-mpox-outbreak-heart-sierra-leone](http://www.msf.org/responding-mpox-outbreak-heart-sierra-leone)

12. ~~Kupferschmidt K. Explosive mpox outbreak in Sierra Leone overwhelms health systems. Science. 2025 Jun 2 [cited 2025~~ ~~Aug 30]. Available from: https://~~[~~www.science.org/content/article/explosive-mpox-outbreak-sierra-leone-overwhelms-health-~~](http://www.science.org/content/article/explosive-mpox-outbreak-sierra-leone-overwhelms-health-) ~~systems~~

~~13~~12. Fischer LS, Mansergh G, Lynch J, Santibanez S. 2019. Addressing disease-related

stigma during infectious disease outbreaks. Disaster Med Public Health Prep. ~~2019;~~13:989-994. doi:10.1017/dmp.2018.157

~~14~~13. Paterson A, Olliaro P, Rojek A. 2023. Addressing stigma in infectious disease

outbreaks: a crucial step in pandemic preparedness. Front Public Health. ~~2023;~~11:1303679. doi:10.3389/fpubh.2023.1303679

~~15~~14. Link BG, Phelan JC. 2001. Conceptualizing stigma. Annu Rev Sociol. ~~2001;~~27:363-

**Formatted:** English (United States)

385. doi:10.1146/annurev.soc.27.1.363

~~16~~15. Stangl AL, Earnshaw VA, Logie CH, et al. 2019. The health stigma and

**Formatted:** French (France)

discrimination framework: a global, crosscutting framework to inform research, intervention development, and policy on health-related stigmas. BMC Med. ~~2019;~~17:31.

doi:10.1186/s12916-019-1271-3

~~17~~16. Sah R, Mohanty A, Reda A, Padhi BK, Rodriguez-Morales AJ. 2022. Stigma during

monkeypox	outbreak.	Front	Public	Health.	~~2022;~~10:1023519.

doi:10.3389/fpubh.2022.1023519

~~18~~17. Orsini D, Sartini M, Spagnolo AM, Cristina ML, Martini M. 2024. Mpox: ~~“the~~‘the

stigma is as dangerous as the virus.”’ Historical, social, ethical issues and future

forthcoming. J Prev Med Hyg. ~~2024;~~64(4):~~E398–E404~~E398-E404. doi:10.15167/2421-

4248/jpmh2023.64.4.3144

~~19~~18. Smith AKJ, Storer D, Lancaster K, et al. 2024. Mpox illness narratives: stigmatising

care and recovery during and after an emergency outbreak. Qual Health Res. ~~2024;~~34(12):~~1161–1174~~1161-1174. doi:10.1177/10497323241234482

~~20~~19. Takenaka BP, Kirklewski SJ, Griffith FJ, et al. ~~“It’s~~2024. ‘It’s another gay

~~disease”~~disease’: ~~An~~an intersectional qualitative approach contextualizing the lived

experiences of young gay, bisexual, and other sexual minoritized men in the United States during the mpox outbreak. BMC Public Health. ~~2024;~~24:1574. doi:10.1186/s12889-024-

19062-z

~~21~~20. Witzel TC, Ghobrial A, Palich R, et al. 2024. Experiences of mpox illness and case

management among cis and trans gay, bisexual and other men who have sex with men in England:	a	qualitative	study.	EClinicalMedicine.	~~2024;~~70:102522.

doi:10.1016/j.eclinm.2024.102522

~~22~~21. Biesty CP, Hemingway C, Woolgar J, et al. 2024. Community led health promotion

to counter stigma and increase trust amongst priority populations: lessons from the ~~2022–~~

~~2023~~2022-2023	UK	mpox	outbreak.	BMC	Public	Health.	~~2024;~~24:1638.

doi:10.1186/s12889-024-19142-2

~~23~~22. Logie CH. 2022. What can we learn from HIV, COVID-19 and mpox stigma to

guide stigma-informed pandemic preparedness? J Int AIDS Soc. ~~2022;~~25:e26060.

doi:10.1002/jia2.26060

~~24~~23. Billings J, Ching BCF, Gkofa V, et al. 2021. Experiences of frontline healthcare

workers and their views about support during COVID-19 and previous pandemics: a systematic review and qualitative meta-synthesis. BMC Health Serv Res. ~~2021;~~21(1):923.

doi:10.1186/s12913-021-06917-z

~~25~~24. Apea V, Titanji BK, Dakin FH, et al. 2025. International healthcare workers’

experiences and perceptions of the 2022 multi-country mpox outbreak. PLOS Glob Public Health. ~~2025;~~5(2):e0003704. doi:10.1371/journal.pgph.0003704

~~26~~25. Bow SMA, Schröder-Bäck P, Norcliffe-Brown D, et al. 2023. Moral distress and

injury in the public health professional workforce during the COVID-19 pandemic. J Public Health (Oxf). ~~2023;~~45(3):~~697–705~~697-705. doi:10.1093/pubmed/fdad010

~~27~~26. Jahromi AS, Jokar M, Sharifi N, et al. 2024. Global knowledge and attitudes towards

mpox (monkeypox) among healthcare workers: a systematic review and meta-analysis. Int Health. ~~2024;~~16:487-498. doi:10.1093/inthealth/ihad107

~~28~~27. West CP, Dyrbye LN, Sinsky C, et al. 2020. Resilience and burnout among

**Formatted:** English (United States)

physicians and the general US working population. JAMA Netw Open. ~~2020;~~3(7):e209385. doi:10.1001/jamanetworkopen.2020.9385

~~29~~28. Hussein M, et al. 2024. Ethical considerations during ~~Mpox~~mpox

**Formatted:** French (France)

~~Outbreak~~outbreak: a scoping review. BMC Med Ethics. ~~2024;~~25:78. doi:10.1186/s12910-

024-01080-4

~~30~~29. May T, Towler L, Smith LE, et al. 2023. Mpox knowledge, behaviours and barriers

to public health measures among gay, bisexual and other men who have sex with men in the UK: a qualitative study to inform public health guidance and messaging. BMC Public Health. ~~2023;~~23(1):2265. doi:10.1186/s12889-023-17134-4

~~31~~30. Torres TS, Silva MST, Coutinho C, et al. 2023. Evaluation of ~~Mpox~~mpox

~~Knowledge~~knowledge, ~~Stigma~~stigma, and willingness to ~~Vaccinate~~vaccinate for

~~Mpox~~mpox: cross-sectional web-based survey among sexual and gender minorities. JMIR

Public Health Surveill. ~~2023;~~9:e46489. doi:10.2196/46489

~~32~~31. McLeroy KR, Bibeau D, Steckler A, Glanz K. 1988. An ecological perspective on

health	promotion	programs.	Health	Educ	Q.	~~1988;~~15(4):351-377.

doi:10.1177/109019818801500401

~~33~~32. Gale NK, Heath G, Cameron E, Rashid S, Redwood S. 2013. Using the framework

method for the analysis of qualitative data in multi-disciplinary health research. BMC Med Res Methodol. ~~2013;~~13:117. doi:10.1186/1471-2288-13-117

~~34~~33. Ritchie J, Spencer L. 1994. Qualitative data analysis for applied policy research. In:

Bryman A, Burgess RG, editors. Analyzing qualitative data. London: Routledge; ~~1994.~~ p.

~~173–194~~173-194.

~~35~~34. Pope C, Ziebland S, Mays N. 2000. Qualitative research in health care:

~~Analysing~~analysing	qualitative	data.	BMJ.	~~2000;~~320(7227):114-116.

doi:10.1136/bmj.320.7227.114

~~36~~35. May CR, Johnson M, Finch T. 2016. Implementation, context and complexity.

Implement Sci. ~~2016;~~11:141. doi:10.1186/s13012-016-0506-3

~~37~~36. Tong A, Sainsbury P, Craig J. 2007. Consolidated criteria for reporting qualitative

research (COREQ): a 32-item checklist for interviews and focus groups. Int J Qual Health Care. ~~2007;~~19:349-357. doi:10.1093/intqhc/mzm042

~~38~~37. Schmalzle SA, Grant M, Lovelace S, et al. 2024. Survey of pain and stigma

experiences in people diagnosed with mpox in Baltimore, Maryland during 2022 global outbreak. PLoS ONE. ~~2024;~~19(5):e0299587. doi:10.1371/journal.pone.0299587

~~39~~38. Bury M. 1982. Chronic illness as biographical disruption. Sociol Health Illn.

~~1982;~~4:167-182. doi:10.1111/1467-9566.ep11339939

~~40~~39. Hatzenbuehler ML, Phelan JC, Link BG. 2013. Stigma as a fundamental cause of

population	health	inequalities.	Am	J	Public	Health.	~~2013;~~103:813-821.

doi:10.2105/AJPH.2012.301069

~~41~~40. Paterson A, Cheyne A, Jones B, et al. 2025. An hourglass model for conceptualising

stigma in infectious disease outbreaks. Sci Rep. ~~2025;~~15:15339. doi:10.1038/s41598-025-

98995-w

41. Maslach C, Jackson SE. 1981. The measurement of experienced burnout. J Occup

Behav. 2(2):99-113. doi:10.1002/job.4030020205

42. Jameton A. 1984. Nursing practice: The ethical issues. Englewood Cliffs (NJ):

Prentice-Hall.

43. Epstein EG, Hamric AB. 2009. Moral distress, moral residue, and the crescendo effect.

J Clin Ethics. 20(4):330-342.

44. Litz BT, Stein N, Delaney E, Lebowitz L, Nash WP, Silva C, et al. 2009. Moral injury

and moral repair in war veterans: a preliminary model and intervention strategy. Clin

Psychol Rev. 29(8):695-706. doi:10.1016/j.cpr.2009.07.003

45. Dean W, Talbot SG, Dean A. 2019. Reframing clinician distress: moral injury not

burnout. Fed Pract. 36(9):400-402.

46. Leppold C, Gibbs L, Block K, Reifels L, Quinn P. 2022. Public health implications of

multiple disaster exposures. Lancet Public Health. 7(3):e274-e286. doi:10.1016/S2468-

2667(21)00255-3

47. Newnham EA, Mergelsberg ELP, Chen Y, Kim Y, Gibbs L, Dzidic PL, et al. 2022.

**Formatted:** English (United States)

Long term mental health trajectories after disasters and pandemics: a multilingual

systematic review of prevalence, risk and protective factors. Clin Psychol Rev. 97:102203.

doi:10.1016/j.cpr.2022.102203

48. Li A, Leppold C. 2025. Long-term mental health trajectories across multiple exposures

**Formatted:** French (France)

to climate disasters in Australia: a population-based cohort study. Lancet Public Health.

10(5):e391-e400. doi:10.1016/S2468-2667(25)00068-4

49. Fronteira I, Mathews V, Bandeira Dos Santos RL, Matsumoto K, Amde W, Pereira A,

et al. 2024. Impacts for health and care workers of COVID-19 and other public health

emergencies of international concern: living systematic review, meta-analysis and policy

recommendations. Hum Resour Health. 22(1):10. doi:10.1186/s12960-024-00892-2

~~42~~50. Betsch C, Wieler LH, Habersaat K. 2020. Monitoring behavioural insights related

to COVID-19. Lancet. ~~2020;~~395(10232):1255-1256. doi:10.1016/S0140-6736(20)30729-

7

**Supporting information**

**S1 Appendix. Framework description and thematic framework summary.** Integrated SEM+HSDF analytical framework, S1 Fig thematic framework diagram, and thematic framework overview.

**S2 Appendix. Semi-structured interview guides.** Interview guides for survivors, healthcare workers, community members, and contact tracers.

**S3 Appendix. Focus group discussion guide.** Guide for focus group discussions with healthcare workers, community members, and contact tracers.

**S4 Appendix. Coding framework.** Full codebook with code names, definitions, inclusion/exclusion criteria, and example excerpts.

S5 Appendix. COREQ checklist. Completed the Consolidated Criteria for Reporting

Qualitative Research checklist.

**S6 Appendix. Extended participant quotations by theme.** Additional quotations supporting each theme and subtheme.

S7 Appendix. PLOS Inclusivity in Global Research Questionnaire. Completed

questionnaire describing local leadership, Sierra Leonean authorship, community and

institutional embeddedness, and the contribution of regional qualitative and mental health

expertise.

Response to Reviewers

Response to Reviewers

PLOS Global Public Health | Manuscript ID: PGPH-D-26-00336

Suffering in silence: Stigma, healthcare barriers, and resilience during Sierra Leone’s 2025 clade IIb mpox outbreak—A multi-perspective qualitative study

Date: 8 May 2026

Dear Dr. Bloom and Members of the Editorial Team,

We thank you and the reviewers for the careful consideration given to our manuscript and for the constructive comments. We are grateful that Reviewer 1 found the manuscript well written and methodologically sound, and that Reviewer 2 described the study as strong, well-executed, timely, and methodologically robust.

We revised the manuscript to address each journal requirement and each reviewer comment. In particular, we clarified the relationship between our sensitising framework and inductive findings; strengthened the conceptual framing of compound trauma; clarified the rationale for healthcare worker focus group discussions and the limits of those data; removed the risk of over- interpreting subgroup tables by presenting subgroup observations narratively; added a transparent limitation on sexual transmission and underreporting; expanded Theme 5 on health system response, vaccination, and coordination; and clarified the local leadership and authorship structure of the study. All changes are visible in the revised manuscript with changes highlighted.

# Responses to Journal Requirements

### Journal Requirement 1: Inclusivity in Global Research Questionnaire

#### Response:

We thank the editorial team for this request and for the journal's commitment to transparency, equity, and accountability in global health research. We completed the PLOS Inclusivity in Global Research Questionnaire and uploaded it as Supporting Information. We also revised the manuscript's Research team and reflexivity section and added a brief Methods subsection directing readers to the completed checklist.

This study was conceived, approved, implemented, analysed, and interpreted from within Sierra Leone’s public health system and under the institutional mandate of the National Public Health Agency (NPHA). The study was not organised as an externally led research project; it arose from and remained accountable to Sierra Leone’s national public health response structures.

Page **1** of **7**

The author team includes senior Sierra Leonean public health leadership and surveillance expertise. Dr. Eric Nzirakaindi Ikoona, the corresponding author and first author, works with the NPHA in Freetown, Sierra Leone, as strategic advisor for research and evaluation where he has served for eight years following doctoral training. His post-doctoral public health practice, outbreak response work, and research leadership are embedded within Sierra Leone's national public health system. Professor Foday Sahr is a senior research professor and Executive Director of the NPHA; Dr Mohamed Alex Vandi is a Sierra Leonean physician and Deputy Executive Director of the NPHA; and Mary Magdalene Sinnah is a Sierra Leonean Principal Surveillance Officer. Their roles brought direct knowledge of Sierra Leone's outbreak response system, surveillance architecture, institutional context, and community-facing public health practice.

Dr. Lucy Namulemo was included for her specialist expertise as a PhD-trained researcher in qualitative research and mental health; her role strengthened the methodological and psychosocial dimensions of the study and did not replace local leadership.

All eight research assistants were Sierra Leoneans, embedded in Sierra Leonean cultural contexts, and collectively fluent in the local languages used for the study. They conducted data collection in English, Krio, Mende, or Temne according to participant preference. The study was therefore locally led, institutionally grounded, and accountable to Sierra Leone's public health system and communities.

### Journal Requirement 2: Data Availability

#### Response:

We respectfully request a targeted exemption from public deposition of full interview and focus group transcripts. The transcripts contain sensitive qualitative data from a stigmatising infectious disease outbreak. Even after de-identification, they include indirect identifiers related to geography, household relationships, illness trajectories, health facility encounters, occupational roles, and outbreak response activities. Public release could create a credible risk of deductive identification, especially in districts with small numbers of cases or specialised response roles.

To support transparency and reproducibility, we provide substantial study materials as Supporting Information: interview and focus group guides, the full coding framework with inclusion and exclusion criteria, the completed COREQ checklist, and extended de-identified quotations mapped to themes and subthemes. Additional de-identified excerpts may be requested from the corresponding author under a data-sharing agreement, subject to conditions consistent with Sierra Leone Ethics and Scientific Review Committee approval.

Page **2** of **7**

### Journal Requirement 3: Ethics Statement Placement

#### Response:

We moved the Ethics Statement to the beginning of the Methods section under the subheading “Ethics Statement,” as required. We retained fuller procedural detail later in the Methods section under participant confidentiality and support procedures.

### Journal Requirement 4: Figure Files

#### Response:

We prepared a separate TIFF figure file and processed it through the PLOS NAAS tool. The validated file is submitted with this revision.

### Journal Requirement 5: Title Consistency

#### Response:

We revised the manuscript title to match the title used in the online submission form: “Suffering in silence: Stigma, healthcare barriers, and resilience during Sierra Leone’s 2025 clade IIb mpox outbreak—A multi-perspective qualitative study.”

### Journal Requirement 6: Supporting Information Legends

#### Response:

We added a full list of Supporting Information legends after the References section and removed attachment-placeholder text from the manuscript file.

# Reviewer 1: Dr. Chizaram Onyeaghala

We thank Reviewer 1 for the generous and constructive assessment, and for recognising the

importance of stigma and psychosocial challenges in mpox response. We accepted the reviewer’s tracked edits and corrected the abbreviation, typographical, and wording issues identified in the annotated manuscript. We also reviewed the manuscript for similar errors and corrected repeated definitions of abbreviations after first use, wording duplication, and minor syntax issues.

# Reviewer 2: Anonymous

We thank Reviewer 2 for the careful and intellectually rigorous critique. The six major comments substantially improved the manuscript. We respond to each point below.

### Comment 1: Stigma embedded in the analytical framework

#### Reviewer comment:

Stigma was embedded in the analytical framework prior to data collection but is then presented as an emergent finding. This is not a trivial issue. It should be explicitly acknowledged as a

Page **3** of **7**

limitation. More importantly, the authors should be clear about what did emerge inductively— what findings challenged, extended, or complicated the initial framework?

#### Response:

We agree. We revised the Methods, Results, Discussion, and Limitations sections to distinguish between framework-informed sensitising concepts and inductive findings. The revised manuscript now states that stigma was anticipated by the HSDF and should not be presented as a wholly inductive discovery. What emerged inductively was the recursive movement of stigma across illness experience, care-seeking, professional practice, contact tracing, and system response. We also identified inductive extensions to the framework: institutional stigma directed toward treatment centres, associative stigma toward contact tracers, religious and moral idioms of internalised stigma, informal stigma-mitigation practices used by response teams, and the cyclical relationship between enacted stigma, anticipated rejection, concealment, and community suspicion.

### Comment 2: Compound trauma underdeveloped

#### Reviewer comment:

The concept of “compound trauma” among HCWs with prior Ebola and COVID-19 experience is arguably the most novel contribution of the paper. It is well described in the results and flagged in the discussion, but it is underdeveloped conceptually. The current framing leans on burnout and moral distress literature, which is not sufficient. There is a substantial body of work on cumulative trauma, moral injury, and sequential disaster exposure that is not meaningfully engaged here. If this is intended as a novel empirical contribution it needs a stronger theoretical anchor and a more prominent position in the conclusions.

#### Response:

We agree. We strengthened the conceptual discussion of compound trauma by distinguishing it from burnout and linking it to cumulative trauma, moral injury, and sequential disaster exposure. The revised manuscript now defines compound trauma as the reactivation and accumulation of distress across discrete epidemic events within the same professional life course. We added relevant references on moral injury and disaster mental health and gave the concept a more prominent place in the Conclusions. A companion manuscript examining compound trauma in depth within the Sierra Leone healthcare workforce context is currently in preparation. The present paper stands independently on the conceptual grounding provided in the revised Discussion.

### Comment 3: HCW FGD rationale and silencing dynamics

#### Reviewer comment:

Page **4** of **7**

Survivors were interviewed individually, which is appropriate given the sensitivity and stigma associated with their experiences. However, HCWs participated in both SSIs and FGDs, and the rationale for this is not clearly articulated. FGDs in hierarchical clinical settings raise real concerns about suppressed disclosure. The authors should clarify whether groups were stratified by role or seniority, and whether any silencing dynamics were observed or actively mitigated.

#### Response:

We agree. We revised the Data Collection and Limitations sections. The manuscript now explains that healthcare worker SSIs were used for sensitive individual accounts, including moral distress and emotional burden, while FGDs were used to explore shared operational experiences and collective reflections on service delivery, coordination, and facility-level challenges. We clarified that groups were organised by stakeholder type and district, and that hierarchy was reduced where feasible, but full stratification by cadre or seniority was not always possible because of small eligible staff numbers in some facilities. We also added mitigation steps: facilitators monitored group dynamics, invited quieter participants to speak, normalised disagreement, warned against identifying colleagues or patients, and offered individual follow-up where feasible. We explicitly acknowledge that some suppression of disclosure may still have occurred.

### Comment 4: Subgroup presentation in Tables 2 and 3

#### Reviewer comment:

Tables 2 and 3 present subgroup variation and risk over-interpretation. Some subgroups, such as Kenema district survivors, are too small to support even cautious patterning. The authors should consider whether these tables add sufficient analytical value or whether these observations would be better integrated narratively.

#### Response:

We agree. We removed Tables 2 and 3 from the revised manuscript and integrated the subgroup observations narratively. The revised text now explicitly states that these observations are exploratory analytic prompts, not generalisable subgroup differences. We retained only cautious narrative observations around gender, age, urban/rural residence, employment, and care-seeking context, with clear warnings about small subgroup sizes.

### Comment 5: Sexual transmission and underreporting

#### Reviewer comment:

The paper is notably cautious around sexual transmission, despite genomic and epidemiologic signals and clear stigma tied to perceived sexual exposure. The authors should address whether relevant experiences were likely underreported and discuss this transparently as a limitation.

Page **5** of **7**

#### Response:

We agree. We revised the Introduction and Limitations sections to address this transparently and carefully. The manuscript now states that genital involvement, clade IIb epidemiology, and participant accounts linking mpox to shame, intimacy, and perceived sexual exposure suggest that sexual transmission concerns shaped stigma and disclosure. We added a limitation stating that sexual exposures and partner-related experiences were likely underreported because of stigma, fear of disclosure, and the social risks attached to discussing sexual behaviour in this setting. We make no claims about the actual transmission routes of individual participants.

### Comment 6: Theme 5 underdeveloped

#### Reviewer comment:

Theme 5 (“Building the plane while flying”) is underdeveloped relative to the earlier themes. Health system response, particularly vaccination rollout and coordination challenges, is highly policy-relevant and should be expanded with more granular detail and analytic depth.

#### Response:

We agree. We expanded Theme 5 substantially. The revised section now includes adaptive capacity and local improvisation, coordination and information flow, vaccination rollout and trust, and contact tracing barriers. We added detail on treatment capacity expansion, referral coordination, informal communication networks, bed and transport constraints, data-sharing challenges, vaccination prioritisation, cold-chain and scheduling issues, community uncertainty about eligibility, and how stigma shaped cooperation with contact tracing and vaccination. We also strengthened the policy implications for health system preparedness in post-conflict, multi- epidemic settings.

We trust that these revisions address the editor’s requirements and the reviewers’ comments. We are grateful for the opportunity to revise the manuscript and submit it for further consideration.

Yours sincerely,

Dr. Eric Nzirakaindi Ikoona, MD, MMed, PhD, MPH Corresponding Author

Strategic Adviser for Research and Evaluation, National Public Health Agency Freetown, Sierra Leone

[ikoonae@yahoo.com](mailto:ikoonae@yahoo.com)

ORCID: 0000-0003-3402-1961

Page **6** of **7**

On behalf of Lucy Namulemo, Mary Magdalene Sinnah, Mohamed Alex Vandi, and Foday Sahr

Page **7** of **7**
