# Supplementary material for: Suffering in silence: Stigma, healthcare barriers, and resilience during Sierra Leone’s 2025 clade IIb mpox outbreak—A multi-perspective qualitative study
Source: PLOS Glob Public Health. 2026 Jun 30;6(6):e0006686. doi: 10.1371/journal.pgph.0006686 (PMC13318003; doi:10.1371/journal.pgph.0006686)
Supplement: Attachment — Submitted filename: PGPH-D-26-00336.docx [file pgph.0006686.s008.docx]

PLOS Global Public Health

# Suffering in silence: Stigma, healthcare barriers, and resilience during Sierra Leone's 2025 clade IIb mpox outbreak—A multi-perspective qualitative study

--Manuscript Draft--

| **Manuscript Number:** | PGPH-D-26-00336 |
| --- | --- |
| **Article Type:** | Research Article |
| **Full Title:** | Suffering in silence: Stigma, healthcare barriers, and resilience during Sierra Leone's 2025 clade IIb mpox outbreak—A multi-perspective qualitative study |
| **Short Title:** | Mpox stigma and healthcare experiences in Sierra Leone |
| **Corresponding Author:** | Eric Nzirakaindi Ikoona, MD, MMED, PhD, MPH National Public Health Agency  FREETOWN, SIERRA LEONE |
| **Order of Authors:** | Eric Nzirakaindi Ikoona, MD, MMED, PhD, MPH |
|  | Lucy Namulemo, MSc, PhD |
|  | Mary Magdalene Sinnah, MPH |
|  | Mohamed Alex Vandi, MD, MPH |
|  | Foday Sahr, MBChB, MMED, PhD |
| **Keywords:** | mpox, monkeypox, Sierra Leone, qualitative research, lived experience, stigma, healthcare workers, health systems, outbreak response, clade IIb |
| **Abstract:** | Background  Mpox was confirmed in Sierra Leone in January 2025, with a public health emergency declared shortly thereafter. By June 2025, the country reported over 4,000 confirmed cases caused by clade IIb. Limited qualitative research has explored the lived experiences of affected populations during mpox outbreaks in African settings. This study explored multi-stakeholder experiences of mpox, including illness trajectories, stigma manifestations, healthcare-seeking behaviours, and health system response challenges.  Methods  We conducted a multi-perspective qualitative study using thematic framework analysis between March and August 2025 across four districts. Participants included mpox survivors (n=42), healthcare workers (n=38), community members (n=36), and contact tracers (n=24). Data were collected through 94 semi-structured interviews and 12 focus group discussions. Analysis was guided by a modified Social Ecological Model integrating the Health Stigma and Discrimination Framework.  Results  Five interconnected themes emerged: (1) illness characterised by physical suffering and lasting bodily changes; (2) multi-layered stigma operating across individual, interpersonal, community, and institutional levels; (3) healthcare-seeking shaped by fear of disclosure and economic constraints; (4) healthcare workers’ moral distress and professional isolation; (5) adaptive health system response alongside coordination challenges. Stigma operated as a cross-cutting dimension that intersected with all other themes, shaping illness experience, care-seeking behaviour, professional practice, and system-level response simultaneously.  Conclusions  Stigma permeated all dimensions of the mpox outbreak experience, delaying care- seeking, undermining contact tracing, and compounding healthcare worker distress. Compound trauma from sequential epidemic exposure emerged as a distinct burden among frontline workers. These findings point to the need for stigma-informed outbreak response strategies, sustained healthcare worker support mechanisms, and community-centred communication approaches in post-conflict, resource-constrained settings facing recurrent epidemics. |
| **Additional Information:** |  |

*Powered by Editorial Manager® and ProduXion Manager® from Aries Systems Corporation*

| **Question** | **Response** |
| --- | --- |
| **Financial Disclosure**  Enter a financial disclosure statement that describes the sources of funding for the work included in this submission and the role the funder(s) played. This includes grants and any commercial funding of the work or authors.  This statement will be typeset if the manuscript is accepted for publication.  *Please review the* [*submission guidelines*](http://journals.plos.org/globalpublichealth/s/submission-guidelines#loc-financial-disclosure-statement) *and the instructions link below for detailed requirements and guidance.* | The authors received no specific funding for this work. |
| **Competing Interests**  On behalf of all authors, disclose any competing interests that could be perceived to bias this work.  This statement will be typeset if the manuscript is accepted for publication.  *Please review the instructions link below and PLOS Global Public Health’s* [*competing interests*](https://journals.plos.org/globalpublichealth/s/competing-interests) *policy to determine what information must be disclosed at submission.* | The authors have declared that no competing interests exist. |
| **Data Availability**  Before publication, Authors are required to make fully available and without restriction all data underlying their findings. Please see our [PLOS Data](https://journals.plos.org/globalpublichealth/s/data-availability) [Policy](https://journals.plos.org/globalpublichealth/s/data-availability) page for detailed information on this policy.  A **Data Availability Statement**, detailing where the data can be accessed, is | De-identified qualitative data (full transcripts) cannot be publicly shared because they contain sensitive information and indirect identifiers related to a stigmatizing infectious disease outbreak context, and public release may risk participant re-identification given the small number of mpox cases in specific geographic areas during the study period. The study materials that support transparency and reproducibility are available in the Supporting Information files: interview and focus group discussion guides (S2–S3 Appendix), the full coding framework with inclusion/exclusion criteria (S4 Appendix), the completed COREQ checklist (S5 Appendix), and extended de-identified quotations mapped to themes (S6 Appendix). Researchers may request access to further de- identified data excerpts under a data-sharing agreement, subject to approval by the Sierra Leone Ethics and Scientific Review Committee. Requests should be directed to the corresponding author (Eric Nzirakaindi Ikoona, [ikoonae@yahoo.com)](mailto:ikoonae@yahoo.com) with a brief proposal describing the intended use and data protection safeguards. |

*Powered by Editorial Manager® and ProduXion Manager® from Aries Systems Corporation*

| required at first submission. Insert your Data Availability Statement in the box below.  Please see the [data reporting](https://journals.plos.org/globalpublichealth/s/submission-guidelines#loc-data-reporting) section of our submission guidelines for instructions on what you need to include in your Data Availability Statement.  This statement will be typeset if the manuscript is accepted for publication.  *PLOS allows rare exemptions to address legal and ethical concerns. If you have legal or ethical restrictions, please use the box below to detail these in full sentences for the Journal team to consider.* |  |
| --- | --- |

*Powered by Editorial Manager® and ProduXion Manager® from Aries Systems Corporation*

Manuscript	[Click here to access/download;Manuscript;mpox_PLOS.docx](https://www2.cloud.editorialmanager.com/pgph/download.aspx?id=736742&guid=557064b5-1593-4986-ab6c-cc057f624b4e&scheme=1)[<https://www2.cloud.editorialmanager.com/pgph/download.aspx?id=736742&guid=557064b5-1593-4986-ab6c-cc057f624b4e&scheme=1>](https://www2.cloud.editorialmanager.com/pgph/download.aspx?id=736742&guid=557064b5-1593-4986-ab6c-cc057f624b4e&scheme=1)

### Stigma, healthcare barriers, and resilience during Sierra Leone’s 2025 clade IIb mpox outbreak: A multi-perspective qualitative study

**Short Title: Mpox stigma and healthcare experiences in Sierra Leone**

Eric Nzirakaindi Ikoona¹*, Lucy Namulemo²·³, Mary Magdalene Sinnah¹, Mohamed Alex Vandi¹, Foday Sahr¹

¹ National Public Health Agency, Freetown, Sierra Leone

² Foothills Community-Based Interventions, Kentucky, United States of America

³ Uganda Counselling and Support Services, Kampala, Uganda

* Corresponding author

E-mail: [ikoonae@yahoo.com](mailto:ikoonae@yahoo.com) (ENI)

# Abstract

### Background

Mpox was confirmed in Sierra Leone in January 2025, with a public health emergency declared shortly thereafter. By June 2025, the country reported over 4,000 confirmed cases caused by clade IIb. Limited qualitative research has explored the lived experiences of affected populations during mpox outbreaks in African settings. This study explored multi-stakeholder experiences of mpox, including illness trajectories, stigma manifestations, healthcare-seeking behaviours, and health system response challenges.

### Methods

We conducted a multi-perspective qualitative study using thematic framework analysis between March and August 2025 across four districts. Participants included mpox survivors (n=42), healthcare workers (n=38), community members (n=36), and contact tracers (n=24). Data were collected through 94 semi-structured interviews and 12 focus group discussions. Analysis was guided by a modified Social Ecological Model integrating the Health Stigma and Discrimination Framework.

### Results

Five interconnected themes emerged: (1) illness characterised by physical suffering and lasting bodily changes; (2) multi-layered stigma operating across individual, interpersonal, community, and institutional levels; (3) healthcare-seeking shaped by fear of disclosure and economic constraints; (4) healthcare workers’ moral distress and professional isolation; (5) adaptive health system response alongside coordination challenges. Stigma operated as a cross-cutting dimension that intersected with all other themes, shaping illness experience, care-seeking behaviour, professional practice, and system-level response simultaneously.

### Conclusions

Stigma permeated all dimensions of the mpox outbreak experience, delaying care- seeking, undermining contact tracing, and compounding healthcare worker distress. Compound trauma from sequential epidemic exposure emerged as a distinct burden among frontline workers. These findings point to the need for stigma-informed outbreak response strategies, sustained healthcare worker support mechanisms, and community-

centred communication approaches in post-conflict, resource-constrained settings facing recurrent epidemics.

**Keywords: mpox, monkeypox, Sierra Leone, qualitative research, lived experience, stigma, healthcare workers, health systems, outbreak response, clade IIb**

# Introduction

Mpox (formerly monkeypox) remains a significant public health concern. The World Health Organization (WHO) declared the mpox upsurge a Public Health Emergency of International Concern (PHEIC) on 14 August 2024 [4]. Mpox was first confirmed in Sierra Leone on 10 January 2025, the outbreak was officially declared on 13 January 2025, and a national public health emergency was declared on 16 January 2025 [1,2]. By 22 June 2025, the country reported 4,294 confirmed cases and 28 deaths [3]. In early May 2025, Sierra Leone accounted for approximately half of Africa’s confirmed mpox cases, underscoring the intensity of transmission during the outbreak acceleration phase [5].

The Sierra Leone outbreak presents distinctive epidemiological and social characteristics. Genomic evidence indicates that most sequences fall within clade IIb and cluster as a distinct group descended from lineage A.2.2, consistent with sustained human-to-human transmission; a proposed designation identifies this newly observed Sierra Leone lineage as G.1 (alias of A.2.2.1) [6]. Frequent genital involvement among cases supports the plausibility of sexual networks contributing to clade IIb transmission in this setting [7,8], carrying significant implications for understanding disease spread, designing targeted interventions, and addressing the complex social dimensions of the outbreak.

## The Sierra Leone context

Sierra Leone’s mpox response occurs within a health system shaped by post-conflict rebuilding and the 2014–2016 Ebola epidemic, which disrupted services and workforce capacity [9,10]. More recently, the country navigated the COVID-19 pandemic while simultaneously addressing endemic challenges, including malaria, tuberculosis, and HIV [8]. These cumulative experiences have shaped both the health system’s adaptive capacity and community perceptions of outbreak response, creating a distinctive context in which trust, institutional memory, and resource constraints interact in ways that may differ from settings without comparable histories of sequential health emergencies.

The concentration of cases in the Western Area, encompassing Freetown and surrounding districts, reflects underlying patterns of urbanisation, population density, and healthcare access [3]. By mid-2025, four dedicated treatment centres had opened, a 400-bed facility had become operational in Freetown, and over 43,000 individuals had received vaccination— yet these resources remained insufficient for the scale of the epidemic [3,11].

## Stigma and infectious disease outbreaks

Infectious disease outbreaks have historically been accompanied by stigmatisation of affected individuals and communities [13,14]. Link and Phelan’s foundational conceptualisation describes stigma as occurring when elements of labelling, stereotyping, separation, status loss, and discrimination converge within a context of power imbalance [15]. Disease-related stigma manifests through multiple mechanisms: enacted stigma (actual experiences of discrimination), perceived stigma (expectations of mistreatment),

internalised stigma (acceptance of negative societal attitudes), and associative stigma (stigma experienced by those connected to affected individuals) [16].

The mpox outbreak presents particular stigma challenges given its visible cutaneous manifestations, perceived associations with sexual transmission, and historical framings as an “African disease” [17,18]. Evidence from the 2022 global outbreak documented significant stigma among affected populations, including personalised stigma, negative self-image, and disclosure concerns [19,20]. Healthcare workers have similarly reported fear of contagion and social isolation [21,22]. While these dynamics are well documented in high-income country contexts, significant gaps remain in understanding how mpox- related stigma operates in African settings, where different cultural meanings, health system structures, and prior outbreak experiences may produce distinct stigma configurations [23].

## Healthcare workers' experiences in outbreak response

Healthcare workers (HCWs) occupy a critical yet vulnerable position during infectious disease outbreaks [24]. Research from the 2022 mpox response documented knowledge gaps, with 30% of HCWs having never heard of mpox before the outbreak and over one- quarter initially misdiagnosing cases [25]. HCWs reported inadequate access to vaccines, concerns about occupational exposure, and moral distress related to resource constraints [26]. The psychological toll of outbreak response—compounded for many by the recent COVID-19 pandemic—has contributed to burnout, compassion fatigue, and professional attrition [27,28].

## Gaps in current knowledge

Despite growing epidemiological surveillance of mpox in Africa, qualitative research examining lived experiences remains markedly limited [29,30]. To our knowledge, no published qualitative studies have examined mpox experiences from endemic African settings during the current PHEIC period. Existing qualitative research has predominantly emerged from high-income country contexts, primarily focusing on men who have sex with men populations affected by clade IIb during the 2022 outbreak [19,20,31]. This leaves significant knowledge gaps regarding how mpox illness is experienced in African contexts, the specific manifestations and mechanisms of mpox-related stigma in these settings, and healthcare-seeking behaviours and barriers in resource-constrained environments.

## Study aims and theoretical framework

This study aimed to provide a comprehensive qualitative exploration of mpox outbreak experiences in Sierra Leone, addressing the following research questions: (1) How do individuals who have experienced mpox understand and narrate their illness trajectories?

(2) What forms of stigma emerge during mpox outbreaks, and how do these affect individuals, families, and communities? (3) What factors shape healthcare-seeking behaviours among those affected by or at risk of mpox? (4) How do healthcare workers and public health personnel experience outbreak response? (5) How do experiences vary across different social positions and geographic contexts?

Our analysis was guided by a modified Social Ecological Model (SEM) integrating the Health Stigma and Discrimination Framework (HSDF) [16,32] (S1 Appendix). The SEM

provides a multi-level structure for examining individual, interpersonal, community, organisational, and policy factors that shape health experiences. The HSDF offers a cross- cutting framework for understanding how stigma operates through marking, labelling, separation, status loss, and discrimination within specific contexts. We employed these frameworks as sensitising tools rather than rigid analytical templates, remaining open to findings that might challenge, extend, or complicate the frameworks’ assumptions— particularly regarding the interplay between structural constraints and individual-level processes in a post-conflict, resource-constrained setting.

# Methods

## Study design and period

We conducted a multi-perspective qualitative study using thematic framework analysis to examine lived experiences of the mpox outbreak [33,34]. The study was conducted between 10 March and 28 August 2025, spanning the outbreak’s acceleration, peak (May–June 2025), and initial decline phases. Thematic framework analysis is well-suited for policy-relevant health research involving multiple participant groups, enabling systematic comparison across cases while maintaining analytic transparency [33,35]. Our analytical approach was informed by the Social Ecological Model and the Health Stigma and Discrimination Framework, which provided sensitising concepts for theme development [16,32].

We incorporated multiple stakeholder perspectives to triangulate and develop a comprehensive understanding of the outbreak experience. This multi-perspective approach aligns with calls for qualitative research that moves beyond single stakeholder

views to capture the relational and systemic dimensions of health phenomena [36]. The four participant groups—survivors, healthcare workers, community members, and contact tracers—occupy distinct epistemic positions. Survivors provide experiential knowledge grounded in embodied illness experience; healthcare workers contribute professional and observational accounts shaped by clinical training and institutional roles; community members offer perspectives on social responses and collective meanings; and contact tracers provide operational insights from the interface between surveillance systems and community engagement. We treated these perspectives as complementary but epistemically distinct, and our analytical approach was designed to differentiate between lived experience and professional or observational discourse (see Data analysis). The study followed the Consolidated Criteria for Reporting Qualitative Research (COREQ) guidelines [37] (S5 Appendix).

## Setting

The study was conducted across four districts representing diverse epidemiological and geographic contexts: Western Area Urban (Freetown), Western Area Rural, Bo, and Kenema. These districts were selected based on: (1) case burden, with Western Area accounting for approximately 80% of confirmed cases; (2) geographic diversity, including urban, peri-urban, and rural settings; (3) healthcare infrastructure variation, from tertiary facilities to community health centres; and (4) prior outbreak experience, with all districts having been affected by the 2014–2016 Ebola epidemic [9].

At the time of data collection, the four districts exhibited distinct epidemiological profiles. Western Area Urban reported the highest case concentration, driven by population density and social mixing patterns characteristic of the capital. Western Area

Rural, while geographically proximate, exhibied different transmission dynamics associated with peri-urban settlement patterns and more limited healthcare access. Bo and Kenema districts in the south and east reported lower overall case counts but faced particular challenges related to diagnostic capacity, delayed specimen transport, and greater distances to treatment centres. Across all four districts, cases were predominantly among young adults aged 15–35 years, with male predominance in reported cases [7].

## Participants and sampling

We recruited 140 participants across four stakeholder groups: mpox survivors who had completed isolation (n=42), healthcare workers involved in mpox response (n=38), community members from affected areas (n=36), and contact tracers/surveillance officers (n=24). Purposive sampling with maximum variation was employed to ensure diversity across demographic characteristics (age, gender, education), geographic location (urban/peri-urban/rural), and for survivors, illness severity and time since recovery.

Inclusion criteria for survivors required: confirmed mpox diagnosis (laboratory or clinical), completion of isolation period, age 18 years or older, capacity to provide informed consent, and willingness to discuss experiences. Healthcare worker eligibility required direct involvement in mpox clinical care, laboratory services, or infection prevention and control. Community member eligibility required residence in a community with documented mpox cases. Contact tracers required current or recent employment in mpox surveillance activities.

## Participant recruitment and contact

Recruitment utilised multiple pathways tailored to each stakeholder group. For survivor participants, treatment centre staff at the four designated mpox treatment facilities (Connaught Hospital, 34 Military Hospital, Kenema Government Hospital, and Bo Government Hospital) provided study information sheets to patients nearing discharge. Patients who expressed interest were referred to research assistants, who contacted them via telephone 2–4 weeks post-discharge to confirm eligibility and schedule interviews. Community health workers also identified survivors in their catchment areas who had completed home-based isolation.

Healthcare workers were recruited through facility-based approaches. Research team members visited hospitals, peripheral health units, and laboratories involved in the mpox response to present the study at staff meetings. Interested staff provided contact information and were subsequently contacted to arrange interviews at convenient times. Contact tracers were recruited through District Health Management Teams (DHMTs); surveillance focal persons distributed study information during routine team meetings, and interested personnel were contacted directly by research assistants.

Community members were identified through community leaders and existing community health structures. Community Health Officers introduced the study at community meetings in areas with documented mpox cases. Interested community members provided contact details and were subsequently contacted to arrange participation. We assessed theme stability iteratively within each stakeholder group and district; recruitment stopped when additional interviews did not generate new codes or meaningfully extend existing themes.

## Data collection

Data collection employed semi-structured interviews (SSIs) and focus group discussions (FGDs), with methods matched to participant preferences and topic sensitivity. Individual SSIs were preferred for survivors given the personal and potentially stigmatising nature of illness narratives, while FGDs were used to explore community perspectives and healthcare worker experiences where group dynamics could generate richer discussion.

In total, we conducted 94 SSIs (42 with survivors, 28 with healthcare workers, 12 with community members, 12 with contact tracers) and 12 FGDs (4 with healthcare workers, 6 with community members, 2 with contact tracers). FGDs comprised 6–10 participants each, grouped by stakeholder type and district to enable comfortable discussion among peers with shared experiences.

Interview guides were developed separately for each stakeholder group, informed by the theoretical framework and existing literature (S2 and S3 Appendices). Survivor guides explored illness onset and trajectory, healthcare-seeking experiences, social responses and stigma, coping strategies, and perspectives on recovery. Healthcare worker guides examined clinical experiences, occupational challenges, resource constraints, emotional impacts, and recommendations. Community member guides explored knowledge and perceptions, community responses, fears and concerns, and recommendations. Contact tracer guides examined work experiences, challenges encountered, community responses, and support needs.

All guides were piloted with five individuals from each stakeholder group and refined based on feedback. Interviews were conducted in Krio, Mende, Temne, or English according to participant preference by trained research assistants fluent in these

languages. Interviews averaged 62 minutes for survivors (range: 45–95 minutes), 48 minutes for other SSIs (range: 35–75 minutes), and 87 minutes for FGDs (range: 72–110 minutes). All sessions were audio-recorded with permission and transcribed verbatim in the original language before being translated into English by bilingual team members.

Translations were verified by back-translating randomly selected segments (20% of transcripts).

## Informed consent process

The informed consent process was designed to ensure voluntary, informed participation while being sensitive to the stigmatising nature of mpox. Prior to each interview or FGD, research assistants met with potential participants in a private setting of their choosing (typically their home, a private room at a health facility, or a community centre).

Research assistants explained the study using a standardised information sheet available in English, Krio, Mende, and Temne.

The consent process covered: (1) study purpose and procedures, including the expected duration and topics to be discussed; (2) voluntary nature of participation, with explicit emphasis that declining would not affect access to healthcare or other services; (3) right to withdraw at any time without penalty; (4) right to skip any question or stop the interview at any time; (5) confidentiality protections, including use of pseudonyms, secure data storage, and removal of identifying information; (6) potential risks including emotional distress when discussing difficult experiences; (7) available support including referral to mental health services if needed; (8) audio-recording procedures and data storage; (9) plans for data use and dissemination; and (10) contact information for the research team and ethics committee for questions or concerns.

Participants were given time to ask questions and were provided with the information sheet to keep. For participants who could not read, the information sheet was read aloud in their preferred language. Written informed consent was obtained from all participants before data collection commenced. For participants who could not write, witnessed thumbprint consent was obtained in accordance with Sierra Leone Ethics and Scientific Review Committee guidelines. A copy of the signed consent form was provided to each participant.

## Research team and reflexivity

The research team comprised investigators with backgrounds in public health, medicine, nursing, medical anthropology, and community health. The principal investigator (ENI) is a physician and public health researcher based in Sierra Leone with extensive experience in qualitative health research and implementation science. The co-investigator (LN) is a social scientist with training in qualitative methods and prior experience in HIV and infectious disease research in East and West Africa. Research assistants (n=8) were recruited from Sierra Leone; all held at minimum a bachelor’s degree in a health-related field, and four had prior experience in qualitative data collection during the Ebola response. Research assistants received five days of training covering qualitative methods, ethical considerations, sensitive interviewing, emotional support provision, and mpox- specific knowledge. The team included members with prior experience in Ebola response research, providing valuable contextual understanding [12,13].

We maintained reflexive practices throughout, recognising that researchers’ positions— as healthcare professionals, public health practitioners, and Sierra Leoneans navigating the same outbreak—shaped data collection and interpretation. Weekly team debriefings

addressed emerging insights, analytic decisions, and researcher well-being. Reflexive memos documented team members’ evolving understandings, emotional responses, and methodological considerations.

## Data analysis

Analysis followed the Framework Method, enabling systematic cross-case comparison and transparent matrix-based synthesis [33,34]. Analysis proceeded through six phases:

(1) Familiarisation: Analysts read complete transcripts multiple times, noting initial observations; (2) Developing an analytical framework: Initial coding of a subset of transcripts to develop a working codebook informed by the SEM/HSDF theoretical framework and emergent concepts; (3) Indexing: Systematic coding of all transcripts using the agreed codebook; (4) Charting: Summarising coded data into framework matrices organised by theme and participant group; (5) Cross-case analysis: Identifying patterns and variations across participants within and between stakeholder groups; (6) Interpretation: Developing higher-order interpretations connecting findings to broader literature and implications.

A critical analytical decision concerned how to integrate and differentiate perspectives across the four participant groups. We constructed separate framework matrices for each stakeholder group before conducting cross-group analysis, ensuring that patterns within each group were identified before comparisons were drawn. In our interpretive process, we explicitly distinguished between first-person experiential accounts (primarily from survivors), professional observational accounts (primarily from healthcare workers and contact tracers), and community normative perspectives (primarily from community members). Where healthcare workers or contact tracers spoke about patient experiences,

we treated these as institutionally situated observations rather than direct evidence of lived experience, and we note this distinction in our presentation of findings.

Convergence across groups was interpreted as strengthening credibility, while divergence prompted further analytical interrogation of the epistemic basis for differing accounts.

Data management was conducted using NVivo 14 (Lumivero, 2023). Coding was led by ENI and LN, both of whom had doctoral-level training in qualitative research methods. ENI is based in Sierra Leone and brought contextual knowledge of the health system and outbreak response; LN brought a comparative perspective from qualitative research experience in East African settings. A team-based approach to coding was employed, with initial independent coding by both analysts followed by consensus discussions to resolve discrepancies. An audit trail documented all analytic decisions (S4 Appendix). Subgroup analyses examined variations by gender, age group (18–24, 25–34, 35–49, 50+), district, urban/rural residence, education level, and for healthcare workers, professional role and facility type. Given the qualitative nature of the study and modest subgroup sizes, we report observed patterns rather than statistically tested differences, and we exercise caution in interpretation where cell sizes are small. We also attended to how social positions may intersect—for example, how gender and rural residence jointly shaped stigma experiences—rather than treating demographic categories as independent, parallel variables. Member checking was conducted with 20 participants (5 from each stakeholder group) who reviewed preliminary findings and provided feedback that was incorporated into the final analysis.

## Ethical considerations

Ethical approval was obtained from the Sierra Leone Ethics and Scientific Review Committee (SLESRC-2025-049) on 28 February 2025. Given the potentially stigmatising nature of mpox, particular attention was paid to confidentiality protections: participants selected interview locations that offered privacy; identifiers were removed during transcription; quotes are presented using pseudonyms and with identifying details altered; and data files are stored on encrypted, password-protected servers accessible only to the research team.

Emotional distress protocols were established, given the sensitive subject matter. Research assistants were trained to recognise distress signals and offer supportive responses. Referral pathways to mental health services were established in each district through a partnership with Partners in Health Sierra Leone. Participants were informed that they could pause, skip questions, or terminate interviews at any time. Post-interview check-ins were conducted via telephone within one week for survivor participants.

Participants received transportation reimbursement (NLE 50, approximately USD 2.50 at the June 2025 exchange rate) and refreshments, but no other compensation, to minimise coercion.

# Results

## Participant characteristics

Table 1 presents participant demographics. Among the 42 mpox survivors, 26 (61.9%) were male and 16 (38.1%) were female, with a mean age of 28.4 years (range: 18–52). The majority resided in Western Area Urban (n=25, 59.5%), followed by Western Area

Rural (n=9, 21.4%), Bo (n=5, 11.9%), and Kenema (n=3, 7.1%). Time since recovery ranged from 2–14 weeks at interview. Among healthcare workers (n=38), 22 (57.9%) were nurses, 8 (21.1%) were physicians, 5 (13.2%) were laboratory technicians, and 3 (7.9%) were IPC officers. Community members (n=36) were distributed across all four districts with deliberate oversampling from communities with the highest case burdens.

Contact tracers (n=24) represented all District Health Management Teams involved in mpox response.

### Table 1. Demographic characteristics of study participants.

| **Characteristic** | **Survivors (n=42)** | **HCWs (n=38)** | **Community**  **(n=36)** | **Contact Tracers**  **(n=24)** |
| --- | --- | --- | --- | --- |
| Gender – Male | 26 (61.9%) | 15 (39.5%) | 18 (50.0%) | 16 (66.7%) |
| Gender – Female | 16 (38.1%) | 23 (60.5%) | 18 (50.0%) | 8 (33.3%) |
| Age (mean, range) | 28.4 (18–52) | 36.2 (24–58) | 38.7 (22–65) | 32.1 (23–48) |
| Western Area  Urban | 25 (59.5%) | 22 (57.9%) | 14 (38.9%) | 10 (41.7%) |
| Western Area  Rural | 9 (21.4%) | 8 (21.1%) | 10 (27.8%) | 6 (25.0%) |
| Bo District | 5 (11.9%) | 5 (13.2%) | 6 (16.7%) | 4 (16.7%) |
| Kenema District | 3 (7.1%) | 3 (7.9%) | 6 (16.7%) | 4 (16.7%) |

Analysis identified five overarching themes with multiple subthemes, summarised in the thematic framework (S1 Fig), with the full diagram and explanation provided in S1 Appendix. Stigma appeared across all five themes. We present each theme with illustrative quotations, indicating participant characteristics using codes (e.g., S-M-28-

WAU = survivor, male, age 28, Western Area Urban). Extended quotations supporting each theme and subtheme appear in S6 Appendix.

**S1 Fig. Thematic framework showing five overarching themes and their interconnections.** *The full framework diagram and explanation are provided in S1 Appendix.*

## Theme 1: “The body that betrays”—Illness experiences

Survivors described illness experiences characterised by physical suffering, diagnostic uncertainty, and lasting bodily changes. This theme encompassed four subthemes: onset and recognition, physical suffering, diagnostic journeys, and altered body experience.

### Onset and recognition

Initial symptom recognition was frequently delayed or misattributed, particularly in the outbreak’s early phases. Many survivors described initially interpreting symptoms through familiar illness categories—malaria, skin infections, or sexually transmitted infections:

*“I first felt the fever coming and thought, this is malaria again. I took the [antimalarial] tablets and waited. When the bumps started appearing on my skin, I thought maybe it was an allergic reaction. It was only when my friend told me about mpox being in Freetown that I started to worry it could be that.” (S-M-24- WAU)*

### Physical suffering

Survivors described intense physical suffering that often exceeded their expectations. Pain was a dominant feature, particularly associated with lesions, lymphadenopathy, and systemic symptoms. Survivors frequently reported limited access to effective pain relief (S6 Appendix):

*“The pain was like nothing I have experienced before. Every lesion felt like fire burning on my skin. I could not sleep, I could not eat, and I could not even lie down comfortably. The lymph nodes under my arms were so swollen that I could not lower my arms to my sides. I thought I was going to die.” (S-M-35-WAU)*

### Diagnostic journeys

Pathways to diagnosis were often circuitous, involving multiple healthcare encounters. Several survivors described initial misdiagnosis:

*“The first clinic I went to, they said it was chickenpox and gave me calamine lotion. After two days, I went back because it was getting worse, and they said maybe it was syphilis and gave me injections. It was only when I went to [government hospital] that they finally said it could be mpox and took the swab.” (S-M-29-WAU)*

### Altered body experience

Beyond acute illness, survivors described lasting changes in their relationship with their bodies, including scarring and impacts on sexuality and intimacy:

*“Since I recovered, I have not been able to be intimate with my husband. Every time I think about it, I remember the sores in that area, and I feel afraid and*

*ashamed. He is patient with me, but I can see he is frustrated. I do not know if this feeling will ever go away.” (S-F-34-WAU)*

## Theme 2: “Marked and marginalised”—Multi-layered stigma

Stigma emerged across all participant groups and study sites. It operated at multiple levels and through various mechanisms. Four interconnected subthemes were identified: enacted stigma and discrimination, perceived stigma and anticipated rejection, internalised stigma and self-blame, and associative stigma (see S6 Appendix).

### Enacted stigma and discrimination

Survivors reported direct experiences of discrimination from multiple sources, including family rejection, community avoidance, and healthcare discrimination:

*“When my family found out I had mpox, my own brother told me I could not stay in the house anymore. He said I would infect his children. I had to go and stay with a friend who was the only one willing to take me in.” (S-M-26-WAR)*

### Perceived stigma and anticipated rejection

Perceived stigma shaped behaviour, particularly around disclosure:

*“I told no one except my mother and one close friend. Not even my employer—I said I had malaria and needed rest. I was so afraid that if people knew, I would lose everything.” (S-F-27-BO)*

### Internalised stigma and self-blame

Many survivors described accepting negative social messages, leading to shame and self- blame, particularly when illness was attributed to sexual behaviour:

*“I kept asking myself, what did I do to deserve this? I felt so dirty, so ashamed. I thought maybe God was punishing me for something I had done. I could not even look at myself in the mirror.” (S-M-25-WAU)*

### Associative stigma

Stigma extended to families, caregivers, and those associated with outbreak response. Both survivors and healthcare workers described this pattern. Healthcare workers reported being avoided by neighbours and community members because of their roles:

*“My neighbours know I work at the mpox treatment centre. Some of them have started avoiding me. One woman actually told me she does not want me coming near her children because I might bring the disease to the community. It is hurtful because I am trying to help people.” (HCW-F-34-WAU-Nurse)*

## Theme 3: “Seeking care in silence”—Healthcare-seeking trajectories

Multiple factors influenced healthcare-seeking behaviours, including stigma, knowledge, economic constraints, and health system accessibility. Three subthemes characterised these trajectories: delays and barriers, alternative care pathways, and facilitators of timely care (S6 Appendix).

### Delays and barriers

Fear of stigma was the most frequently cited reason for delayed care-seeking:

*“I waited because I was afraid of what would happen if people found out. I thought maybe if I just stayed home, it would go away on its own, and no one would ever need to know.” (S-M-22-WAU)*

Patterns in care-seeking delays varied across participant subgroups. Table 2 summarises the barriers most frequently described by each group. We note that subgroup sizes are modest, and these comparisons illustrate qualitative variation rather than generalisable differences.

### Table 2. Observed patterns in healthcare-seeking delays by subgroup characteristics.

| **Subgroup** | **Key barriers described by participants** |
| --- | --- |
| Urban residents | Fear of disclosure, work concerns, elaborate  concealment strategies |
| Rural residents | Distance to facilities, transport costs, and limited  diagnostic capacity |
| Higher education | Disclosure concerns, workplace stigma, awareness  of long-term implications |
| Lower education | Limited symptom knowledge, financial  constraints, reliance on familiar illness categories |
| Formal employment | Fear of job loss, disclosure to employer,  navigating sick leave |
| Informal employment | Income loss from missed work, no sick leave  provisions, competing livelihood priorities |

**Facilitators of timely care**

Survivors who sought care promptly described factors that facilitated their decision, including awareness of mpox symptoms from public health messaging, social support, and prior positive healthcare experiences:

*“I had seen the messages on the radio about mpox and what signs to look for. When I started having those exact symptoms, I knew I should go get tested right away.” (S-M-33-WAU)*

## Theme 4: “Working at the edge”—Healthcare worker experiences

Healthcare workers described experiences of responding to the outbreak while navigating resource constraints, occupational risks, emotional burdens, and community pressures.

Four subthemes captured these experiences (S6 Appendix).

### Clinical challenges and moral distress

Healthcare workers described confronting clinical situations for which they felt inadequately prepared:

*“There were times when we had more patients than beds. We had to make difficult decisions about who could be admitted and who had to go home. Some patients we sent home were very sick, but we had no space for them. I would lie awake at night wondering if I had made the right choice.” (HCW-M-38-WAU- Physician)*

### Emotional toll and compound trauma

The emotional burden was substantial. Many healthcare workers who had served during previous epidemics described the mpox response reactivating earlier distress:

*“When mpox started spreading, it brought back all those memories from Ebola. The fear, the death, watching colleagues get sick. I thought I had processed all of*

*that but it came flooding back. Some nights I could not sleep.” (HCW-F-45-WAU- Nurse)*

## Theme 5: “Building the plane while flying”—Health system response

The final theme addresses the health system response, drawing primarily on accounts from healthcare workers and contact tracers. Subthemes included adaptive capacity and innovation, coordination challenges, contact tracing barriers, and vaccination experiences (S6 Appendix).

### Contact tracing barriers

Contact tracing was described as particularly challenging, with stigma undermining cooperation:

*“Many patients are reluctant to name their contacts, especially if the contact was through sexual activity. They are afraid of what will happen to those people, or*

*they do not want to reveal their private behaviour.” (CT-M-34-WAU)*

## Subgroup variations

Table 3 summarises variations observed across subgroups. Gender differences were notable: female survivors more frequently described internalised stigma connected to perceived sexual transmission implications, while male survivors more often described economic impacts from work disruption. These gendered patterns were not uniform— younger women in urban areas also described economic concerns similar to those raised by male participants.

Rural participants generally described longer pathways to care, though this varied with proximity to treatment centres and prior health system engagement. Urban participants described more elaborate concealment strategies. We present these observations as descriptive patterns from a modest sample rather than definitive subgroup differences. **Table 3. Summary of observed subgroup variations in key findings.**

| **Subgroup** | **Key observations** |
| --- | --- |
| Female survivors | More frequently described internalised stigma;  concerns about perceived sexual transmission implications |
| Male survivors | Greater emphasis on economic impacts and work  disruption |
| Age 18–24 | More peer-level stigma described; social media disclosure fears; economic vulnerability among  those in informal work |
| Age 50+ | More family-based discrimination is described; intergenerational tensions; compounded by pre-  existing health concerns |
| Rural residence | Longer care-seeking pathways described; limited facility access; varied with proximity to treatment  Centres |
| Urban residence | More elaborate concealment strategies are described;  workplace concerns |

# Discussion

This study provides an in-depth qualitative exploration of mpox outbreak experiences in Sierra Leone, revealing the physical suffering, multi-layered stigma, healthcare-seeking challenges, and health system response dynamics characterising the clade IIb epidemic. While certain themes—such as disease-related stigma, healthcare-seeking delays, and healthcare worker moral distress—resonate with prior research on infectious disease outbreaks, our findings contribute novel insights in several respects that extend beyond contextual confirmation of known patterns [29,30].

Three contributions merit particular emphasis. First, this study provides what is, to our knowledge, the first qualitative account of mpox illness experiences from an African setting during the current PHEIC, offering empirical grounding for understanding how mpox is experienced in a context characterised by post-conflict health system fragility, sequential epidemic exposure, and cultural frameworks distinct from those documented in high-income country studies of the 2022 outbreak. Second, our multi-perspective design reveals how stigma operates not merely as a barrier at discrete points in the care pathway but as a pervasive structural force that simultaneously shapes illness experience, healthcare-seeking, professional practice, and system-level response—a cross-cutting dynamic that existing frameworks may inadequately capture. Third, the phenomenon of compound trauma among healthcare workers—where mpox response reactivated unresolved distress from Ebola and COVID-19 experiences—represents a distinctive finding with implications for workforce sustainability in settings with histories of sequential health emergencies.

## Illness experiences and disrupted embodiment

Survivors’ narratives of physical suffering align with emerging clinical literature documenting severe pain as a hallmark of mpox, particularly when genital lesions are present [7,38]. Our qualitative findings add experiential depth to these clinical observations, revealing how inadequate pain management compounded suffering and eroded trust in healthcare. The phenomenon of “disrupted embodiment”—fundamentally altered relationships with one’s own body—has been documented in other illness contexts but has not previously been explored in relation to mpox [39]. Visible scarring and impacts on sexuality represent lasting consequences extending well beyond acute illness resolution. It should be noted, however, that our cross-sectional design captures experiences at a single point in recovery; longitudinal research would be needed to understand how embodied experiences evolve over time and whether the disruptions described here are enduring or gradually resolve [19].

## Stigma mechanisms and impacts

Our findings extend the Health Stigma and Discrimination Framework to the mpox context, documenting how stigma operates through multiple mechanisms—enacted, perceived, internalised, and associative—at multiple levels from individual to institutional [16]. The visible nature of mpox lesions, coupled with perceived associations with sexual transmission, creates a particularly stigmatising configuration [17,40].

Comparisons to HIV disease were explicit in participant accounts, echoing patterns documented in the 2022 global outbreak [19,20,23].

Critically, our findings illustrate how stigma undermined outbreak control in this setting. Fear of stigma delayed care-seeking, enabling ongoing transmission. Anticipated discrimination reduced disclosure to contacts, hampering contact tracing. Internalised stigma affected mental health and recovery. These pathways indicate that stigma warrants attention not only as a social harm but as a public health concern integral to outbreak response effectiveness [13,23,41].

## Theoretical implications

Our findings have implications for the theoretical frameworks that guided the analysis. The Social Ecological Model provided a useful heuristic for organising multi-level findings, but the empirical data suggest that the relationships between levels are more dynamic and recursive than the model’s nested structure implies. Stigma, in particular, did not operate as a discrete factor at any single level but functioned as a cross-cutting structural force that simultaneously shaped individual cognition, interpersonal relationships, community norms, and institutional practices. This finding suggests that the SEM’s conventional representation of levels as relatively independent layers may need to be adapted for outbreak contexts, where a single social process—stigma—permeates and connects all levels simultaneously.

Similarly, the Health Stigma and Discrimination Framework proved valuable for categorising stigma mechanisms, but our findings suggest areas for extension. The framework’s typology of enacted, perceived, internalised, and associative stigma captured important dimensions, yet participants’ accounts revealed that these mechanisms operated in reinforcing cycles rather than as parallel categories. Enacted stigma fuelled perceived stigma, which in turn drove concealment behaviours that paradoxically

intensified community suspicion and further enacted stigma. This cyclical dynamic, while implied by the framework, deserves more explicit theorisation, particularly in outbreak contexts where the temporal compression of an epidemic may accelerate stigma cycles in ways not fully captured by frameworks developed for chronic conditions such as HIV. Future research might explore how the HSDF could be adapted to account for the rapid onset, acute visibility, and epidemic tempo of mpox-related stigma compared to the more gradual stigma trajectories characteristic of chronic infectious diseases [41].

## Healthcare workers’ experiences

Healthcare workers’ narratives of moral distress, occupational risk anxiety, and emotional burden align with documented experiences from other outbreaks while revealing mpox- specific dimensions [25,26,27]. The compound effect of sequential outbreak response— many participants had served during both Ebola and COVID-19—suggests cumulative workforce impacts that warrant attention [9,28]. Moral distress arising from resource constraints represents a systemic issue requiring health system strengthening, not merely individual-level support [24]. We note that healthcare worker accounts in this study primarily reflect the perspectives of those who remained in their posts during the outbreak; the experiences of those who left clinical practice or were unable to continue responding remain undocumented and may differ substantially.

## Implications for practice and policy

Our findings suggest several implications for mpox response in Sierra Leone and potentially in similar settings, though we note that these are indicative rather than

prescriptive, and their applicability will depend on local context and implementation considerations.

First, the pervasive role of stigma across all domains of the outbreak experience suggests that stigma-informed strategies merit integration into outbreak management frameworks from the outset, rather than being treated as secondary concerns [23,41]. This could include attention to language used in public communications, protection of patient confidentiality, community engagement to address misinformation and fear, and support for affected individuals facing discrimination [42]. The specific forms that such strategies might take require further development and evaluation.

Second, healthcare worker accounts suggest that support mechanisms—including adequate PPE and access to vaccination, psychosocial support, strategies to address associative stigma, and career protections for outbreak responders—may warrant being established as integral components of the response rather than as afterthoughts [26,27]. The compound trauma observed among workers with prior epidemic experience further suggests the potential value of continuity in workforce wellbeing programmes across successive outbreaks.

Third, the care-seeking patterns observed in this study suggest that community-centred communication approaches—emphasising symptom recognition and care availability, delivered through trusted community channels—may facilitate timely care-seeking [41,42]. However, our data capture what participants reported about their care-seeking experiences and decisions, and social desirability considerations may have shaped these accounts. Intervention research would be needed to evaluate the effectiveness of specific communication strategies.

## Limitations

Several limitations should be acknowledged, some of which have already been noted in the discussion of specific findings. First, our sample, while purposively diverse, may not represent all experiences—particularly those who never accessed healthcare or who experienced severe outcomes. Survivor participants, by definition, had recovered sufficiently to participate, potentially excluding the most severe cases. Second, recall bias may affect accounts of illness trajectories, particularly given the emotional intensity of the experiences described. Third, social desirability bias may have influenced responses across all participant groups: survivors may have understated risky behaviours, community members may have moderated stigmatising attitudes, and healthcare workers may have emphasised challenges in advocating for resources. Fourth, the geographic concentration of cases in the Western Area may limit the transferability of findings to districts with different epidemiological and social profiles.

Fifth, the cross-sectional nature of data collection means that our findings capture experiences at particular points in the outbreak trajectory and in individual recovery processes; how these experiences evolve over time—particularly regarding stigma, embodiment, and healthcare worker wellbeing—remains an important question for longitudinal research. Sixth, the absence of male partner perspectives, family members beyond the survivors themselves, and individuals who declined healthcare limits the comprehensiveness of our account of the relational dimensions of mpox experience.

Despite these limitations, our multi-perspective approach, diverse sample, and rigorous analytic procedures support the credibility of findings within the scope of what the study was designed to address.

# Conclusions

Stigma was the dominant social force shaping the mpox outbreak experience in Sierra Leone. It delayed care-seeking, undermined contact tracing, eroded trust in health services, and compounded the emotional burden on healthcare workers—all of which directly compromised outbreak control. These findings indicate that stigma is not a peripheral social concern but a central obstacle to effective epidemic response.

Healthcare workers with prior Ebola and COVID-19 experience carried a compound trauma burden that current support systems did not address. Their accounts point to a cumulative workforce crisis that risks deepening with each successive epidemic unless sustained psychosocial support and career protections are built into response architectures.

Three priorities emerge for outbreak preparedness in similar post-conflict, resource- constrained settings: (1) stigma-informed strategies integrated from the outset of outbreak response, not appended later; (2) healthcare worker support systems that extend across successive outbreaks rather than being rebuilt each time; and (3) community-centred communication that reaches people before stigma drives them into silence. These priorities require further evaluation, but the experiences documented here—from those who lived through the outbreak, treated its patients, and traced its spread—make it clear that technical response alone is insufficient without attention to the social dimensions of epidemic disease.

# Acknowledgments

We extend our profound gratitude to all study participants who shared their experiences during an extraordinarily difficult time. We thank the National Public Health Agency leadership for institutional support; the District Health Management Teams of Western Area Urban, Western Area Rural, Bo, and Kenema for facilitating fieldwork; the research assistants who conducted data collection with sensitivity and skill; and the transcriptionists and translators who enabled accurate representation of participants’ words. We acknowledge technical input from Partners in Health Sierra Leone for mental health referral support.

# Author contributions

**Conceptualization:** Eric Nzirakaindi Ikoona.

**Data curation:** Eric Nzirakaindi Ikoona, Lucy Namulemo.

**Formal analysis:** Eric Nzirakaindi Ikoona, Lucy Namulemo.

**Investigation:** Eric Nzirakaindi Ikoona, Lucy Namulemo, Mary Magdalene Sinnah.

**Methodology:** Eric Nzirakaindi Ikoona, Mohamed Vandi.

**Project administration:** Eric Nzirakaindi Ikoona.

**Supervision:** Foday Sahr.

**Writing – original draft:** Eric Nzirakaindi Ikoona.

**Writing – review & editing:** Eric Nzirakaindi Ikoona, Lucy Namulemo, Mary Magdalene Sinnah, Mohamed Alex Vandi, Foday Sahr.

# Funding

The authors received no specific funding for this work.

# Competing interests

The authors have declared that no competing interests exist.

# Data availability statement

De-identified qualitative data (full transcripts) cannot be shared publicly because they contain sensitive information and carry a risk of participant re-identification. Researchers can request access by contacting the corresponding author [(ikoonae@yahoo.com)](mailto:(ikoonae@yahoo.com) with a brief proposal describing the intended use, data protection safeguards, and an ethics- compliant data management plan. Access is subject to approval conditions consistent with the ethics clearance for this study and may require a data sharing agreement.

# Ethics statement

This study was approved by the Sierra Leone Ethics and Scientific Review Committee (SLESRC-2025-049) on 28 February 2025. All participants provided written informed consent prior to participation.

# References

1. Ministry of Health (Sierra Leone). Mpox declared a public health emergency in Sierra Leone. 2025 Jan 16 [cited 2026 Jan 26]. Available from: https://mohs.gov.sl/mpox- declared-a-public-health-emergency-in-sierra-leone/
2. UNICEF Sierra Leone. Fighting Mpox: UNICEF scales up critical support in Sierra Leone. 2025 Jun 18 [cited 2026 Jan 26]. Available from: https:/[/www.unicef.org/sierraleone/press-releases/fighting-mpox-unicef-scales-critical-](http://www.unicef.org/sierraleone/press-releases/fighting-mpox-unicef-scales-critical-) support-sierra-leone
3. World Health Organization. Multi-country outbreak of mpox, external situation report #54 – 27 June 2025. 2025 Jun 27 [cited 2026 Jan 26]. Available from: https:/[/www.who.int/publications/m/item/multi-country-outbreak-of-mpox--external-](http://www.who.int/publications/m/item/multi-country-outbreak-of-mpox--external-) situation-report--54	27-june-2025
4. World Health Organization. WHO Director-General declares mpox outbreak a public health emergency of international concern. 2024 Aug 14 [cited 2026 Jan 26]. Available from: https:/[/www.who.int/news/item/14-08-2024-who-director-general-declares-mpox-](http://www.who.int/news/item/14-08-2024-who-director-general-declares-mpox-) outbreak-a-public-health-emergency-of-international-concern
5. Africa Centres for Disease Control and Prevention. Sierra Leone fights mpox. 2025 May 18 [cited 2026 Jan 26]. Available from: https://africacdc.org/news-item/sierra-leone- fights-mpox/
6. Sandi JD, Grant DS, Sahr F, Harding A. Genomic epidemiology of Mpox virus in Sierra Leone. Virological [Internet]. 2025 May 28 [cited 2026 Jan 26]. Available from: https://virological.org/t/genomic-epidemiology-of-mpox-virus-in-sierra-leone/995
7. Kangbai JB, Saidu E, Foday IK, et al. Clinical and epidemiological characteristics among probable and confirmed patients with mpox in Sierra Leone reported from January to May 2025. medRxiv. 2025 [cited 2025 Aug 30]. doi:10.1101/2025.05.30.25328691
8. Mathiang JR, Sahr F, Kamara IF, Ansumana R, Rogers MK, Lee SS, et al. Rapid surge of mpox in Sierra Leone: Public health challenges. Int J Infect Dis. 2025;159:107987. doi:10.1016/j.ijid.2025.107987
9. Raven J, Wurie H, Witter S. Health workers’ experiences of coping with the Ebola epidemic in Sierra Leone’s health system: a qualitative study. BMC Health Serv Res. 2018;18:251. doi:10.1186/s12913-018-3072-3
10. Bah AJ, James PB, Bah N, et al. Prevalence of anxiety, depression, and post-traumatic stress disorder among Ebola survivors in northern Sierra Leone: a cross-sectional study. BMC Public Health. 2020;20:1391. doi:10.1186/s12889-020-09507-6
11. Médecins Sans Frontières. Responding to an mpox outbreak in the heart of Sierra Leone. MSF; 2025 Jul 8 [cited 2025 Aug 30]. Available from: https:/[/www.msf.org/responding-mpox-outbreak-heart-sierra-leone](http://www.msf.org/responding-mpox-outbreak-heart-sierra-leone)
12. Kupferschmidt K. Explosive mpox outbreak in Sierra Leone overwhelms health systems. Science. 2025 Jun 2 [cited 2025 Aug 30]. Available from: https:/[/www.science.org/content/article/explosive-mpox-outbreak-sierra-leone-](http://www.science.org/content/article/explosive-mpox-outbreak-sierra-leone-) overwhelms-health-systems
13. Fischer LS, Mansergh G, Lynch J, Santibanez S. Addressing disease-related stigma during infectious disease outbreaks. Disaster Med Public Health Prep. 2019;13:989-994. doi:10.1017/dmp.2018.157
14. Paterson A, Olliaro P, Rojek A. Addressing stigma in infectious disease outbreaks: a crucial step in pandemic preparedness. Front Public Health. 2023;11:1303679. doi:10.3389/fpubh.2023.1303679
15. Link BG, Phelan JC. Conceptualizing stigma. Annu Rev Sociol. 2001;27:363-385. doi:10.1146/annurev.soc.27.1.363
16. Stangl AL, Earnshaw VA, Logie CH, et al. The health stigma and discrimination framework: a global, crosscutting framework to inform research, intervention development, and policy on health-related stigmas. BMC Med. 2019;17:31. doi:10.1186/s12916-019-1271-3
17. Sah R, Mohanty A, Reda A, Padhi BK, Rodriguez-Morales AJ. Stigma during monkeypox outbreak. Front Public Health. 2022;10:1023519. doi:10.3389/fpubh.2022.1023519
18. Orsini D, Sartini M, Spagnolo AM, Cristina ML, Martini M. Mpox: “the stigma is as dangerous as the virus.” Historical, social, ethical issues and future forthcoming. J Prev Med Hyg. 2024;64(4):E398–E404. doi:10.15167/2421-4248/jpmh2023.64.4.3144
19. Smith AKJ, Storer D, Lancaster K, et al. Mpox illness narratives: stigmatising care and recovery during and after an emergency outbreak. Qual Health Res. 2024;34(12):1161–1174. doi:10.1177/10497323241234482
20. Takenaka BP, Kirklewski SJ, Griffith FJ, et al. “It’s another gay disease”: An intersectional qualitative approach contextualizing the lived experiences of young gay, bisexual, and other sexual minoritized men in the United States during the mpox outbreak. BMC Public Health. 2024;24:1574. doi:10.1186/s12889-024-19062-z
21. Witzel TC, Ghobrial A, Palich R, et al. Experiences of mpox illness and case management among cis and trans gay, bisexual and other men who have sex with men in England: a qualitative study. EClinicalMedicine. 2024;70:102522. doi:10.1016/j.eclinm.2024.102522
22. Biesty CP, Hemingway C, Woolgar J, et al. Community led health promotion to counter stigma and increase trust amongst priority populations: lessons from the 2022– 2023 UK mpox outbreak. BMC Public Health. 2024;24:1638. doi:10.1186/s12889-024- 19142-2
23. Logie CH. What can we learn from HIV, COVID-19 and mpox stigma to guide stigma-informed pandemic preparedness? J Int AIDS Soc. 2022;25:e26060. doi:10.1002/jia2.26060
24. Billings J, Ching BCF, Gkofa V, et al. Experiences of frontline healthcare workers and their views about support during COVID-19 and previous pandemics: a systematic review and qualitative meta-synthesis. BMC Health Serv Res. 2021;21(1):923. doi:10.1186/s12913-021-06917-z
25. Apea V, Titanji BK, Dakin FH, et al. International healthcare workers’ experiences and perceptions of the 2022 multi-country mpox outbreak. PLOS Glob Public Health. 2025;5(2):e0003704. doi:10.1371/journal.pgph.0003704
26. Bow SMA, Schröder-Bäck P, Norcliffe-Brown D, et al. Moral distress and injury in the public health professional workforce during the COVID-19 pandemic. J Public Health (Oxf). 2023;45(3):697–705. doi:10.1093/pubmed/fdad010
27. Jahromi AS, Jokar M, Sharifi N, et al. Global knowledge and attitudes towards mpox (monkeypox) among healthcare workers: a systematic review and meta-analysis. Int Health. 2024;16:487-498. doi:10.1093/inthealth/ihad107
28. West CP, Dyrbye LN, Sinsky C, et al. Resilience and burnout among physicians and the general US working population. JAMA Netw Open. 2020;3(7):e209385. doi:10.1001/jamanetworkopen.2020.9385
29. Hussein M, et al. Ethical considerations during Mpox Outbreak: a scoping review.

BMC Med Ethics. 2024;25:78. doi:10.1186/s12910-024-01080-4

30. May T, Towler L, Smith LE, et al. Mpox knowledge, behaviours and barriers to public health measures among gay, bisexual and other men who have sex with men in the UK: a qualitative study to inform public health guidance and messaging. BMC Public Health. 2023;23(1):2265. doi:10.1186/s12889-023-17134-4
31. Torres TS, Silva MST, Coutinho C, et al. Evaluation of Mpox Knowledge, Stigma, and willingness to Vaccinate for Mpox: cross-sectional web-based survey among sexual and gender minorities. JMIR Public Health Surveill. 2023;9:e46489. doi:10.2196/46489
32. McLeroy KR, Bibeau D, Steckler A, Glanz K. An ecological perspective on health promotion programs. Health Educ Q. 1988;15(4):351-377. doi:10.1177/109019818801500401
33. Gale NK, Heath G, Cameron E, Rashid S, Redwood S. Using the framework method for the analysis of qualitative data in multi-disciplinary health research. BMC Med Res Methodol. 2013;13:117. doi:10.1186/1471-2288-13-117
34. Ritchie J, Spencer L. Qualitative data analysis for applied policy research. In: Bryman A, Burgess RG, editors. Analyzing qualitative data. London: Routledge; 1994. p. 173– 194.
35. Pope C, Ziebland S, Mays N. Qualitative research in health care: Analysing qualitative data. BMJ. 2000;320(7227):114-116. doi:10.1136/bmj.320.7227.114
36. May CR, Johnson M, Finch T. Implementation, context and complexity. Implement Sci. 2016;11:141. doi:10.1186/s13012-016-0506-3
37. Tong A, Sainsbury P, Craig J. Consolidated criteria for reporting qualitative research (COREQ): a 32-item checklist for interviews and focus groups. Int J Qual Health Care. 2007;19:349-357. doi:10.1093/intqhc/mzm042
38. Schmalzle SA, Grant M, Lovelace S, et al. Survey of pain and stigma experiences in people diagnosed with mpox in Baltimore, Maryland during 2022 global outbreak. PLoS ONE. 2024;19(5):e0299587. doi:10.1371/journal.pone.0299587
39. Bury M. Chronic illness as biographical disruption. Sociol Health Illn. 1982;4:167-

182. doi:10.1111/1467-9566.ep11339939

40. Hatzenbuehler ML, Phelan JC, Link BG. Stigma as a fundamental cause of population health inequalities. Am J Public Health. 2013;103:813-821. doi:10.2105/AJPH.2012.301069
41. Paterson A, Cheyne A, Jones B, et al. An hourglass model for conceptualising stigma in infectious disease outbreaks. Sci Rep. 2025;15:15339. doi:10.1038/s41598-025-98995- w
42. Betsch C, Wieler LH, Habersaat K. Monitoring behavioural insights related to COVID-19. Lancet. 2020;395(10232):1255-1256. doi:10.1016/S0140-6736(20)30729-7

# Supporting information

**S1 Appendix. Framework description and thematic framework summary.** Integrated SEM+HSDF analytical framework, S1 Fig thematic framework diagram, and thematic framework overview.

**S2 Appendix. Semi-structured interview guides.** Interview guides for survivors, healthcare workers, community members, and contact tracers.

**S3 Appendix. Focus group discussion guide.** Guide for focus group discussions with healthcare workers, community members, and contact tracers.

**S4 Appendix. Coding framework.** Full codebook with code names, definitions, inclusion/exclusion criteria, and example excerpts.

**S5 Appendix. COREQ checklist.** Completed the Consolidated Criteria for Reporting Qualitative Research checklist.

**S6 Appendix. Extended participant quotations by theme.** Additional quotations supporting each theme and subtheme.

<https://www2.cloud.editorialmanager.com/pgph/download.aspx?id=719636&guid=dfc0b14c-3b52-4def-88ac-b9d1af70ee94&scheme=1>Figure	[Click here to access/download;Figure;S1](https://www2.cloud.editorialmanager.com/pgph/download.aspx?id=719636&guid=dfc0b14c-3b52-4def-88ac-b9d1af70ee94&scheme=1)

[Fig_Thematic_Framework.docx](https://www2.cloud.editorialmanager.com/pgph/download.aspx?id=719636&guid=dfc0b14c-3b52-4def-88ac-b9d1af70ee94&scheme=1)

### S1 Fig. Thematic Framework: Mpox Outbreak Experiences in Sierra Leone

Supporting Information

[Click here to access/download](https://www2.cloud.editorialmanager.com/pgph/download.aspx?id=719638&guid=c5bf7391-0b53-4a5b-97f6-326e0263d7ff&scheme=1)

[**Supporting Information** mpox_PLOS_supplementary.docx](https://www2.cloud.editorialmanager.com/pgph/download.aspx?id=719638&guid=c5bf7391-0b53-4a5b-97f6-326e0263d7ff&scheme=1)
