# Supplementary material for: Suffering in silence: Stigma, healthcare barriers, and resilience during Sierra Leone’s 2025 clade IIb mpox outbreak—A multi-perspective qualitative study
Source: PLOS Glob Public Health. 2026 Jun 30;6(6):e0006686. doi: 10.1371/journal.pgph.0006686 (PMC13318003; doi:10.1371/journal.pgph.0006686)
Supplement: Attachment — Submitted filename: PGPH-D-26-00336_Response_to_Reviewers.docx [file pgph.0006686.s011.docx]

Response to Reviewers

PLOS Global Public Health | Manuscript ID: PGPH-D-26-00336

Suffering in silence: Stigma, healthcare barriers, and resilience during Sierra Leone’s 2025 clade IIb mpox outbreak—A multi-perspective qualitative study

Date: 8 May 2026

Dear Dr. Bloom and Members of the Editorial Team,

We thank you and the reviewers for the careful consideration given to our manuscript and for the constructive comments. We are grateful that Reviewer 1 found the manuscript well written and methodologically sound, and that Reviewer 2 described the study as strong, well-executed, timely, and methodologically robust.

We revised the manuscript to address each journal requirement and each reviewer comment. In particular, we clarified the relationship between our sensitising framework and inductive findings; strengthened the conceptual framing of compound trauma; clarified the rationale for healthcare worker focus group discussions and the limits of those data; removed the risk of over-interpreting subgroup tables by presenting subgroup observations narratively; added a transparent limitation on sexual transmission and underreporting; expanded Theme 5 on health system response, vaccination, and coordination; and clarified the local leadership and authorship structure of the study. All changes are visible in the revised manuscript with changes highlighted.

# Responses to Journal Requirements

## Journal Requirement 1: Inclusivity in Global Research Questionnaire

**Response:**

We thank the editorial team for this request and for the journal's commitment to transparency, equity, and accountability in global health research. We completed the PLOS Inclusivity in Global Research Questionnaire and uploaded it as Supporting Information. We also revised the manuscript's Research team and reflexivity section and added a brief Methods subsection directing readers to the completed checklist.

This study was conceived, approved, implemented, analysed, and interpreted from within Sierra Leone’s public health system and under the institutional mandate of the National Public Health Agency (NPHA). The study was not organised as an externally led research project; it arose from and remained accountable to Sierra Leone’s national public health response structures.

The author team includes senior Sierra Leonean public health leadership and surveillance expertise. Dr. Eric Nzirakaindi Ikoona, the corresponding author and first author, works with the NPHA in Freetown, Sierra Leone, as strategic advisor for research and evaluation where he has served for eight years following doctoral training. His post-doctoral public health practice, outbreak response work, and research leadership are embedded within Sierra Leone's national public health system. Professor Foday Sahr is a senior research professor and Executive Director of the NPHA; Dr Mohamed Alex Vandi is a Sierra Leonean physician and Deputy Executive Director of the NPHA; and Mary Magdalene Sinnah is a Sierra Leonean Principal Surveillance Officer. Their roles brought direct knowledge of Sierra Leone's outbreak response system, surveillance architecture, institutional context, and community-facing public health practice.

Dr. Lucy Namulemo was included for her specialist expertise as a PhD-trained researcher in qualitative research and mental health; her role strengthened the methodological and psychosocial dimensions of the study and did not replace local leadership.

All eight research assistants were Sierra Leoneans, embedded in Sierra Leonean cultural contexts, and collectively fluent in the local languages used for the study. They conducted data collection in English, Krio, Mende, or Temne according to participant preference. The study was therefore locally led, institutionally grounded, and accountable to Sierra Leone's public health system and communities.

## Journal Requirement 2: Data Availability

**Response:**

We respectfully request a targeted exemption from public deposition of full interview and focus group transcripts. The transcripts contain sensitive qualitative data from a stigmatising infectious disease outbreak. Even after de-identification, they include indirect identifiers related to geography, household relationships, illness trajectories, health facility encounters, occupational roles, and outbreak response activities. Public release could create a credible risk of deductive identification, especially in districts with small numbers of cases or specialised response roles.

To support transparency and reproducibility, we provide substantial study materials as Supporting Information: interview and focus group guides, the full coding framework with inclusion and exclusion criteria, the completed COREQ checklist, and extended de-identified quotations mapped to themes and subthemes. Additional de-identified excerpts may be requested from the corresponding author under a data-sharing agreement, subject to conditions consistent with Sierra Leone Ethics and Scientific Review Committee approval.

## Journal Requirement 3: Ethics Statement Placement

**Response:**

We moved the Ethics Statement to the beginning of the Methods section under the subheading “Ethics Statement,” as required. We retained fuller procedural detail later in the Methods section under participant confidentiality and support procedures.

## Journal Requirement 4: Figure Files

**Response:**

We prepared a separate TIFF figure file and processed it through the PLOS NAAS tool. The validated file is submitted with this revision.

## Journal Requirement 5: Title Consistency

**Response:**

We revised the manuscript title to match the title used in the online submission form: “Suffering in silence: Stigma, healthcare barriers, and resilience during Sierra Leone’s 2025 clade IIb mpox outbreak—A multi-perspective qualitative study.”

## Journal Requirement 6: Supporting Information Legends

**Response:**

We added a full list of Supporting Information legends after the References section and removed attachment-placeholder text from the manuscript file.

# Reviewer 1: Dr. Chizaram Onyeaghala

We thank Reviewer 1 for the generous and constructive assessment, and for recognising the importance of stigma and psychosocial challenges in mpox response. We accepted the reviewer’s tracked edits and corrected the abbreviation, typographical, and wording issues identified in the annotated manuscript. We also reviewed the manuscript for similar errors and corrected repeated definitions of abbreviations after first use, wording duplication, and minor syntax issues.

# Reviewer 2: Anonymous

We thank Reviewer 2 for the careful and intellectually rigorous critique. The six major comments substantially improved the manuscript. We respond to each point below.

## Comment 1: Stigma embedded in the analytical framework

**Reviewer comment:**

Stigma was embedded in the analytical framework prior to data collection but is then presented as an emergent finding. This is not a trivial issue. It should be explicitly acknowledged as a limitation. More importantly, the authors should be clear about what did emerge inductively—what findings challenged, extended, or complicated the initial framework?

**Response:**

We agree. We revised the Methods, Results, Discussion, and Limitations sections to distinguish between framework-informed sensitising concepts and inductive findings. The revised manuscript now states that stigma was anticipated by the HSDF and should not be presented as a wholly inductive discovery. What emerged inductively was the recursive movement of stigma across illness experience, care-seeking, professional practice, contact tracing, and system response. We also identified inductive extensions to the framework: institutional stigma directed toward treatment centres, associative stigma toward contact tracers, religious and moral idioms of internalised stigma, informal stigma-mitigation practices used by response teams, and the cyclical relationship between enacted stigma, anticipated rejection, concealment, and community suspicion.

## Comment 2: Compound trauma underdeveloped

**Reviewer comment:**

The concept of “compound trauma” among HCWs with prior Ebola and COVID-19 experience is arguably the most novel contribution of the paper. It is well described in the results and flagged in the discussion, but it is underdeveloped conceptually. The current framing leans on burnout and moral distress literature, which is not sufficient. There is a substantial body of work on cumulative trauma, moral injury, and sequential disaster exposure that is not meaningfully engaged here. If this is intended as a novel empirical contribution it needs a stronger theoretical anchor and a more prominent position in the conclusions.

**Response:**

We agree. We strengthened the conceptual discussion of compound trauma by distinguishing it from burnout and linking it to cumulative trauma, moral injury, and sequential disaster exposure. The revised manuscript now defines compound trauma as the reactivation and accumulation of distress across discrete epidemic events within the same professional life course. We added relevant references on moral injury and disaster mental health and gave the concept a more prominent place in the Conclusions. A companion manuscript examining compound trauma in depth within the Sierra Leone healthcare workforce context is currently in preparation. The present paper stands independently on the conceptual grounding provided in the revised Discussion.

## Comment 3: HCW FGD rationale and silencing dynamics

**Reviewer comment:**

Survivors were interviewed individually, which is appropriate given the sensitivity and stigma associated with their experiences. However, HCWs participated in both SSIs and FGDs, and the rationale for this is not clearly articulated. FGDs in hierarchical clinical settings raise real concerns about suppressed disclosure. The authors should clarify whether groups were stratified by role or seniority, and whether any silencing dynamics were observed or actively mitigated.

**Response:**

We agree. We revised the Data Collection and Limitations sections. The manuscript now explains that healthcare worker SSIs were used for sensitive individual accounts, including moral distress and emotional burden, while FGDs were used to explore shared operational experiences and collective reflections on service delivery, coordination, and facility-level challenges. We clarified that groups were organised by stakeholder type and district, and that hierarchy was reduced where feasible, but full stratification by cadre or seniority was not always possible because of small eligible staff numbers in some facilities. We also added mitigation steps: facilitators monitored group dynamics, invited quieter participants to speak, normalised disagreement, warned against identifying colleagues or patients, and offered individual follow-up where feasible. We explicitly acknowledge that some suppression of disclosure may still have occurred.

## Comment 4: Subgroup presentation in Tables 2 and 3

**Reviewer comment:**

Tables 2 and 3 present subgroup variation and risk over-interpretation. Some subgroups, such as Kenema district survivors, are too small to support even cautious patterning. The authors should consider whether these tables add sufficient analytical value or whether these observations would be better integrated narratively.

**Response:**

We agree. We removed Tables 2 and 3 from the revised manuscript and integrated the subgroup observations narratively. The revised text now explicitly states that these observations are exploratory analytic prompts, not generalisable subgroup differences. We retained only cautious narrative observations around gender, age, urban/rural residence, employment, and care-seeking context, with clear warnings about small subgroup sizes.

## Comment 5: Sexual transmission and underreporting

**Reviewer comment:**

The paper is notably cautious around sexual transmission, despite genomic and epidemiologic signals and clear stigma tied to perceived sexual exposure. The authors should address whether relevant experiences were likely underreported and discuss this transparently as a limitation.

**Response:**

We agree. We revised the Introduction and Limitations sections to address this transparently and carefully. The manuscript now states that genital involvement, clade IIb epidemiology, and participant accounts linking mpox to shame, intimacy, and perceived sexual exposure suggest that sexual transmission concerns shaped stigma and disclosure. We added a limitation stating that sexual exposures and partner-related experiences were likely underreported because of stigma, fear of disclosure, and the social risks attached to discussing sexual behaviour in this setting. We make no claims about the actual transmission routes of individual participants.

## Comment 6: Theme 5 underdeveloped

**Reviewer comment:**

Theme 5 (“Building the plane while flying”) is underdeveloped relative to the earlier themes. Health system response, particularly vaccination rollout and coordination challenges, is highly policy-relevant and should be expanded with more granular detail and analytic depth.

**Response:**

We agree. We expanded Theme 5 substantially. The revised section now includes adaptive capacity and local improvisation, coordination and information flow, vaccination rollout and trust, and contact tracing barriers. We added detail on treatment capacity expansion, referral coordination, informal communication networks, bed and transport constraints, data-sharing challenges, vaccination prioritisation, cold-chain and scheduling issues, community uncertainty about eligibility, and how stigma shaped cooperation with contact tracing and vaccination. We also strengthened the policy implications for health system preparedness in post-conflict, multi-epidemic settings.

We trust that these revisions address the editor’s requirements and the reviewers’ comments. We are grateful for the opportunity to revise the manuscript and submit it for further consideration.

Yours sincerely,

Dr. Eric Nzirakaindi Ikoona, MD, MMed, PhD, MPH

Corresponding Author

Strategic Adviser for Research and Evaluation, National Public Health Agency

Freetown, Sierra Leone

ikoonae@yahoo.com

ORCID: 0000-0003-3402-1961

On behalf of Lucy Namulemo, Mary Magdalene Sinnah, Mohamed Alex Vandi, and Foday Sahr
