# Supplementary material for: Suffering in silence: Stigma, healthcare barriers, and resilience during Sierra Leone’s 2025 clade IIb mpox outbreak—A multi-perspective qualitative study
Source: PLOS Glob Public Health. 2026 Jun 30;6(6):e0006686. doi: 10.1371/journal.pgph.0006686 (PMC13318003; doi:10.1371/journal.pgph.0006686)
Supplement: Peer review history

# Peer review: Decision Letter 0

**DOI:** 10.1371/journal.pgph.0006686.r001

**Authors:** Ilse Bloom

8 May 2026

PGPH-D-26-00336

Suffering in silence: Stigma, healthcare barriers, and resilience during Sierra Leone's 2025 clade IIb mpox outbreak—A multi-perspective qualitative study

PLOS Global Public Health

Dear Dr. Ikoona,

Thank you for submitting your manuscript to PLOS Global Public Health. After careful consideration, we feel that it has merit but does not fully meet PLOS Global Public Health’s publication criteria as it currently stands. Therefore, we invite you to submit a revised version of the manuscript that addresses the points raised during the review process.

The manuscript has been evaluated by two reviewers, and their comments are available below.

Could you please carefully revise the manuscript to address all comments raised?

Please submit your revised manuscript by Jul 06 2026 11:59PM. If you will need more time than this to complete your revisions, please reply to this message or contact the journal office at globalpubhealth@plos.org. When you're ready to submit your revision, log on to https://www.editorialmanager.com/pgph/ and select the 'Submissions Needing Revision' folder to locate your manuscript file.

Please include the following items when submitting your revised manuscript:

- A letter that responds to each point raised by the editor and reviewer(s). You should upload this letter as a separate file labeled 'Response to Reviewers'.
- A marked-up copy of your manuscript that highlights changes made to the original version. You should upload this as a separate file labeled 'Revised Manuscript with Track Changes'.
- An unmarked version of your revised paper without tracked changes. You should upload this as a separate file labeled 'Manuscript'.

Guidelines for resubmitting your figure files are available below the reviewer comments at the end of this letter.

As the corresponding author, your ORCID iD is verified in the submission system and will appear in the published article. PLOS supports the use of ORCID, and we encourage all coauthors to register for an ORCID iD and use it as well. Please encourage your coauthors to verify their ORCID iD within the submission system before final acceptance, as unverified ORCID iDs will not appear in the published article. *Only*  the individual author can complete the verification step; PLOS staff *cannot*  verify ORCID iDs on behalf of authors.

We look forward to receiving your revised manuscript.

Kind regards,

Ilse Bloom

Staff Editor

PLOS Global Public Health

**Journal Requirements:**

1. Please include a complete copy of PLOS’ questionnaire on inclusivity in global research in your revised manuscript. Our policy for research in this area aims to improve transparency in the reporting of research performed outside of researchers’ own country or community. The policy applies to researchers who have travelled to a different country to conduct research, research with Indigenous populations or their lands, and research on cultural artefacts. The questionnaire can also be requested at the journal’s discretion for any other submissions, even if these conditions are not met. Please find more information on the policy and a link to download a blank copy of the questionnaire here: https://journals.plos.org/globalpublichealth/s/best-practices-in-research-reporting. Please upload a completed version of your questionnaire as Supporting Information when you resubmit your manuscript.

2. In the online submission form, you indicated that “De-identified qualitative data (full transcripts) cannot be publicly shared because they contain sensitive information and indirect identifiers related to a stigmatizing infectious disease outbreak context, and public release may risk participant re-identification given the small number of mpox cases in specific geographic areas during the study period. The study materials that support transparency and reproducibility are available in the Supporting Information files: interview and focus group discussion guides (S2–S3 Appendix), the full coding framework with inclusion/exclusion criteria (S4 Appendix), the completed COREQ checklist (S5 Appendix), and extended de-identified quotations mapped to themes (S6 Appendix). Researchers may request access to further de-identified data excerpts under a data-sharing agreement, subject to approval by the Sierra Leone Ethics and Scientific Review Committee. Requests should be directed to the corresponding author (Eric Nzirakaindi Ikoona, ikoonae@yahoo.com) with a brief proposal describing the intended use and data protection safeguards.”.

All PLOS journals now require all data underlying the findings described in their manuscript to be freely available to other researchers, either

1. In a public repository,

2. Within the manuscript itself, or

3. Uploaded as supplementary information.

This policy applies to all data except where public deposition would breach compliance with the protocol approved by your research ethics board. If your data cannot be made publicly available for ethical or legal reasons (e.g., public availability would compromise patient privacy), please explain your reasons by return email and your exemption request will be escalated to the editor for approval. Your exemption request will be handled independently and will not hold up the peer review process, but will need to be resolved should your manuscript be accepted for publication. One of the Editorial team will then be in touch if there are any issues.

3. Please ensure that your Ethics Statement is available in its entirety at the beginning of your Methods section, under a subheading 'Ethics Statement'.

4. Please provide separate figure files in .tif or .eps format.

For more information about figure files please see our guidelines:

https://journals.plos.org/globalpublichealth/s/figures

https://journals.plos.org/globalpublichealth/s/figures#loc-file-requirements

5. Please ensure that the Title in your manuscript file and the Title provided in your online submission form are the same.

6. We have noticed that you have uploaded Supporting Information files, but you have not included a list of legends. Please add a full list of legends for your Supporting Information files after the references list.

If the reviewer comments include a recommendation to cite specific previously published works, please review and evaluate these publications to determine whether they are relevant and should be cited. There is no requirement to cite these works unless the editor has indicated otherwise.

**Additional Editor Comments (if provided):**

[Note: HTML markup is below. Please do not edit.]

Reviewers' comments:

Reviewer's Responses to Questions

**Comments to the Author**

1. Does this manuscript meet PLOS Global Public Health’s publication criteria? Is the manuscript technically sound, and do the data support the conclusions? The manuscript must describe methodologically and ethically rigorous research with conclusions that are appropriately drawn based on the data presented.

Reviewer #1: Yes

Reviewer #2: Yes

**********

2. Has the statistical analysis been performed appropriately and rigorously?

Reviewer #1: Yes

Reviewer #2: Yes

**********

3. Have the authors made all data underlying the findings in their manuscript fully available (please refer to the Data Availability Statement at the start of the manuscript PDF file)?

The PLOS Data policy requires authors to make all data underlying the findings described in their manuscript fully available without restriction, with rare exception. The data should be provided as part of the manuscript or its supporting information, or deposited to a public repository. For example, in addition to summary statistics, the data points behind means, medians and variance measures should be available. If there are restrictions on publicly sharing data—e.g. participant privacy or use of data from a third party—those must be specified.

Reviewer #1: Yes

Reviewer #2: Yes

**********

4. Is the manuscript presented in an intelligible fashion and written in standard English?

PLOS Global Public Health does not copyedit accepted manuscripts, so the language in submitted articles must be clear, correct, and unambiguous. Any typographical or grammatical errors should be corrected at revision, so please note any specific errors here.

Reviewer #1: Yes

Reviewer #2: Yes

**********

5. Review Comments to the Author

Please use the space provided to explain your answers to the questions above. You may also include additional comments for the author, including concerns about dual publication, research ethics, or publication ethics. (Please upload your review as an attachment if it exceeds 20,000 characters)

**Reviewer #1** : Let me first of all thank the authors for addressing a crucial and under-explored area of the 2024-2026 mpox upsurge in Africa. Indeed, Stigma and other psychosocial challenges are real challenges that, if not addressed from the outset with tangible and tailored interventions, can effectively undermine response and prevention efforts.

The manuscript was well written, and adequate statistical rigor, ethical principles, and a detailed methodological application were employed in conducting this research in the outbreak areas in Sierra Leone.

Additionally, the authors described themselves as having the requisite experience needed to conduct this type of research.

Other aspects of the paper, including the discussion, limitations and a brief of the studys strength were aptly written.

However, I have provided minimal edits and corrections for the authors to consider as I am of the view that it will further improve the overall quality of the manuscript to meet the standards required of PLoS Global Public Health.

**Reviewer #2:**  This is a strong, well-executed and timely qualitative study addressing stigma, health worker barriers, and resilience during the 2025 mpox outbreak in Sierra Leone. The multi-stakeholder design integrating survivors, HCWs, community members, and contact tracers within a single analytical framework is a clear strength. It is methodologically sound and allows for a level of triangulation that single-group studies cannot achieve. Importantly, the literature remains sparse when it comes to African HCW and survivor experiences in this space, and this study helps to fill that gap.

I have several comments for the authors to consider:

Major comments

1. I am concerned that stigma was embedded in the analytical framework prior to data collection but is then presented as an emergent finding. That is not a trivial issue. It should be explicitly acknowledged as a limitation. More importantly, the authors should be clear about what did emerge inductively what findings challenged, extended, or complicated the initial framework?

2. The concept of “compound trauma” among HCWs with prior Ebola and COVID-19 experience is arguably the most novel contribution of the paper. It is well described in the results and flagged in the discussion, but it is underdeveloped conceptually. The current framing leans on burnout and moral distress literature, which is not sufficient. There is a substantial body of work on cumulative trauma, moral injury, and sequential disaster exposure that is not meaningfully engaged here. If this is intended as a novel empirical contribution and it appears to be needs a stronger theoretical anchor and a more prominent position in the conclusions.

3. Survivors were interviewed individually, which is appropriate given the sensitivity and stigma associated with their experiences. However, HCWs participated in both SSIs (n=28) and FGDs (n=4), and the rationale for this is not clearly articulated. FGDs in hierarchical clinical settings raise real concerns about suppressed disclosure, particularly around issues like moral distress, resource constraints, and colleague behavior. The authors should clarify whether groups were stratified by role or seniority, and whether any silencing dynamics were observed or actively mitigated.

4. Tables 2 and 3 present subgroup variation (gender, age, urban/rural) and are appropriately caveated as descriptive. That caveat is correct, but the presentation risks over-interpretation. Some subgroups e.g., Kenema district survivors (n=3) are too small to support even cautious patterning. The authors should consider whether these tables add sufficient analytical value relative to the risk of misinterpretation, or whether these observations would be better integrated narratively to emphasize their exploratory nature.

5. The paper is notably cautious around sexual transmission, despite genomic and epidemiologic signals (clade IIb, genital involvement, lineage A.2.2.1) and the clear presence of stigma tied to perceived sexual exposure in the findings. This is understandable given the legal and social context in Sierra Leone, but the absence of explicit acknowledgment is a gap. The authors should address whether relevant experiences were likely underreported and discuss this transparently as a limitation.

6. Theme 5 (“Building the plane while flying”) is underdeveloped relative to the earlier themes. This is a missed opportunity. Health system response particularly vaccination rollout and coordination challenges is highly policy-relevant, and African-context data in this area remain limited. This section should be expanded with more granular detail and analytic depth.

**********

6. PLOS authors have the option to publish the peer review history of their article (what does this mean?). If published, this will include your full peer review and any attached files.

**Do you want your identity to be public for this peer review?**  If you choose “no”, your identity will remain anonymous but your review may still be made public.

For information about this choice, including consent withdrawal, please see our Privacy Policy.

Reviewer #1: **Yes:** Chizaram Onyeaghala

Reviewer #2: No

**********

[NOTE: If reviewer comments were submitted as an attachment file, they will be attached to this email and accessible via the submission site. Please log into your account, locate the manuscript record, and check for the action link "View Attachments". If this link does not appear, there are no attachment files.]

**Figure Resubmissions:**

While revising your submission, we strongly recommend that you use PLOS’s NAAS tool (https://ngplosjournals.pagemajik.ai/artanalysis) to test your figure files. NAAS can convert your figure files to the TIFF file type and meet basic requirements (such as print size, resolution), or provide you with a report on issues that do not meet our requirements and that NAAS cannot fix.

After uploading your figures to PLOS’s NAAS tool - https://ngplosjournals.pagemajik.ai/artanalysis, NAAS will process the files provided and display the results in the "Uploaded Files" section of the page as the processing is complete. If the uploaded figures meet our requirements (or NAAS is able to fix the files to meet our requirements), the figure will be marked as "fixed" above. If NAAS is unable to fix the files, a red "failed" label will appear above. When NAAS has confirmed that the figure files meet our requirements, please download the file via the download option, and include these NAAS processed figure files when submitting your revised manuscript.

Attachment Submitted filename: PGPH-D-26-00336.docx

# Author comment: Author response to Decision Letter 1

**DOI:** 10.1371/journal.pgph.0006686.r002

14 May 2026

Attachment Submitted filename: PGPH-D-26-00336_Response_to_Reviewers.docx

# Peer review: Decision Letter 1

**DOI:** 10.1371/journal.pgph.0006686.r003

**Authors:** Ilse Bloom, Catherine Grant

2 Jun 2026

Suffering in silence: Stigma, healthcare barriers, and resilience during Sierra Leone's 2025 clade IIb mpox outbreak—A multi-perspective qualitative study

PGPH-D-26-00336R1

Dear Dr Ikoona,

We are pleased to inform you that your manuscript 'Suffering in silence: Stigma, healthcare barriers, and resilience during Sierra Leone's 2025 clade IIb mpox outbreak—A multi-perspective qualitative study' has been provisionally accepted for publication in PLOS Global Public Health.

Before your manuscript can be formally accepted you will need to complete some formatting changes, which you will receive in a follow up email. A member of our team will be in touch with a set of requests.

Please note that your manuscript will not be scheduled for publication until you have made the required changes, so a swift response is appreciated.

IMPORTANT: The editorial review process is now complete. PLOS will only permit corrections to spelling, formatting or significant scientific errors from this point onwards. Requests for major changes, or any which affect the scientific understanding of your work, will cause delays to the publication date of your manuscript.

If your institution or institutions have a press office, please notify them about your upcoming paper to help maximize its impact. If they'll be preparing press materials, please inform our press team as soon as possible -- no later than 48 hours after receiving the formal acceptance. Your manuscript will remain under strict press embargo until 2 pm Eastern Time on the date of publication. For more information, please contact globalpubhealth@plos.org.

Thank you again for supporting Open Access publishing; we are looking forward to publishing your work in PLOS Global Public Health.

Best regards,

Catherine Grant

Academic Editor

PLOS Global Public Health

***********************************************************

Reviewer Comments (if any, and for reference):

Reviewer's Responses to Questions

**Comments to the Author**

1. If the authors have adequately addressed your comments raised in a previous round of review and you feel that this manuscript is now acceptable for publication, you may indicate that here to bypass the “Comments to the Author” section, enter your conflict of interest statement in the “Confidential to Editor” section, and submit your "Accept" recommendation.

Reviewer #1: All comments have been addressed

Reviewer #2: All comments have been addressed

**********

2. Does this manuscript meet PLOS Global Public Health’s publication criteria? Is the manuscript technically sound, and do the data support the conclusions? The manuscript must describe methodologically and ethically rigorous research with conclusions that are appropriately drawn based on the data presented.

Reviewer #1: Yes

Reviewer #2: (No Response)

**********

3. Has the statistical analysis been performed appropriately and rigorously?

Reviewer #1: Yes

Reviewer #2: N/A

**********

4. Have the authors made all data underlying the findings in their manuscript fully available (please refer to the Data Availability Statement at the start of the manuscript PDF file)?

The PLOS Data policy requires authors to make all data underlying the findings described in their manuscript fully available without restriction, with rare exception. The data should be provided as part of the manuscript or its supporting information, or deposited to a public repository. For example, in addition to summary statistics, the data points behind means, medians and variance measures should be available. If there are restrictions on publicly sharing data—e.g. participant privacy or use of data from a third party—those must be specified.

Reviewer #1: Yes

Reviewer #2: Yes

**********

5. Is the manuscript presented in an intelligible fashion and written in standard English?

PLOS Global Public Health does not copyedit accepted manuscripts, so the language in submitted articles must be clear, correct, and unambiguous. Any typographical or grammatical errors should be corrected at revision, so please note any specific errors here.

Reviewer #1: Yes

Reviewer #2: (No Response)

**********

6. Review Comments to the Author

Please use the space provided to explain your answers to the questions above. You may also include additional comments for the author, including concerns about dual publication, research ethics, or publication ethics. (Please upload your review as an attachment if it exceeds 20,000 characters)

Reviewer #1: Thank you to the authors for substantially revising and addressing my initial comments.

They should address some typographical and grammatical irregularities in the attached manuscript, which will further improve the quality of the paper's readability for your audience.

Reviewer #2: I thank the authors for diligently addressing my queries

**********

7. PLOS authors have the option to publish the peer review history of their article (what does this mean?). If published, this will include your full peer review and any attached files.

**Do you want your identity to be public for this peer review?**  If you choose “no”, your identity will remain anonymous but your review may still be made public.

For information about this choice, including consent withdrawal, please see our Privacy Policy.

Reviewer #1: **Yes:** Chizaram Onyeaghala

Reviewer #2: No

**********

Attachment Submitted filename: PGPH-D-26-00336_R1.docx
